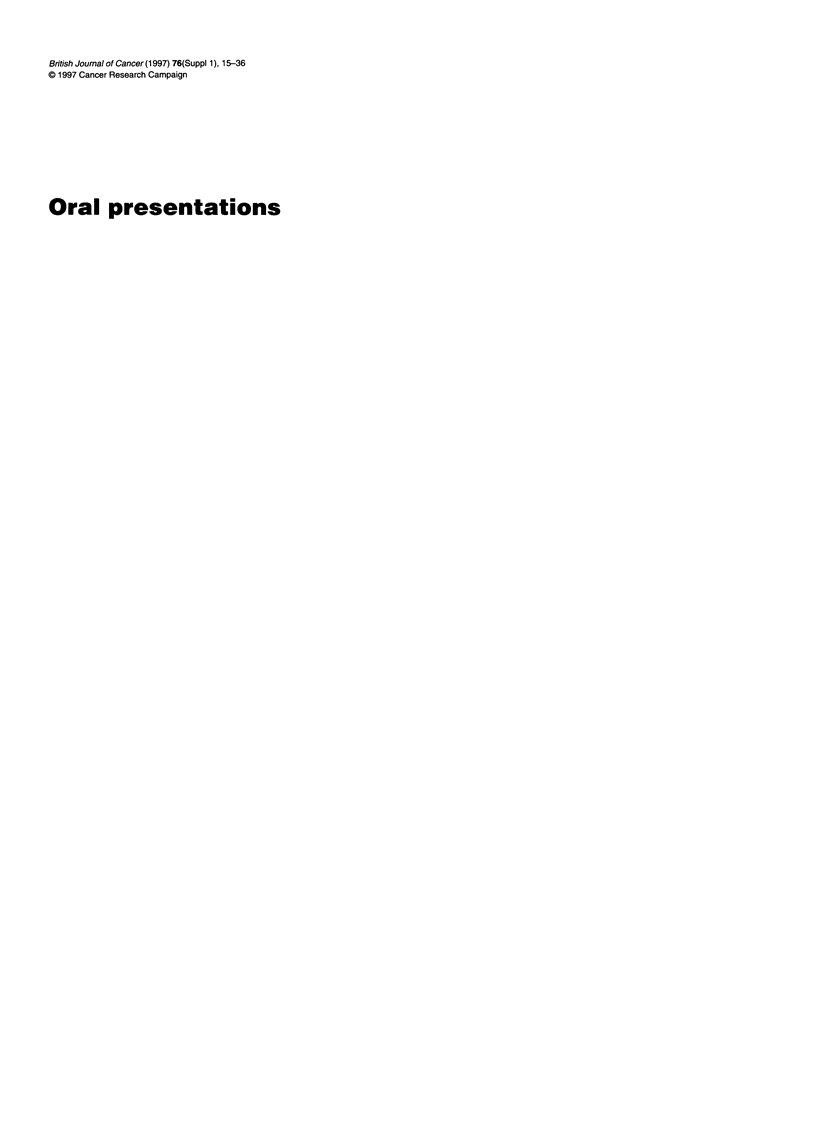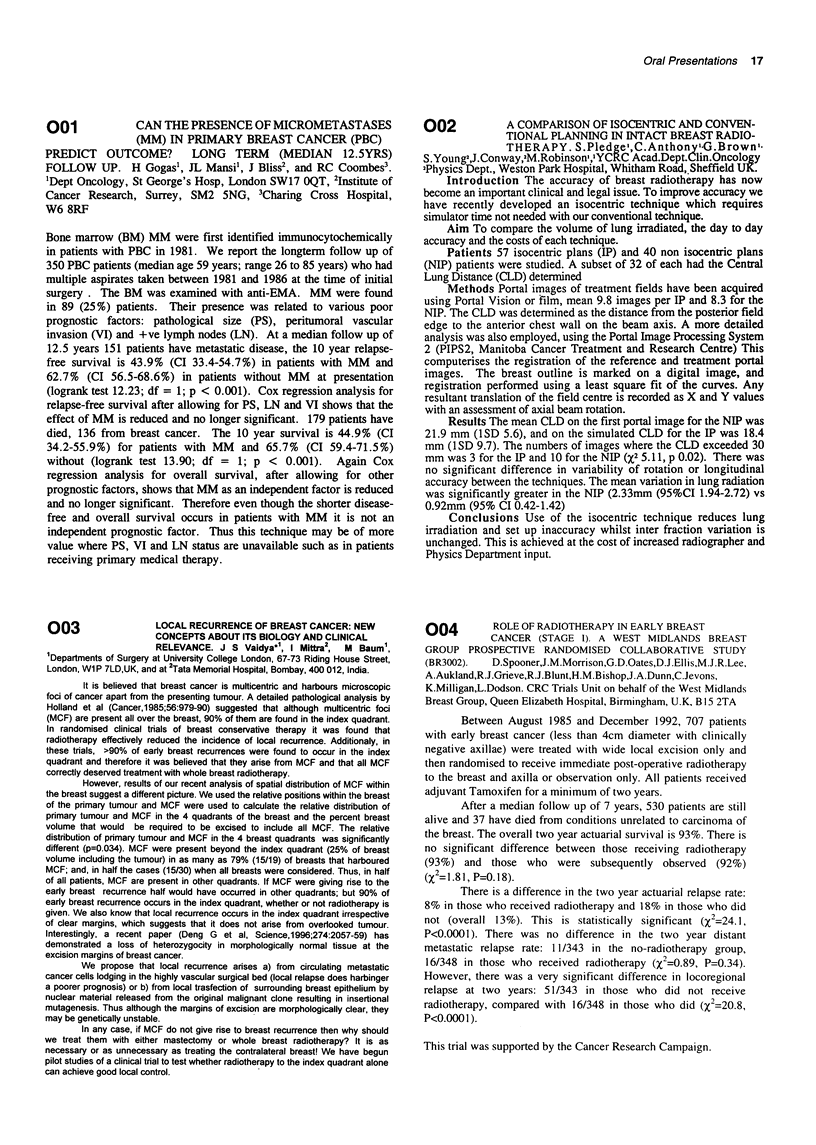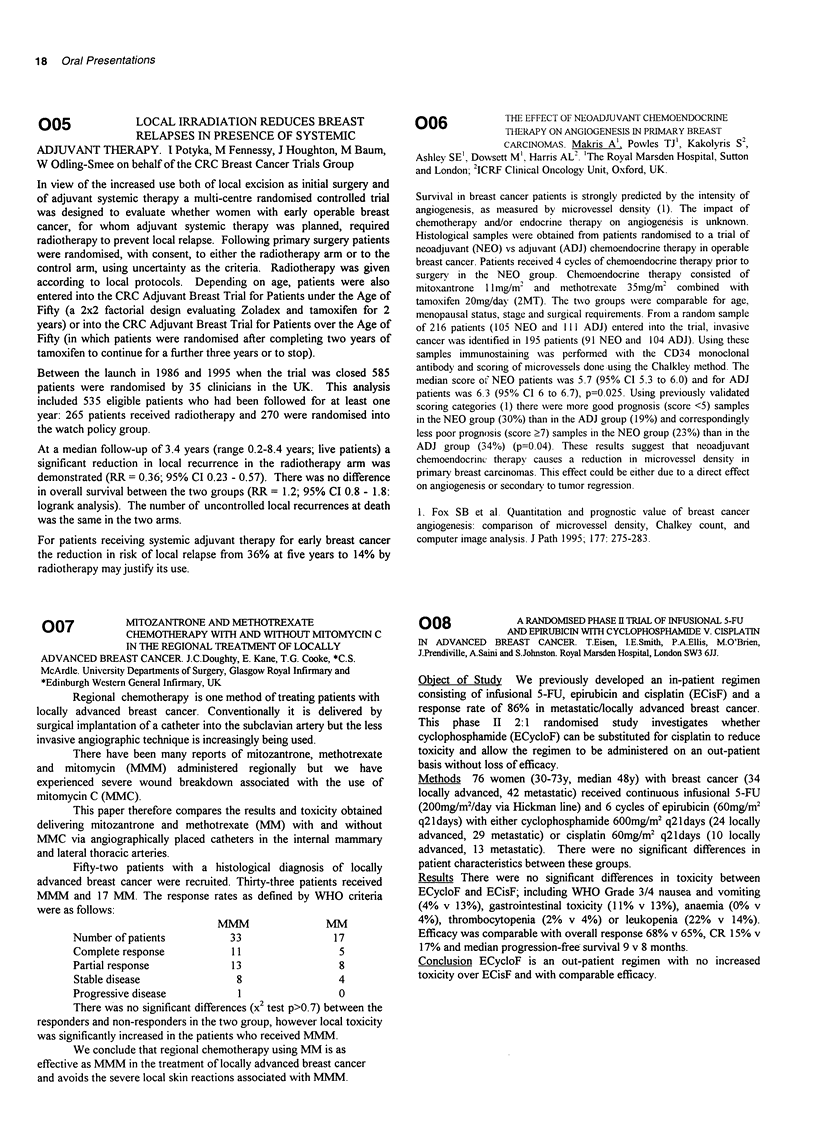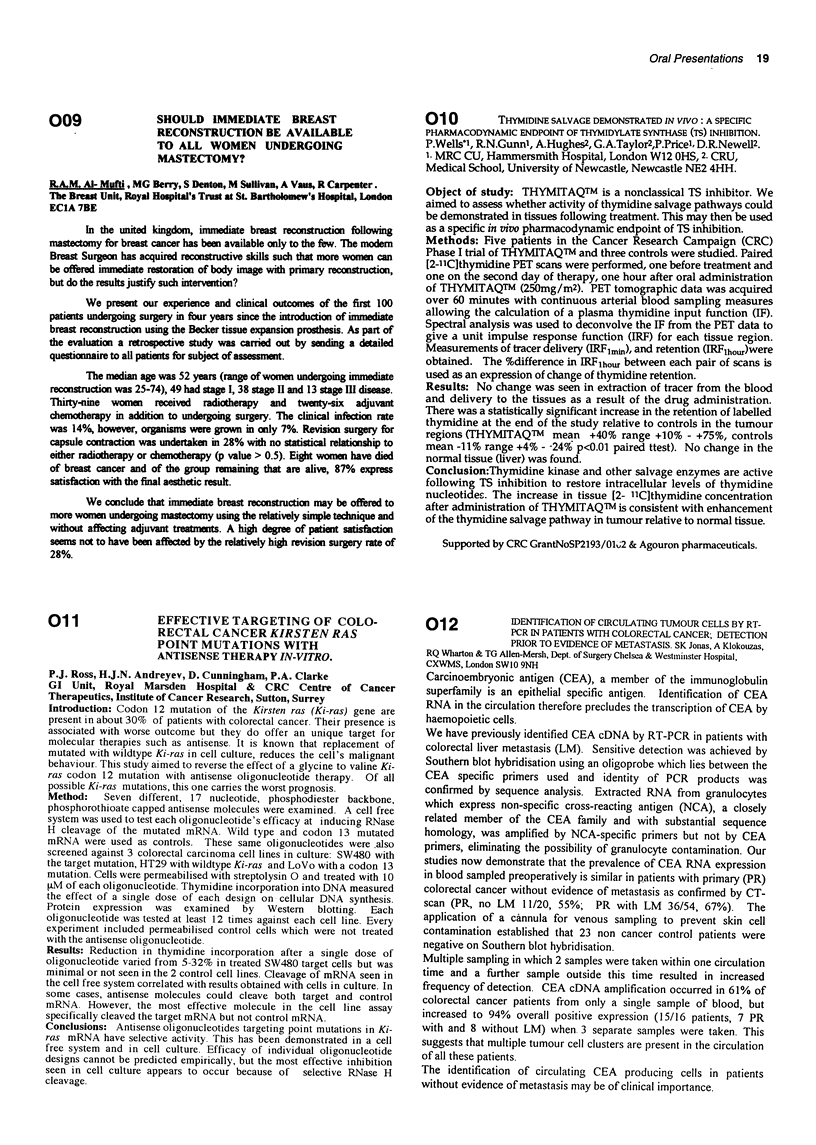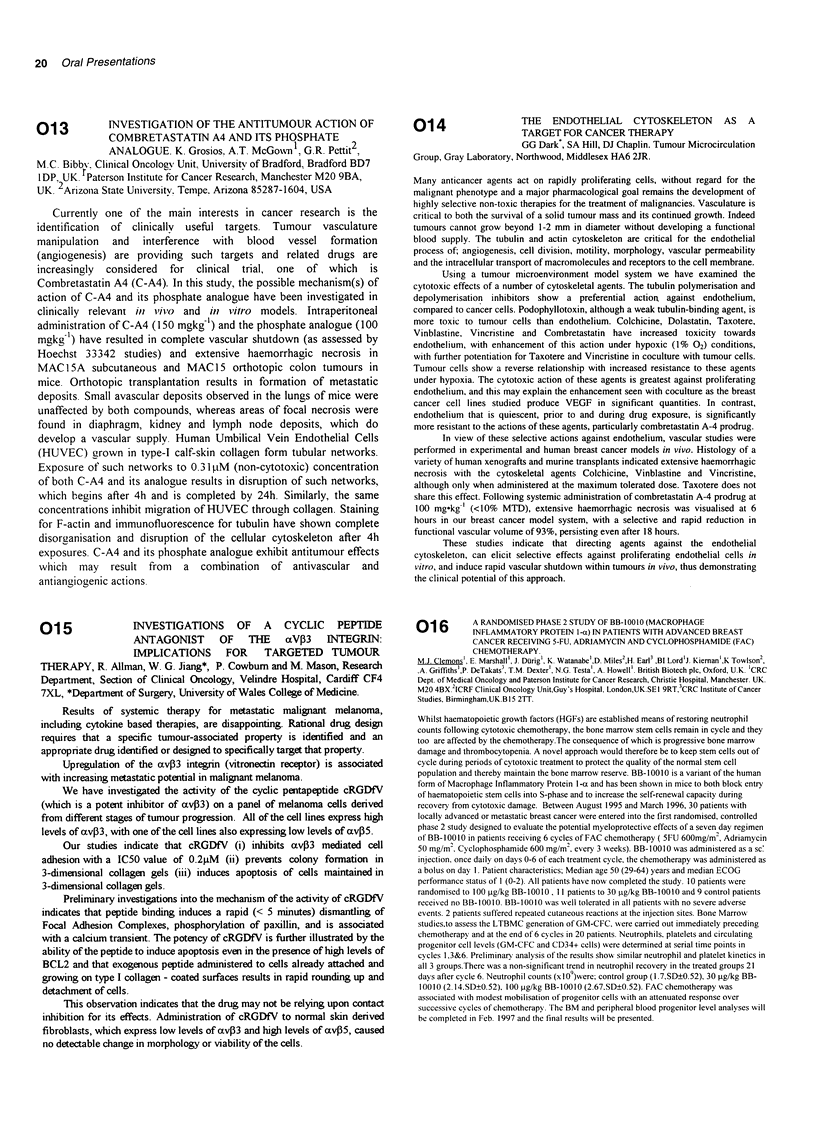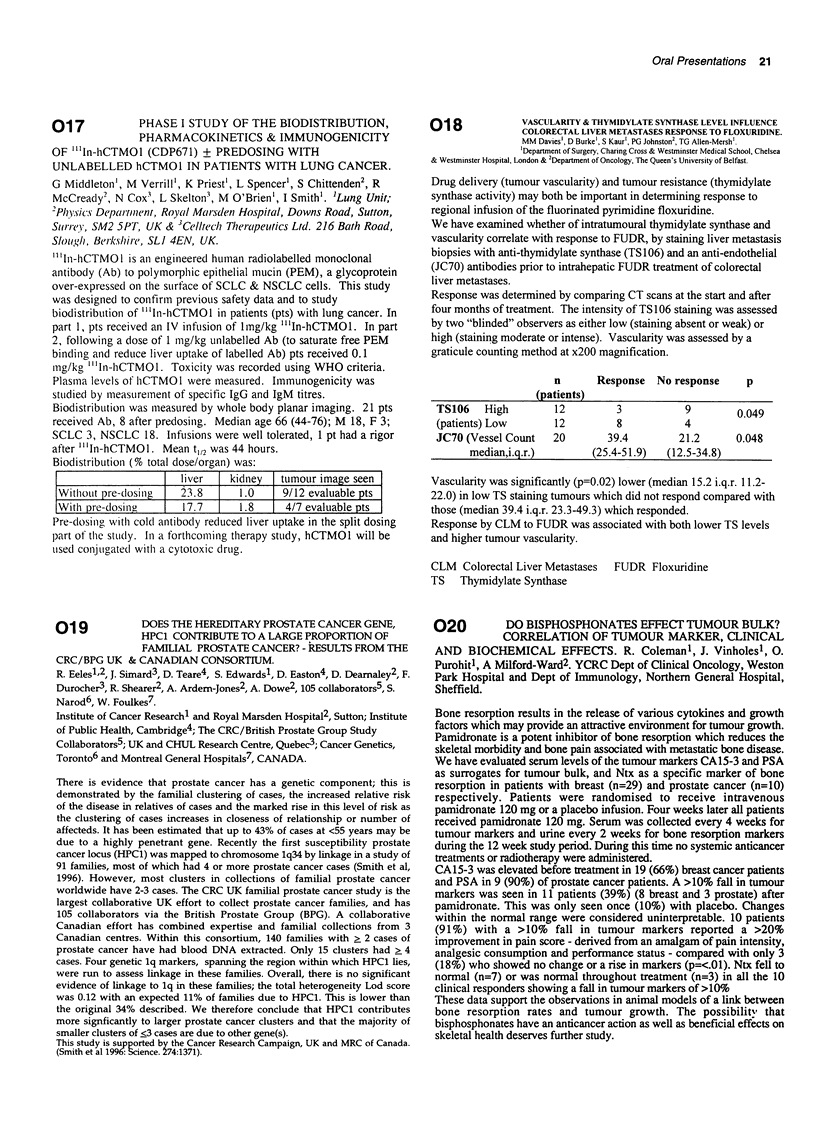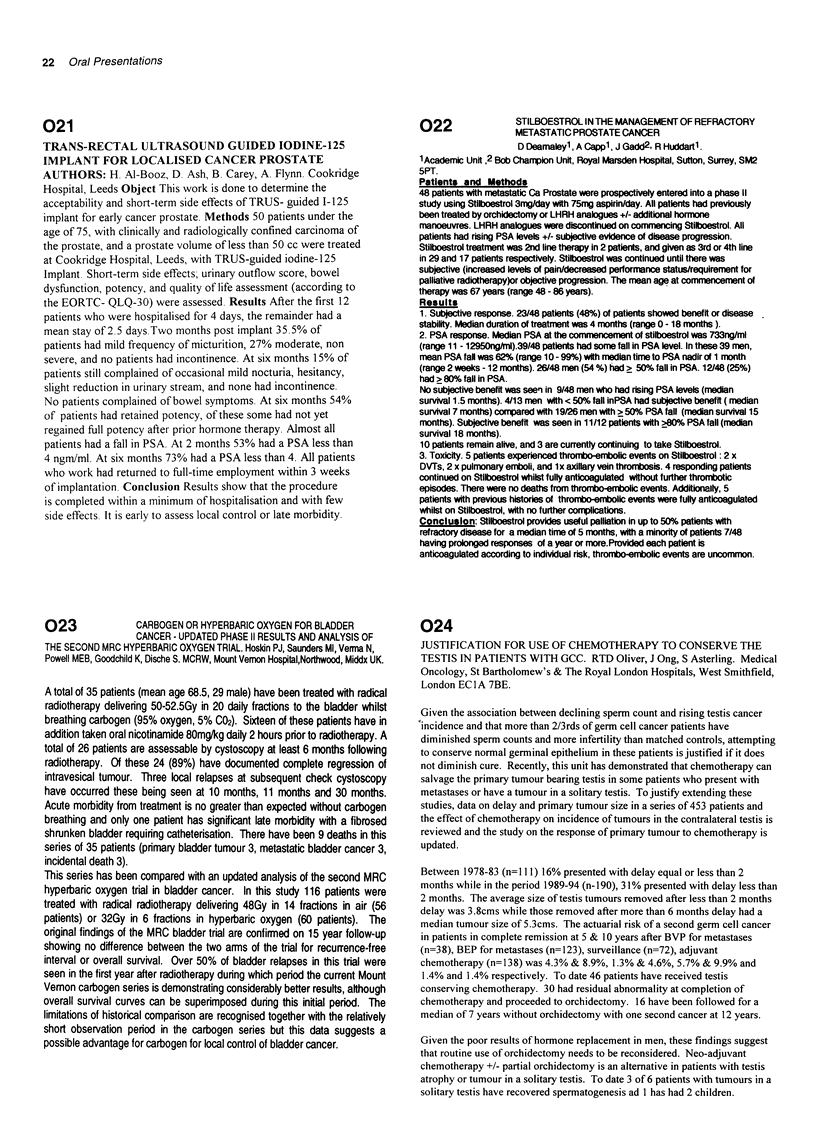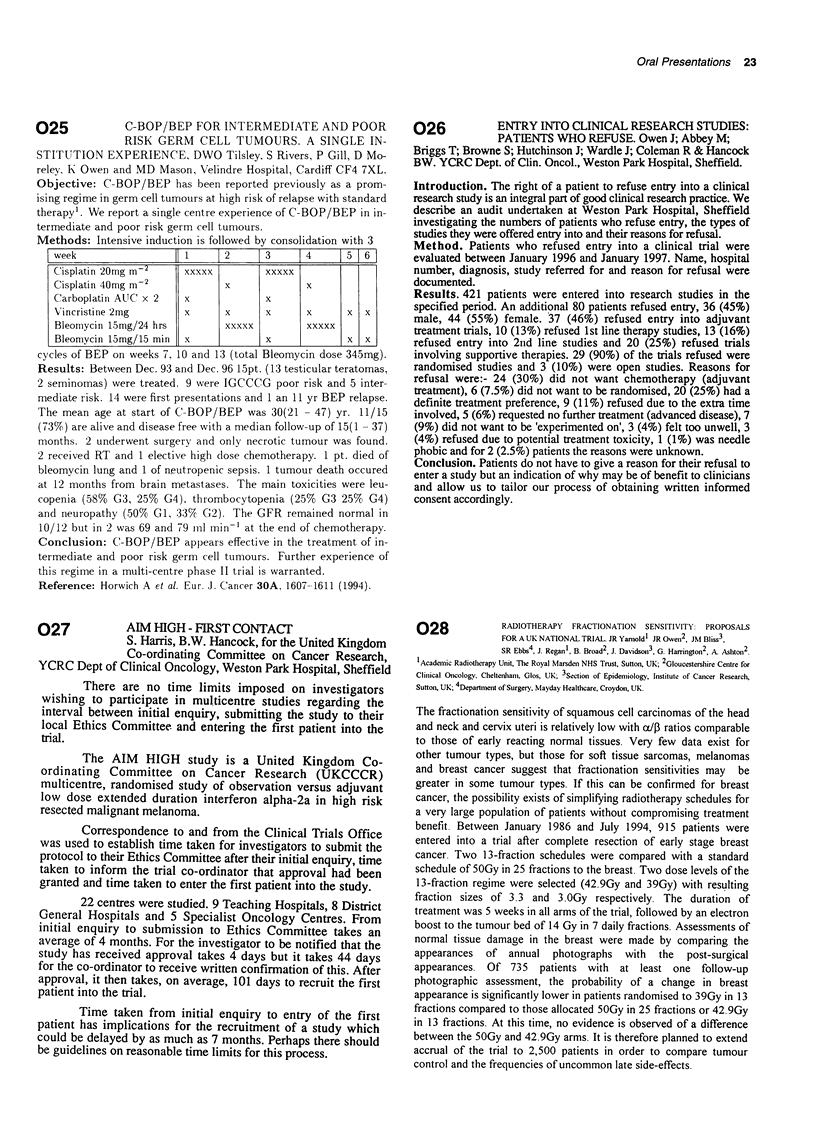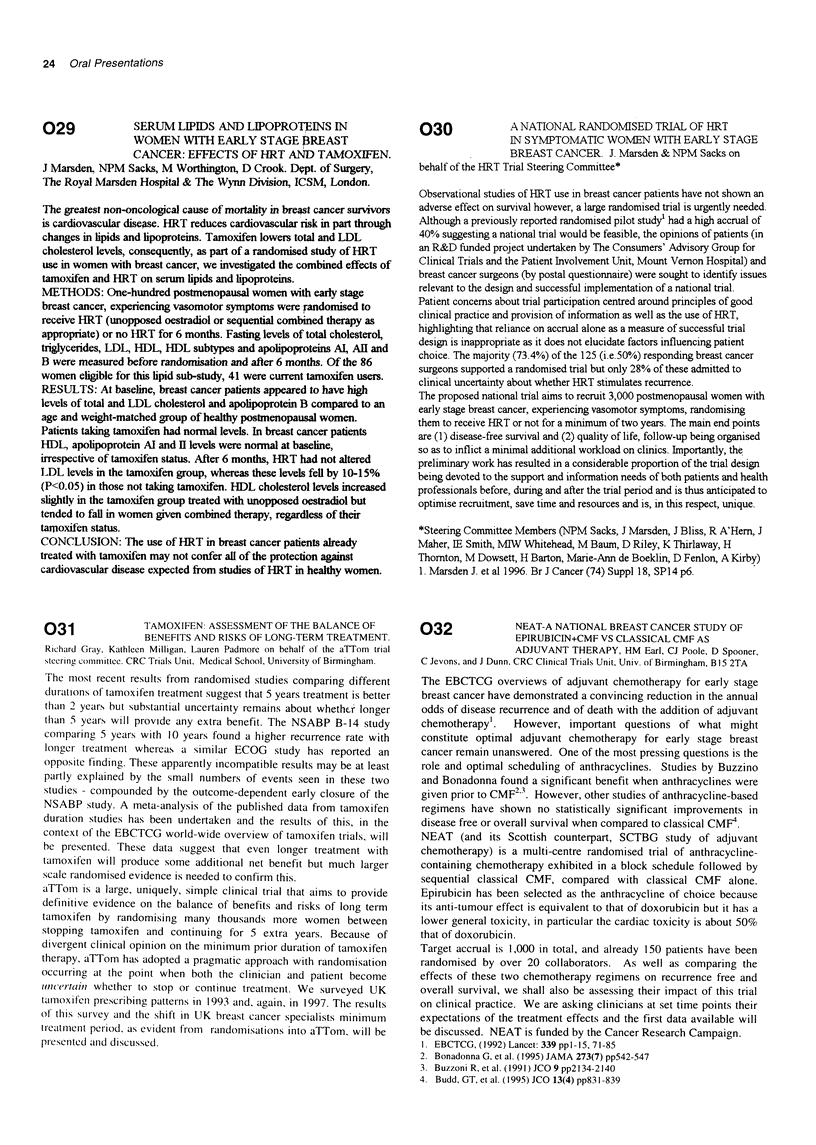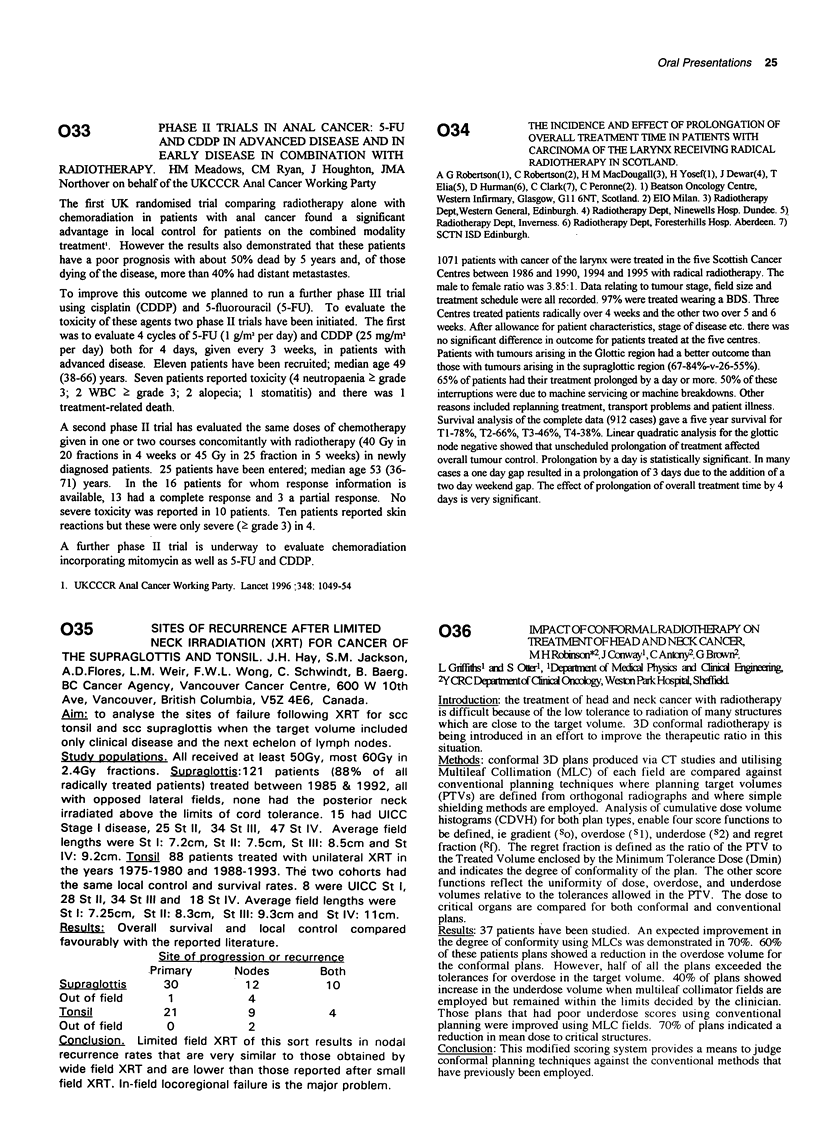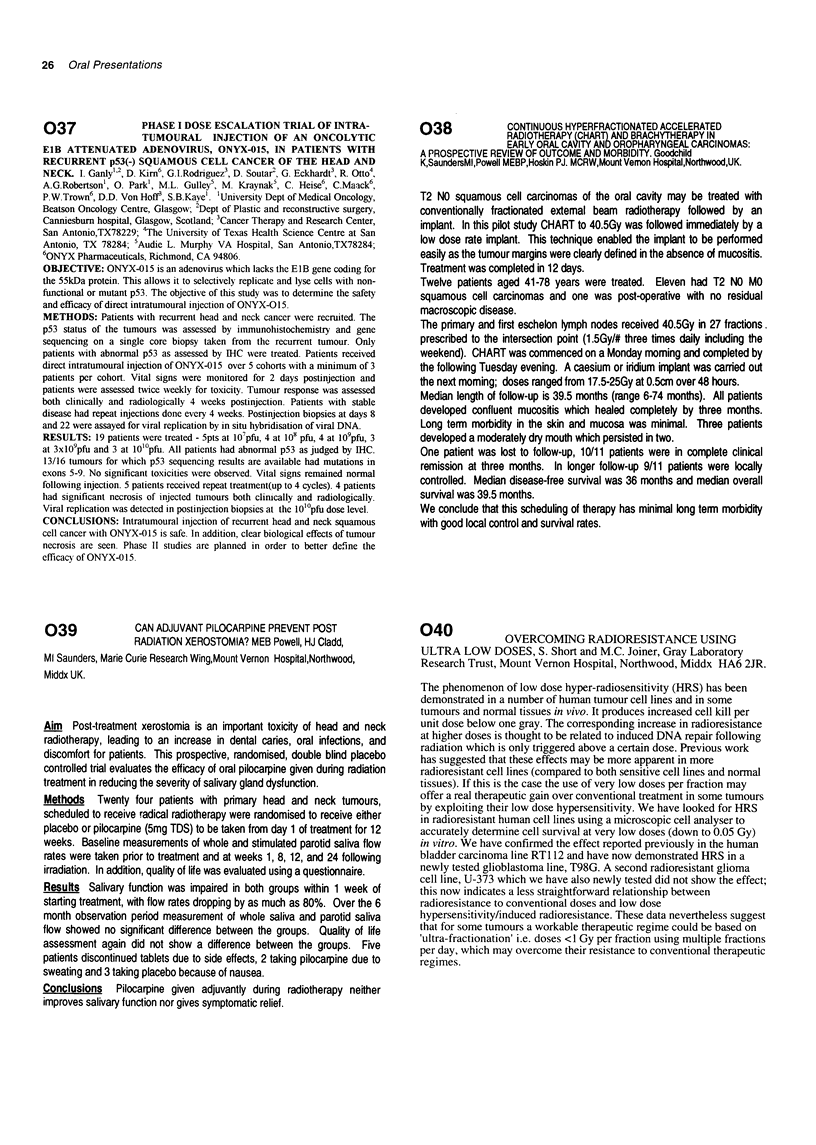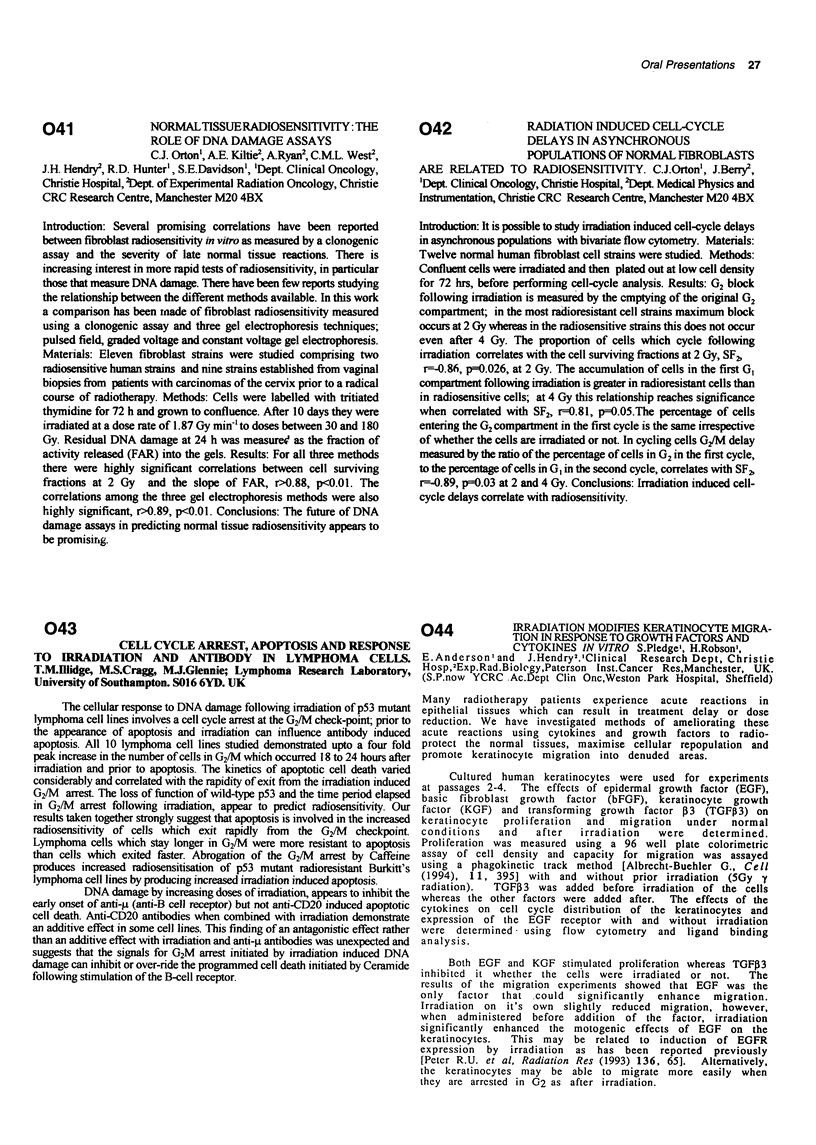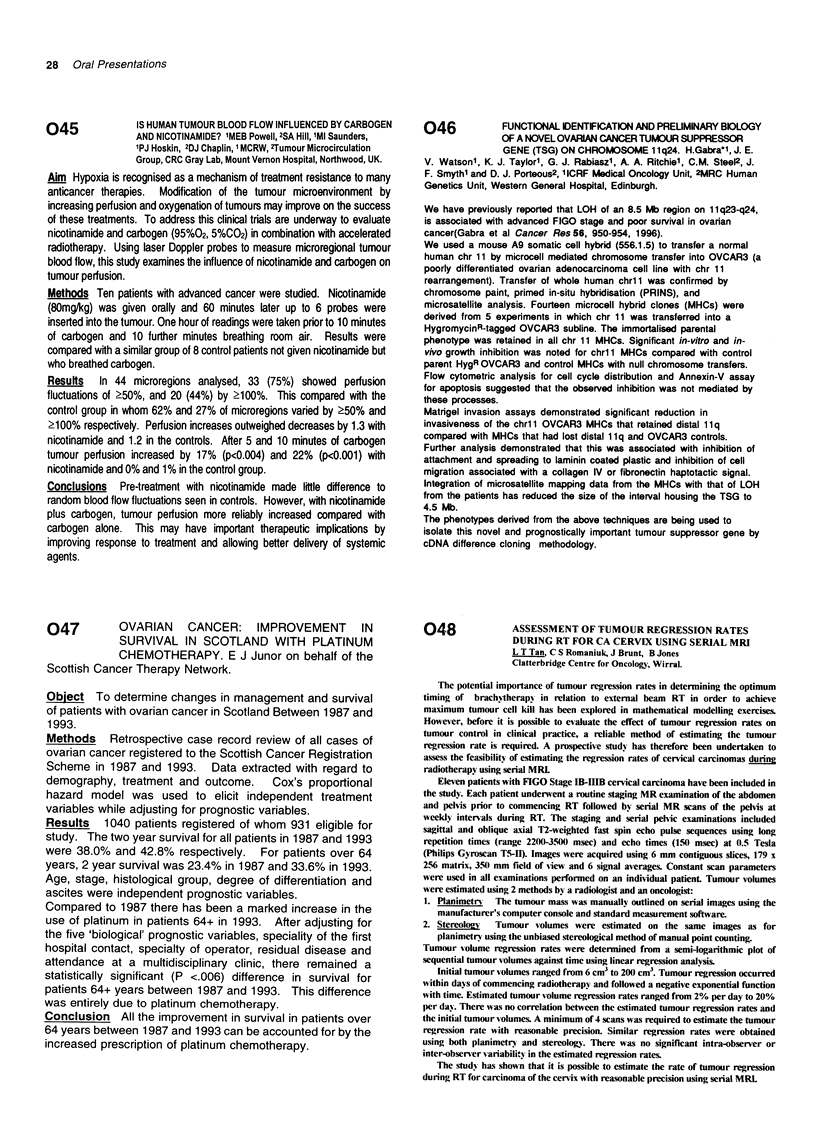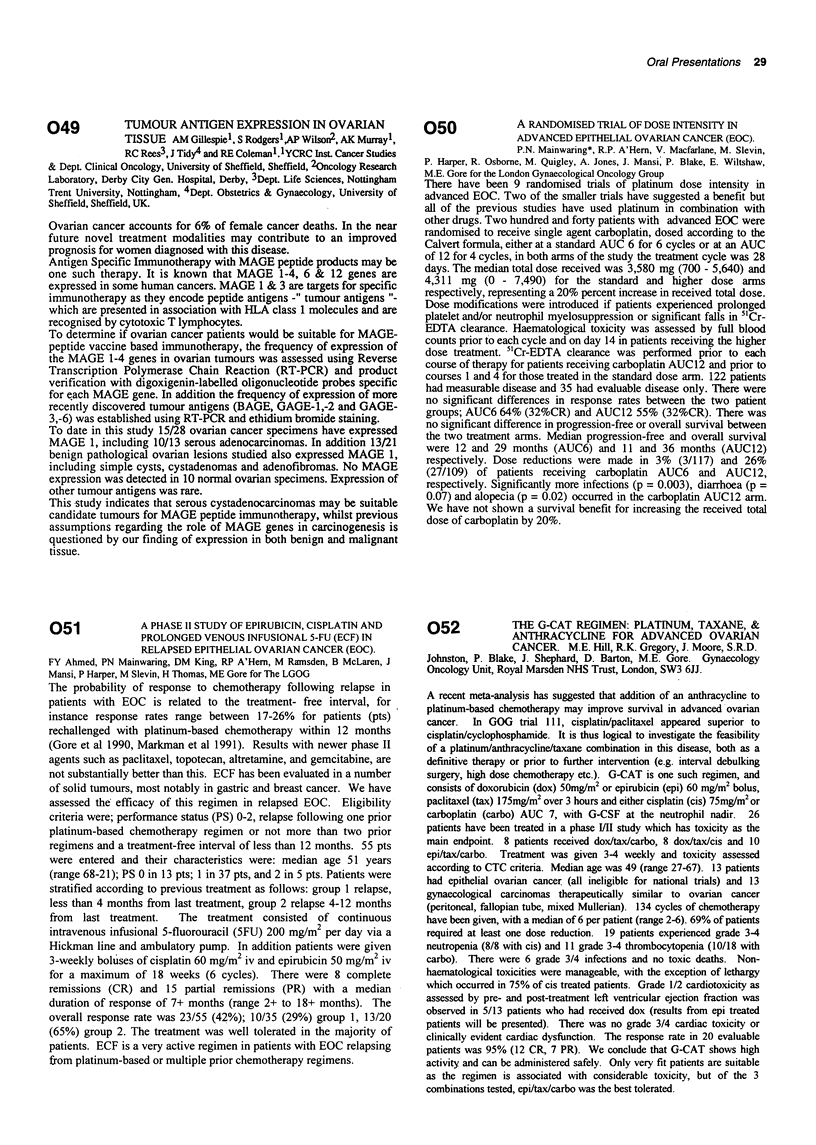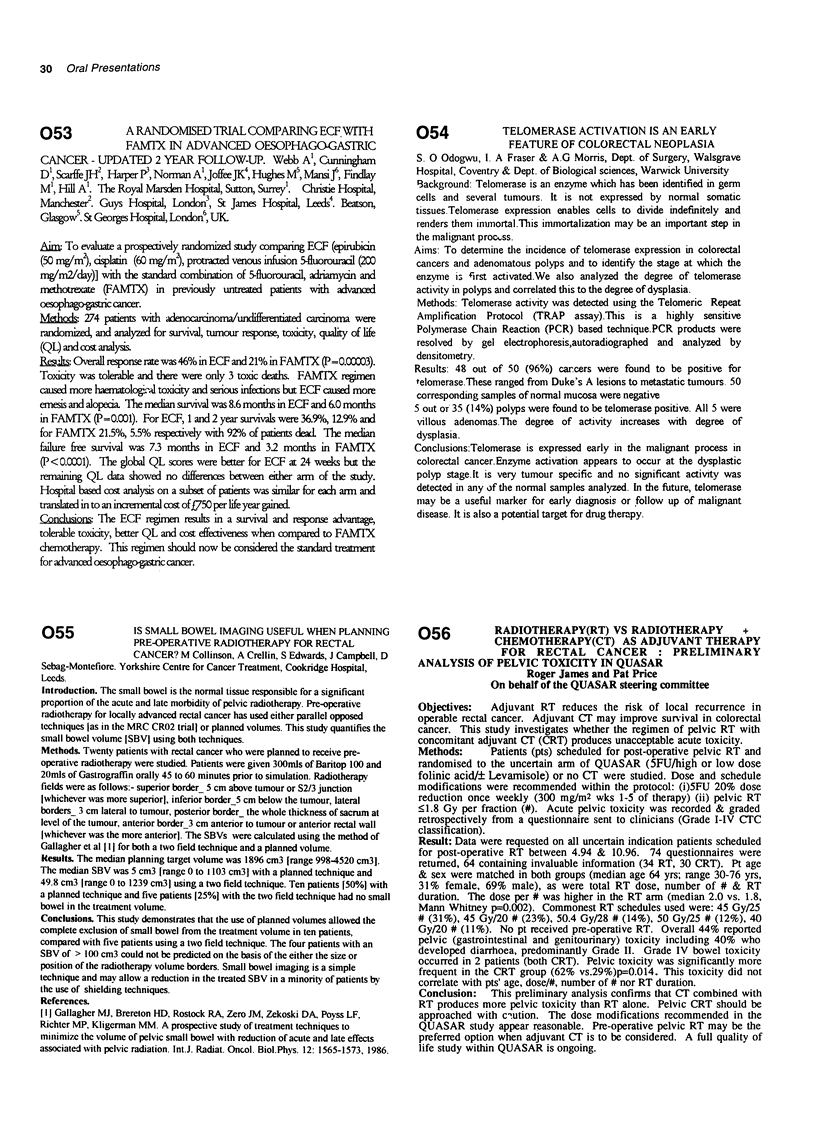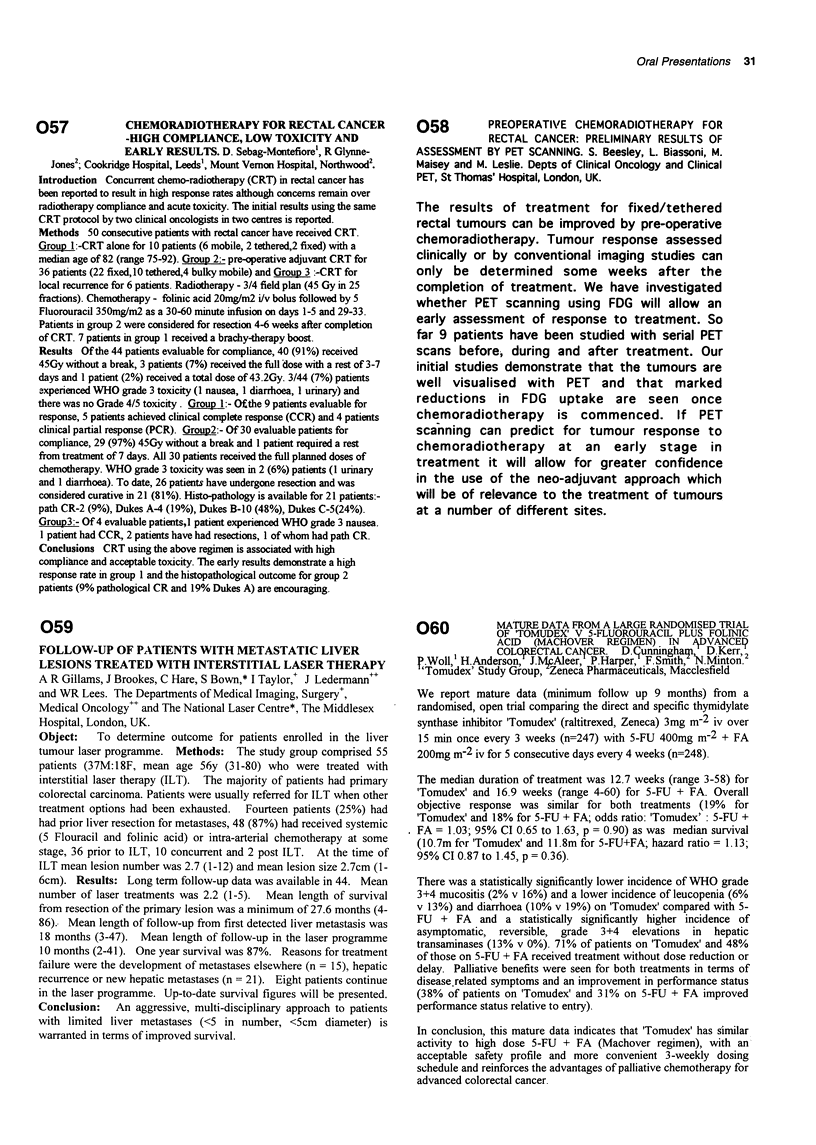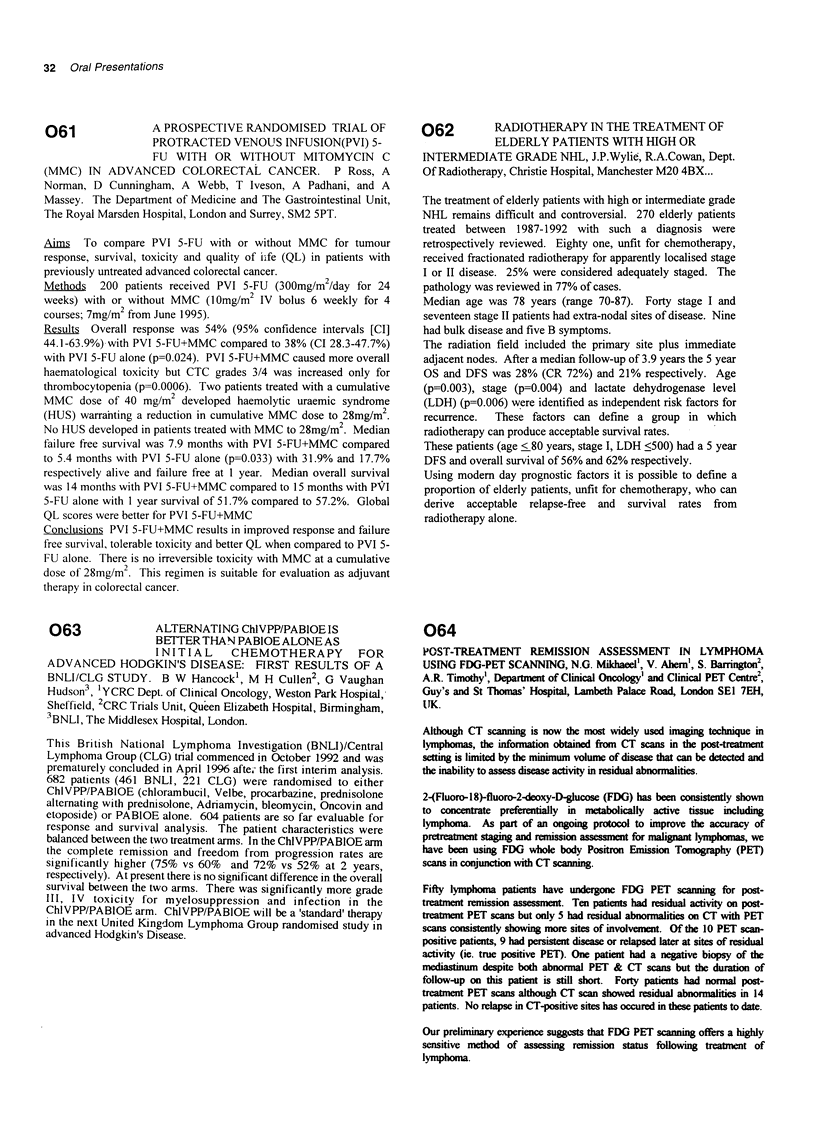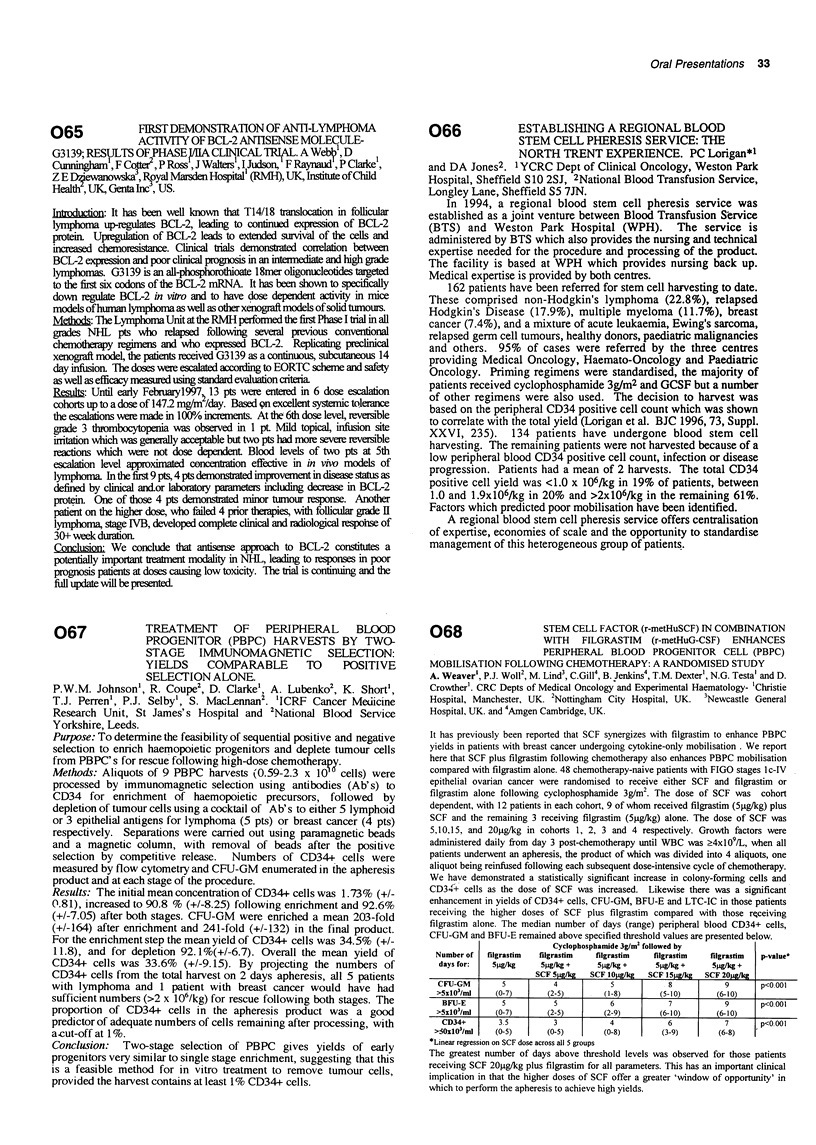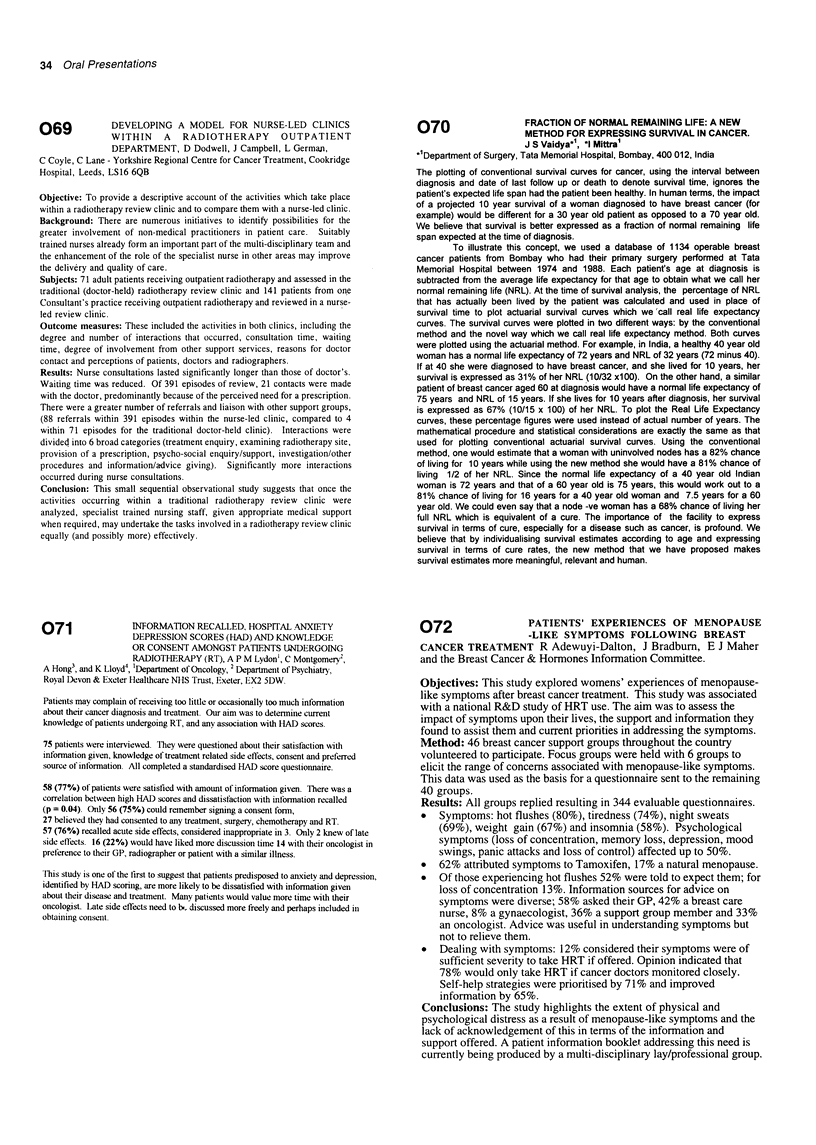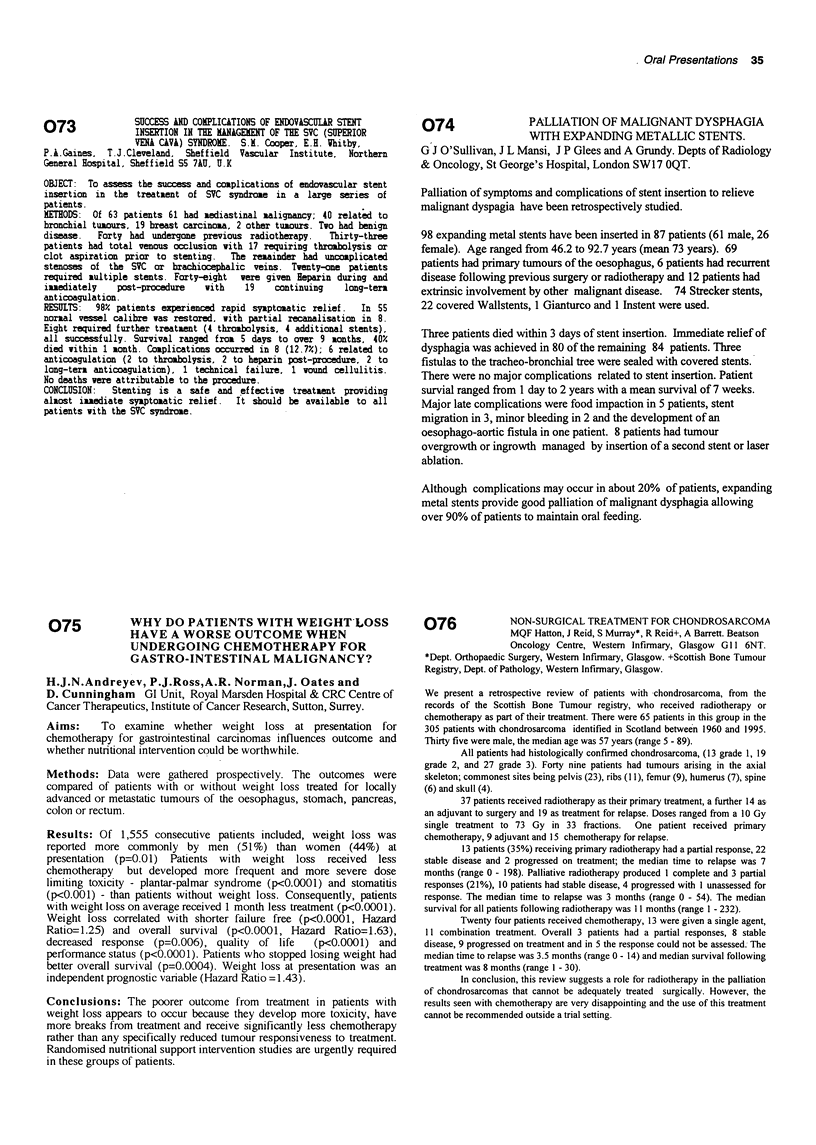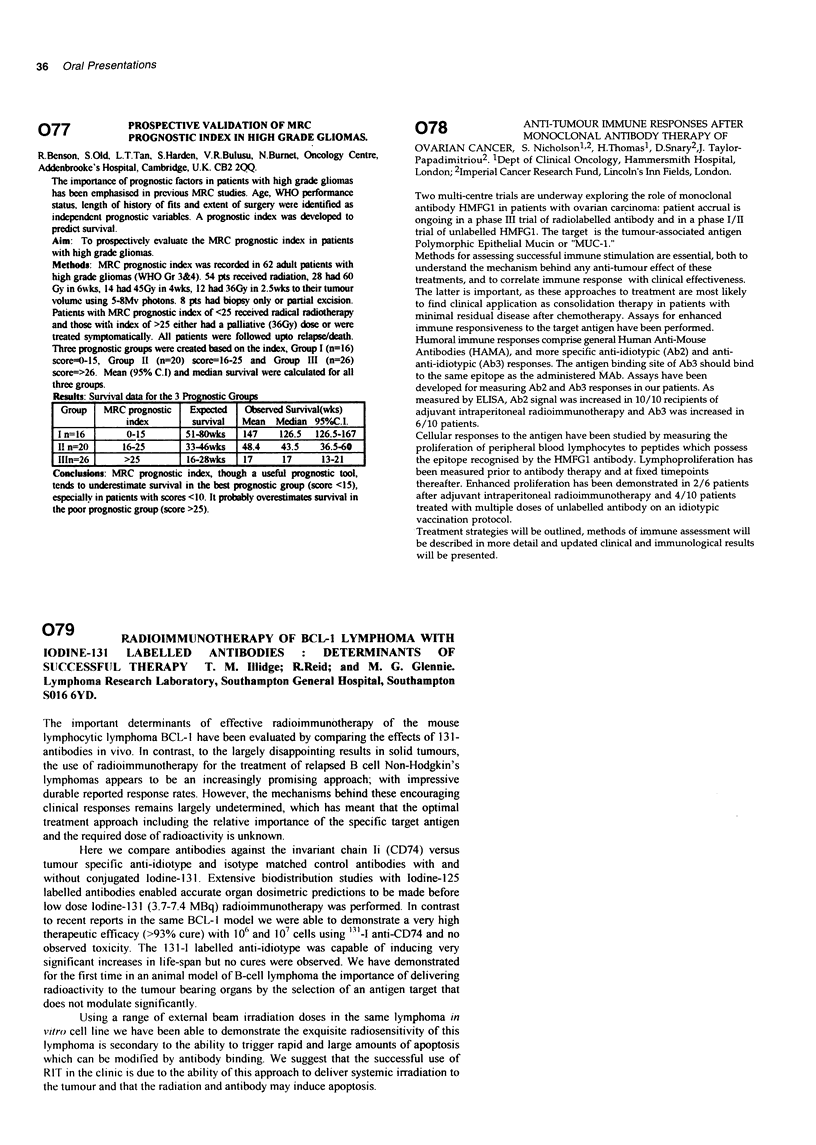# Oral presentations

**Published:** 1997

**Authors:** 


*British Journal of Cancer* (1997) **76**(Suppl 1), 15–36
© 1997 Cancer Research Campaign

### Oral presentations

### O01

CAN THE PRESENCE OF MICROMETASTASES (MM) IN PRIMARY BREAST CANCER (PBC) PREDICT OUTCOME? LONG TERM (MEDIAN 12.5YRS) FOLLOW UP.
H Gogas[1], JL Mansi[1], J Bliss[2], and RC Coombes[3]. [1]Dept Oncology, St George's Hosp, London SW17 0QT, [2]Institute of Cancer Research, Surrey, SM2 5NG, [3]Charing Cross Hospital, W6 8RF

Bone marrow (BM) MM were first identified immunocytochemically in patients with PBC in 1981. We report the longterm follow up of 350 PBC patients (median age 59 years; range 26 to 85 years) who had multiple aspirates taken between 1981 and 1986 at the time of initial surgery . The BM was examined with anti-EMA. MM were found in 89 (25%) patients. Their presence was related to various poor prognostic factors: pathological size (PS), peritumoral vascular invasion (VI) and +ve lymph nodes (LN). At a median follow up of 12.5 years 151 patients have metastatic disease, the 10 year relapse-free survival is 43.9% (CI 33.4-54.7%) in patients with MM and 62.7% (CI 56.5-68.6%) in patients without MM at presentation (logrank test 12.23; df = 1; $p < 0.001$). Cox regression analysis for relapse-free survival after allowing for PS, LN and VI shows that the effect of MM is reduced and no longer significant. 179 patients have died, 136 from breast cancer. The 10 year survival is 44.9% (CI 34.2-55.9%) for patients with MM and 65.7% (CI 59.4-71.5%) without (logrank test 13.90; df = 1; $p < 0.001$). Again Cox regression analysis for overall survival, after allowing for other prognostic factors, shows that MM as an independent factor is reduced and no longer significant. Therefore even though the shorter disease-free and overall survival occurs in patients with MM it is not an independent prognostic factor. Thus this technique may be of more value where PS, VI and LN status are unavailable such as in patients receiving primary medical therapy.

### O02

A COMPARISON OF ISOCENTRIC AND CONVENTIONAL PLANNING IN INTACT BREAST RADIOTHERAPY.

S.Pledge[1],C.Anthony[1],G.Brown[1], S.Young[2],J.Conway,[2]M.Robinson[1],[1]YCRC Acad.Dept.Clin.Oncology [2]Physics Dept., Weston Park Hospital, Whitham Road, Sheffield UK.

**Introduction** The accuracy of breast radiotherapy has now become an important clinical and legal issue. To improve accuracy we have recently developed an isocentric technique which requires simulator time not needed with our conventional technique.

**Aim** To compare the volume of lung irradiated, the day to day accuracy and the costs of each technique.

**Patients** 57 isocentric plans (IP) and 40 non isocentric plans (NIP) patients were studied. A subset of 32 of each had the Central Lung Distance (CLD) determined

**Methods** Portal images of treatment fields have been acquired using Portal Vision or film, mean 9.8 images per IP and 8.3 for the NIP. The CLD was determined as the distance from the posterior field edge to the anterior chest wall on the beam axis. A more detailed analysis was also employed, using the Portal Image Processing System 2 (PIPS2, Manitoba Cancer Treatment and Research Centre) This computerises the registration of the reference and treatment portal images. The breast outline is marked on a digital image, and registration performed using a least square fit of the curves. Any resultant translation of the field centre is recorded as X and Y values with an assessment of axial beam rotation.

**Results** The mean CLD on the first portal image for the NIP was 21.9 mm (1SD 5.6), and on the simulated CLD for the IP was 18.4 mm (1SD 9.7). The numbers of images where the CLD exceeded 30 mm was 3 for the IP and 10 for the NIP ($\chi^2$ 5.11, p 0.02). There was no significant difference in variability of rotation or longitudinal accuracy between the techniques. The mean variation in lung radiation was significantly greater in the NIP (2.33mm (95%CI 1.94-2.72) vs 0.92mm (95% CI 0.42-1.42)

**Conclusions** Use of the isocentric technique reduces lung irradiation and set up inaccuracy whilst inter fraction variation is unchanged. This is achieved at the cost of increased radiographer and Physics Department input.

### O03

**LOCAL RECURRENCE OF BREAST CANCER: NEW CONCEPTS ABOUT ITS BIOLOGY AND CLINICAL RELEVANCE.**
**J S Vaidya*[1], I Mittra[2], M Baum[1],** [1]Departments of Surgery at University College London, 67-73 Riding House Street, London, W1P 7LD,UK, and at [2]Tata Memorial Hospital, Bombay, 400 012, India.

It is believed that breast cancer is multicentric and harbours microscopic foci of cancer apart from the presenting tumour. A detailed pathological analysis by Holland et al (Cancer,1985;56:979-90) suggested that although multicentric foci (MCF) are present all over the breast, 90% of them are found in the index quadrant. In randomised clinical trials of breast conservative therapy it was found that radiotherapy effectively reduced the incidence of local recurrence. Additionaly, in these trials, >90% of early breast recurrences were found to occur in the index quadrant and therefore it was believed that they arise from MCF and that all MCF correctly deserved treatment with whole breast radiotherapy.

However, results of our recent analysis of spatial distribution of MCF within the breast suggest a different picture. We used the relative positions within the breast of the primary tumour and MCF were used to calculate the relative distribution of primary tumour and MCF in the 4 quadrants of the breast and the percent breast volume that would be required to be excised to include all MCF. The relative distribution of primary tumour and MCF in the 4 breast quadrants was significantly different (p=0.034). MCF were present beyond the index quadrant (25% of breast volume including the tumour) in as many as 79% (15/19) of breasts that harboured MCF; and, in half the cases (15/30) when all breasts were considered. Thus, in half of all patients, MCF are present in other quadrants. If MCF were giving rise to the early breast recurrence half would have occurred in other quadrants; but 90% of early breast recurrence occurs in the index quadrant, whether or not radiotherapy is given. We also know that local recurrence occurs in the index quadrant irrespective of clear margins, which suggests that it does not arise from overlooked tumour. Interestingly, a recent paper (Deng G et al, Science,1996;274:2057-59) has demonstrated a loss of heterozygocity in morphologically normal tissue at the excision margins of breast cancer.

We propose that local recurrence arises a) from circulating metastatic cancer cells lodging in the highly vascular surgical bed (local relapse does harbinger a poorer prognosis) or b) from local trasfection of surrounding breast epithelium by nuclear material released from the original malignant clone resulting in insertional mutagenesis. Thus although the margins of excision are morphologically clear, they may be genetically unstable.

In any case, if MCF do not give rise to breast recurrence then why should we treat them with either mastectomy or whole breast radiotherapy? It is as necessary or as unnecessary as treating the contralateral breast! We have begun pilot studies of a clinical trial to test whether radiotherapy to the index quadrant alone can achieve good local control.

### O04

ROLE OF RADIOTHERAPY IN EARLY BREAST CANCER (STAGE I). A WEST MIDLANDS BREAST GROUP PROSPECTIVE RANDOMISED COLLABORATIVE STUDY (BR3002).
D.Spooner,J.M.Morrison,G.D.Oates,D.J.Ellis,M.J.R.Lee, A.Aukland,R.J.Grieve,R.J.Blunt,H.M.Bishop,J.A.Dunn,C.Jevons, K.Milligan,L.Dodson. CRC Trials Unit on behalf of the West Midlands Breast Group, Queen Elizabeth Hospital, Birmingham, U.K, B15 2TA

Between August 1985 and December 1992, 707 patients with early breast cancer (less than 4cm diameter with clinically negative axillae) were treated with wide local excision only and then randomised to receive immediate post-operative radiotherapy to the breast and axilla or observation only. All patients received adjuvant Tamoxifen for a minimum of two years.

After a median follow up of 7 years, 530 patients are still alive and 37 have died from conditions unrelated to carcinoma of the breast. The overall two year actuarial survival is 93%. There is no significant difference between those receiving radiotherapy (93%) and those who were subsequently observed (92%) ($\chi^2$=1.81, P=0.18).

There is a difference in the two year actuarial relapse rate: 8% in those who received radiotherapy and 18% in those who did not (overall 13%). This is statistically significant ($\chi^2$=24.1, P<0.0001). There was no difference in the two year distant metastatic relapse rate: 11/343 in the no-radiotherapy group, 16/348 in those who received radiotherapy ($\chi^2$=0.89, P=0.34). However, there was a very significant difference in locoregional relapse at two years: 51/343 in those who did not receive radiotherapy, compared with 16/348 in those who did ($\chi^2$=20.8, P<0.0001).

This trial was supported by the Cancer Research Campaign.

### O05

**LOCAL IRRADIATION REDUCES BREAST RELAPSES IN PRESENCE OF SYSTEMIC ADJUVANT THERAPY.** I Potyka, M Fennessy, J Houghton, M Baum, W Odling-Smee on behalf of the CRC Breast Cancer Trials Group

In view of the increased use both of local excision as initial surgery and of adjuvant systemic therapy a multi-centre randomised controlled trial was designed to evaluate whether women with early operable breast cancer, for whom adjuvant systemic therapy was planned, required radiotherapy to prevent local relapse. Following primary surgery patients were randomised, with consent, to either the radiotherapy arm or to the control arm, using uncertainty as the criteria. Radiotherapy was given according to local protocols. Depending on age, patients were also entered into the CRC Adjuvant Breast Trial for Patients under the Age of Fifty (a 2x2 factorial design evaluating Zoladex and tamoxifen for 2 years) or into the CRC Adjuvant Breast Trial for Patients over the Age of Fifty (in which patients were randomised after completing two years of tamoxifen to continue for a further three years or to stop).

Between the launch in 1986 and 1995 when the trial was closed 585 patients were randomised by 35 clinicians in the UK. This analysis included 535 eligible patients who had been followed for at least one year: 265 patients received radiotherapy and 270 were randomised into the watch policy group.

At a median follow-up of 3.4 years (range 0.2-8.4 years; live patients) a significant reduction in local recurrence in the radiotherapy arm was demonstrated (RR = 0.36; 95% CI 0.23 - 0.57). There was no difference in overall survival between the two groups (RR = 1.2; 95% CI 0.8 - 1.8: logrank analysis). The number of uncontrolled local recurrences at death was the same in the two arms.

For patients receiving systemic adjuvant therapy for early breast cancer the reduction in risk of local relapse from 36% at five years to 14% by radiotherapy may justify its use.

### O06

**THE EFFECT OF NEOADJUVANT CHEMOENDOCRINE THERAPY ON ANGIOGENESIS IN PRIMARY BREAST CARCINOMAS.** Makris A[1], Powles TJ[1], Kakolyris S[2], Ashley SE[1], Dowsett M[1], Harris AL[2]. [1]The Royal Marsden Hospital, Sutton and London; [2]ICRF Clinical Oncology Unit, Oxford, UK.

Survival in breast cancer patients is strongly predicted by the intensity of angiogenesis, as measured by microvessel density (1). The impact of chemotherapy and/or endocrine therapy on angiogenesis is unknown. Histological samples were obtained from patients randomised to a trial of neoadjuvant (NEO) vs adjuvant (ADJ) chemoendocrine therapy in operable breast cancer. Patients received 4 cycles of chemoendocrine therapy prior to surgery in the NEO group. Chemoendocrine therapy consisted of mitoxantrone 11mg/m$^2$ and methotrexate 35mg/m$^2$ combined with tamoxifen 20mg/day (2MT). The two groups were comparable for age, menopausal status, stage and surgical requirements. From a random sample of 216 patients (105 NEO and 111 ADJ) entered into the trial, invasive cancer was identified in 195 patients (91 NEO and 104 ADJ). Using these samples immunostaining was performed with the CD34 monoclonal antibody and scoring of microvessels done using the Chalkley method. The median score of NEO patients was 5.7 (95% CI 5.3 to 6.0) and for ADJ patients was 6.3 (95% CI 6 to 6.7), p=0.025. Using previously validated scoring categories (1) there were more good prognosis (score <5) samples in the NEO group (30%) than in the ADJ group (19%) and correspondingly less poor prognosis (score ≥7) samples in the NEO group (23%) than in the ADJ group (34%) (p=0.04). These results suggest that neoadjuvant chemoendocrine therapy causes a reduction in microvessel density in primary breast carcinomas. This effect could be either due to a direct effect on angiogenesis or secondary to tumor regression.

1. Fox SB et al. Quantitation and prognostic value of breast cancer angiogenesis: comparison of microvessel density, Chalkey count, and computer image analysis. J Path 1995; 177: 275-283.

### O07

**MITOZANTRONE AND METHOTREXATE CHEMOTHERAPY WITH AND WITHOUT MITOMYCIN C IN THE REGIONAL TREATMENT OF LOCALLY ADVANCED BREAST CANCER.** J.C.Doughty, E. Kane, T.G. Cooke, *C.S. McArdle. University Departments of Surgery, Glasgow Royal Infirmary and *Edinburgh Western General Infirmary, UK

Regional chemotherapy is one method of treating patients with locally advanced breast cancer. Conventionally it is delivered by surgical implantation of a catheter into the subclavian artery but the less invasive angiographic technique is increasingly being used.

There have been many reports of mitozantrone, methotrexate and mitomycin (MMM) administered regionally but we have experienced severe wound breakdown associated with the use of mitomycin C (MMC).

This paper therefore compares the results and toxicity obtained delivering mitozantrone and methotrexate (MM) with and without MMC via angiographically placed catheters in the internal mammary and lateral thoracic arteries.

Fifty-two patients with a histological diagnosis of locally advanced breast cancer were recruited. Thirty-three patients received MMM and 17 MM. The response rates as defined by WHO criteria were as follows:

| | MMM | MM |
|---|---|---|
| Number of patients | 33 | 17 |
| Complete response | 11 | 5 |
| Partial response | 13 | 8 |
| Stable disease | 8 | 4 |
| Progressive disease | 1 | 0 |

There was no significant differences ($x^2$ test p>0.7) between the responders and non-responders in the two group, however local toxicity was significantly increased in the patients who received MMM.

We conclude that regional chemotherapy using MM is as effective as MMM in the treatment of locally advanced breast cancer and avoids the severe local skin reactions associated with MMM.

### O08

**A RANDOMISED PHASE II TRIAL OF INFUSIONAL 5-FU AND EPIRUBICIN WITH CYCLOPHOSPHAMIDE V. CISPLATIN IN ADVANCED BREAST CANCER.** T.Eisen, I.E.Smith, P.A.Ellis, M.O'Brien, J.Prendiville, A.Saini and S.Johnston. Royal Marsden Hospital, London SW3 6JJ.

Object of Study We previously developed an in-patient regimen consisting of infusional 5-FU, epirubicin and cisplatin (ECisF) and a response rate of 86% in metastatic/locally advanced breast cancer. This phase II 2:1 randomised study investigates whether cyclophosphamide (ECycloF) can be substituted for cisplatin to reduce toxicity and allow the regimen to be administered on an out-patient basis without loss of efficacy.

Methods 76 women (30-73y, median 48y) with breast cancer (34 locally advanced, 42 metastatic) received continuous infusional 5-FU (200mg/m$^2$/day via Hickman line) and 6 cycles of epirubicin (60mg/m$^2$ q21days) with either cyclophosphamide 600mg/m$^2$ q21days (24 locally advanced, 29 metastatic) or cisplatin 60mg/m$^2$ q21days (10 locally advanced, 13 metastatic). There were no significant differences in patient characteristics between these groups.

Results There were no significant differences in toxicity between ECycloF and ECisF; including WHO Grade 3/4 nausea and vomiting (4% v 13%), gastrointestinal toxicity (11% v 13%), anaemia (0% v 4%), thrombocytopenia (2% v 4%) or leukopenia (22% v 14%). Efficacy was comparable with overall response 68% v 65%, CR 15% v 17% and median progression-free survival 9 v 8 months.

Conclusion ECycloF is an out-patient regimen with no increased toxicity over ECisF and with comparable efficacy.

### O09 SHOULD IMMEDIATE BREAST RECONSTRUCTION BE AVAILABLE TO ALL WOMEN UNDERGOING MASTECTOMY?

**R.A.M. Al- Mufti , MG Berry, S Denton, M Sullivan, A Vaus, R Carpenter .**
**The Breast Unit, Royal Hospital's Trust at St. Bartholomew's Hospital, London EC1A 7BE**

In the united kingdom, immediate breast reconstruction following mastectomy for breast cancer has been available only to the few. The modern Breast Surgeon has acquired reconstructive skills such that more women can be offered immediate restoration of body image with primary reconstruction, but do the results justify such intervention?

We present our experience and clinical outcomes of the first 100 patients undergoing surgery in four years since the introduction of immediate breast reconstruction using the Becker tissue expansion prosthesis. As part of the evaluation a retrospective study was carried out by sending a detailed questionnaire to all patients for subject of assessment.

The median age was 52 years (range of women undergoing immediate reconstruction was 25-74), 49 had stage I, 38 stage II and 13 stage III disease. Thirty-nine women received radiotherapy and twenty-six adjuvant chemotherapy in addition to undergoing surgery. The clinical infection rate was 14%, however, organisms were grown in only 7%. Revision surgery for capsule contraction was undertaken in 28% with no statistical relationship to either radiotherapy or chemotherapy (p value > 0.5). Eight women have died of breast cancer and of the group remaining that are alive, 87% express satisfaction with the final aesthetic result.

We conclude that immediate breast reconstruction may be offered to more women undergoing mastectomy using the relatively simple technique and without affecting adjuvant treatments. A high degree of patient satisfaction seems not to have been affected by the relatively high revision surgery rate of 28%.

### O10 THYMIDINE SALVAGE DEMONSTRATED *IN VIVO* : A SPECIFIC PHARMACODYNAMIC ENDPOINT OF THYMIDYLATE SYNTHASE (TS) INHIBITION.

P.Wells*[1], R.N.Gunn[1], A.Hughes[2], G.A.Taylor[2],P.Price[1], D.R.Newell[2].

[1]. MRC CU, Hammersmith Hospital, London W12 0HS, [2]. CRU, Medical School, University of Newcastle, Newcastle NE2 4HH.

**Object of study:** THYMITAQ™ is a nonclassical TS inhibitor. We aimed to assess whether activity of thymidine salvage pathways could be demonstrated in tissues following treatment. This may then be used as a specific *in vivo* pharmacodynamic endpoint of TS inhibition.
**Methods:** Five patients in the Cancer Research Campaign (CRC) Phase I trial of THYMITAQ™ and three controls were studied. Paired [2-$^{11}$C]thymidine PET scans were performed, one before treatment and one on the second day of therapy, one hour after oral administration of THYMITAQ™ (250mg/m$^2$). PET tomographic data was acquired over 60 minutes with continuous arterial blood sampling measures allowing the calculation of a plasma thymidine input function (IF). Spectral analysis was used to deconvolve the IF from the PET data to give a unit impulse response function (IRF) for each tissue region. Measurements of tracer delivery ($IRF_{1min}$), and retention ($IRF_{1hour}$)were obtained. The %difference in $IRF_{1hour}$ between each pair of scans is used as an expression of change of thymidine retention.
**Results:** No change was seen in extraction of tracer from the blood and delivery to the tissues as a result of the drug administration. There was a statistically significant increase in the retention of labelled thymidine at the end of the study relative to controls in the tumour regions (THYMITAQ™ mean +40% range +10% - +75%, controls mean -11% range +4% - -24% p<0.01 paired ttest). No change in the normal tissue (liver) was found.
**Conclusion:**Thymidine kinase and other salvage enzymes are active following TS inhibition to restore intracellular levels of thymidine nucleotides. The increase in tissue [2- $^{11}$C]thymidine concentration after administration of THYMITAQ™ is consistent with enhancement of the thymidine salvage pathway in tumour relative to normal tissue.

Supported by CRC GrantNoSP2193/0102 & Agouron pharmaceuticals.

### O11 EFFECTIVE TARGETING OF COLORECTAL CANCER *KIRSTEN RAS* POINT MUTATIONS WITH ANTISENSE THERAPY *IN-VITRO.*

**P.J. Ross, H.J.N. Andreyev, D. Cunningham, P.A. Clarke**
**GI Unit, Royal Marsden Hospital & CRC Centre of Cancer Therapeutics, Institute of Cancer Research, Sutton, Surrey**

**Introduction:** Codon 12 mutation of the *Kirsten ras (Ki-ras)* gene are present in about 30% of patients with colorectal cancer. Their presence is associated with worse outcome but they do offer an unique target for molecular therapies such as antisense. It is known that replacement of mutated with wildtype *Ki-ras* in cell culture, reduces the cell's malignant behaviour. This study aimed to reverse the effect of a glycine to valine *Ki-ras* codon 12 mutation with antisense oligonucleotide therapy. Of all possible *Ki-ras* mutations, this one carries the worst prognosis.
**Method:** Seven different, 17 nucleotide, phosphodiester backbone, phosphorothioate capped antisense molecules were examined. A cell free system was used to test each oligonucleotide's efficacy at inducing RNase H cleavage of the mutated mRNA. Wild type and codon 13 mutated mRNA were used as controls. These same oligonucleotides were also screened against 3 colorectal carcinoma cell lines in culture: SW480 with the target mutation, HT29 with wildtype *Ki-ras* and LoVo with a codon 13 mutation. Cells were permeabilised with streptolysin O and treated with 10 µM of each oligonucleotide. Thymidine incorporation into DNA measured the effect of a single dose of each design on cellular DNA synthesis. Protein expression was examined by Western blotting. Each oligonucleotide was tested at least 12 times against each cell line. Every experiment included permeabilised control cells which were not treated with the antisense oligonucleotide.
**Results:** Reduction in thymidine incorporation after a single dose of oligonucleotide varied from 5-32% in treated SW480 target cells but was minimal or not seen in the 2 control cell lines. Cleavage of mRNA seen in the cell free system correlated with results obtained with cells in culture. In some cases, antisense molecules could cleave both target and control mRNA. However, the most effective molecule in the cell line assay specifically cleaved the target mRNA but not control mRNA.
**Conclusions:** Antisense oligonucleotides targeting point mutations in *Ki-ras* mRNA have selective activity. This has been demonstrated in a cell free system and in cell culture. Efficacy of individual oligonucleotide designs cannot be predicted empirically, but the most effective inhibition seen in cell culture appears to occur because of selective RNase H cleavage.

### O12 IDENTIFICATION OF CIRCULATING TUMOUR CELLS BY RT-PCR IN PATIENTS WITH COLORECTAL CANCER; DETECTION PRIOR TO EVIDENCE OF METASTASIS.

SK Jonas, A Klokouzas, RQ Wharton & TG Allen-Mersh, Dept. of Surgery Chelsea & Westminster Hospital, CXWMS, London SW10 9NH

Carcinoembryonic antigen (CEA), a member of the immunoglobulin superfamily is an epithelial specific antigen. Identification of CEA RNA in the circulation therefore precludes the transcription of CEA by haemopoietic cells.

We have previously identified CEA cDNA by RT-PCR in patients with colorectal liver metastasis (LM). Sensitive detection was achieved by Southern blot hybridisation using an oligoprobe which lies between the CEA specific primers used and identity of PCR products was confirmed by sequence analysis. Extracted RNA from granulocytes which express non-specific cross-reacting antigen (NCA), a closely related member of the CEA family and with substantial sequence homology, was amplified by NCA-specific primers but not by CEA primers, eliminating the possibility of granulocyte contamination. Our studies now demonstrate that the prevalence of CEA RNA expression in blood sampled preoperatively is similar in patients with primary (PR) colorectal cancer without evidence of metastasis as confirmed by CT-scan (PR, no LM 11/20, 55%; PR with LM 36/54, 67%). The application of a cannula for venous sampling to prevent skin cell contamination established that 23 non cancer control patients were negative on Southern blot hybridisation.

Multiple sampling in which 2 samples were taken within one circulation time and a further sample outside this time resulted in increased frequency of detection. CEA cDNA amplification occurred in 61% of colorectal cancer patients from only a single sample of blood, but increased to 94% overall positive expression (15/16 patients, 7 PR with and 8 without LM) when 3 separate samples were taken. This suggests that multiple tumour cell clusters are present in the circulation of all these patients.

The identification of circulating CEA producing cells in patients without evidence of metastasis may be of clinical importance.

### O13 INVESTIGATION OF THE ANTITUMOUR ACTION OF COMBRETASTATIN A4 AND ITS PHOSPHATE ANALOGUE.

K. Grosios, A.T. McGown[1], G.R. Pettit[2], M.C. Bibby, Clinical Oncology Unit, University of Bradford, Bradford BD7 1DP, UK. [1]Paterson Institute for Cancer Research, Manchester M20 9BA, UK. [2]Arizona State University, Tempe, Arizona 85287-1604, USA

Currently one of the main interests in cancer research is the identification of clinically useful targets. Tumour vasculature manipulation and interference with blood vessel formation (angiogenesis) are providing such targets and related drugs are increasingly considered for clinical trial, one of which is Combretastatin A4 (C-A4). In this study, the possible mechanism(s) of action of C-A4 and its phosphate analogue have been investigated in clinically relevant *in vivo* and *in vitro* models. Intraperitoneal administration of C-A4 (150 $mgkg^{-1}$) and the phosphate analogue (100 $mgkg^{-1}$) have resulted in complete vascular shutdown (as assessed by Hoechst 33342 studies) and extensive haemorrhagic necrosis in MAC15A subcutaneous and MAC15 orthotopic colon tumours in mice. Orthotopic transplantation results in formation of metastatic deposits. Small avascular deposits observed in the lungs of mice were unaffected by both compounds, whereas areas of focal necrosis were found in diaphragm, kidney and lymph node deposits, which do develop a vascular supply. Human Umbilical Vein Endothelial Cells (HUVEC) grown in type-I calf-skin collagen form tubular networks. Exposure of such networks to 0.31μM (non-cytotoxic) concentration of both C-A4 and its analogue results in disruption of such networks, which begins after 4h and is completed by 24h. Similarly, the same concentrations inhibit migration of HUVEC through collagen. Staining for F-actin and immunofluorescence for tubulin have shown complete disorganisation and disruption of the cellular cytoskeleton after 4h exposures. C-A4 and its phosphate analogue exhibit antitumour effects which may result from a combination of antivascular and antiangiogenic actions.

### O14 THE ENDOTHELIAL CYTOSKELETON AS A TARGET FOR CANCER THERAPY

GG Dark*, SA Hill, DJ Chaplin. Tumour Microcirculation Group, Gray Laboratory, Northwood, Middlesex HA6 2JR.

Many anticancer agents act on rapidly proliferating cells, without regard for the malignant phenotype and a major pharmacological goal remains the development of highly selective non-toxic therapies for the treatment of malignancies. Vasculature is critical to both the survival of a solid tumour mass and its continued growth. Indeed tumours cannot grow beyond 1-2 mm in diameter without developing a functional blood supply. The tubulin and actin cytoskeleton are critical for the endothelial process of; angiogenesis, cell division, motility, morphology, vascular permeability and the intracellular transport of macromolecules and receptors to the cell membrane.

Using a tumour microenvironment model system we have examined the cytotoxic effects of a number of cytoskeletal agents. The tubulin polymerisation and depolymerisation inhibitors show a preferential action against endothelium, compared to cancer cells. Podophyllotoxin, although a weak tubulin-binding agent, is more toxic to tumour cells than endothelium. Colchicine, Dolastatin, Taxotere, Vinblastine, Vincristine and Combretastatin have increased toxicity towards endothelium, with enhancement of this action under hypoxic (1% $O_2$) conditions, with further potentiation for Taxotere and Vincristine in coculture with tumour cells. Tumour cells show a reverse relationship with increased resistance to these agents under hypoxia. The cytotoxic action of these agents is greatest against proliferating endothelium, and this may explain the enhancement seen with coculture as the breast cancer cell lines studied produce VEGF in significant quantities. In contrast, endothelium that is quiescent, prior to and during drug exposure, is significantly more resistant to the actions of these agents, particularly combretastatin A-4 prodrug.

In view of these selective actions against endothelium, vascular studies were performed in experimental and human breast cancer models *in vivo*. Histology of a variety of human xenografts and murine transplants indicated extensive haemorrhagic necrosis with the cytoskeletal agents Colchicine, Vinblastine and Vincristine, although only when administered at the maximum tolerated dose. Taxotere does not share this effect. Following systemic administration of combretastatin A-4 prodrug at 100 $mg{\bullet}kg^{-1}$ (<10% MTD), extensive haemorrhagic necrosis was visualised at 6 hours in our breast cancer model system, with a selective and rapid reduction in functional vascular volume of 93%, persisting even after 18 hours.

These studies indicate that directing agents against the endothelial cytoskeleton, can elicit selective effects against proliferating endothelial cells *in vitro*, and induce rapid vascular shutdown within tumours *in vivo*, thus demonstrating the clinical potential of this approach.

### O15 INVESTIGATIONS OF A CYCLIC PEPTIDE ANTAGONIST OF THE αVβ3 INTEGRIN: IMPLICATIONS FOR TARGETED TUMOUR THERAPY,

R. Allman, W. G. Jiang*, P. Cowburn and M. Mason, Research Department, Section of Clinical Oncology, Velindre Hospital, Cardiff CF4 7XL, *Department of Surgery, University of Wales College of Medicine.

Results of systemic therapy for metastatic malignant melanoma, including cytokine based therapies, are disappointing. Rational drug design requires that a specific tumour-associated property is identified and an appropriate drug identified or designed to specifically target that property.

Upregulation of the αvβ3 integrin (vitronectin receptor) is associated with increasing metastatic potential in malignant melanoma.

We have investigated the activity of the cyclic pentapeptide cRGDfV (which is a potent inhibitor of αvβ3) on a panel of melanoma cells derived from different stages of tumour progression. All of the cell lines express high levels of αvβ3, with one of the cell lines also expressing low levels of αvβ5.

Our studies indicate that cRGDfV (i) inhibits αvβ3 mediated cell adhesion with a IC50 value of 0.2μM (ii) prevents colony formation in 3-dimensional collagen gels (iii) induces apoptosis of cells maintained in 3-dimensional collagen gels.

Preliminary investigations into the mechanism of the activity of cRGDfV indicates that peptide binding induces a rapid (< 5 minutes) dismantling of Focal Adhesion Complexes, phosphorylation of paxillin, and is associated with a calcium transient. The potency of cRGDfV is further illustrated by the ability of the peptide to induce apoptosis even in the presence of high levels of BCL2 and that exogenous peptide administered to cells already attached and growing on type I collagen - coated surfaces results in rapid rounding up and detachment of cells.

This observation indicates that the drug may not be relying upon contact inhibition for its effects. Administration of cRGDfV to normal skin derived fibroblasts, which express low levels of αvβ3 and high levels of αvβ5, caused no detectable change in morphology or viability of the cells.

### O16 A RANDOMISED PHASE 2 STUDY OF BB-10010 (MACROPHAGE INFLAMMATORY PROTEIN 1-α) IN PATIENTS WITH ADVANCED BREAST CANCER RECEIVING 5-FU, ADRIAMYCIN AND CYCLOPHOSPHAMIDE (FAC) CHEMOTHERAPY.

M.J. Clemons[1], E. Marshall[1], J. Dürig[1], K. Watanabe[1],D. Miles[2],H. Earl[3] ,BI Lord[1]J. Kiernan[1],K Towlson[2], .A. Griffiths[1],P. DeTakats[3], T.M. Dexter[1], N.G. Testa[1], A. Howell[1]. British Biotech plc, Oxford, U.K. [1]CRC Dept. of Medical Oncology and Paterson Institute for Cancer Research, Christie Hospital, Manchester. UK. M20 4BX.[2]ICRF Clinical Oncology Unit,Guy's Hospital, London,UK.SE1 9RT,[3]CRC Institute of Cancer Studies, Birmingham,UK.B15 2TT.

Whilst haematopoietic growth factors (HGFs) are established means of restoring neutrophil counts following cytotoxic chemotherapy, the bone marrow stem cells remain in cycle and they too are affected by the chemotherapy.The consequence of which is progressive bone marrow damage and thrombocytopenia. A novel approach would therefore be to keep stem cells out of cycle during periods of cytotoxic treatment to protect the quality of the normal stem cell population and thereby maintain the bone marrow reserve. BB-10010 is a variant of the human form of Macrophage Inflammatory Protein 1-α and has been shown in mice to both block entry of haematopoietic stem cells into S-phase and to increase the self-renewal capacity during recovery from cytotoxic damage. Between August 1995 and March 1996, 30 patients with locally advanced or metastatic breast cancer were entered into the first randomised, controlled phase 2 study designed to evaluate the potential myeloprotective effects of a seven day regimen of BB-10010 in patients receiving 6 cycles of FAC chemotherapy ( 5FU 600$mg/m^2$, Adriamycin 50 $mg/m^2$, Cyclophosphamide 600 $mg/m^2$, every 3 weeks). BB-10010 was administered as a sc: injection, once daily on days 0-6 of each treatment cycle, the chemotherapy was administered as a bolus on day 1. Patient characteristics; Median age 50 (29-64) years and median ECOG performance status of 1 (0-2). All patients have now completed the study. 10 patients were randomised to 100 μg/kg BB-10010 , 11 patients to 30 μg/kg BB-10010 and 9 control patients received no BB-10010. BB-10010 was well tolerated in all patients with no severe adverse events. 2 patients suffered repeated cutaneous reactions at the injection sites. Bone Marrow studies,to assess the LTBMC generation of GM-CFC, were carried out immediately preceding chemotherapy and at the end of 6 cycles in 20 patients. Neutrophils, platelets and circulating progenitor cell levels (GM-CFC and CD34+ cells) were determined at serial time points in cycles 1,3&6. Preliminary analysis of the results show similar neutrophil and platelet kinetics in all 3 groups.There was a non-significant trend in neutrophil recovery in the treated groups 21 days after cycle 6. Neutrophil counts ($x10^9$)were; control group (1.7,SD±0.52), 30 μg/kg BB-10010 (2.14,SD±0.52), 100 μg/kg BB-10010 (2.67,SD±0.52). FAC chemotherapy was associated with modest mobilisation of progenitor cells with an attenuated response over successive cycles of chemotherapy. The BM and peripheral blood progenitor level analyses will be completed in Feb. 1997 and the final results will be presented.

### O17 PHASE I STUDY OF THE BIODISTRIBUTION, PHARMACOKINETICS & IMMUNOGENICITY OF $^{111}$In-hCTMO1 (CDP671) ± PREDOSING WITH UNLABELLED hCTMO1 IN PATIENTS WITH LUNG CANCER.

G Middleton[1], M Verrill[1], K Priest[1], L Spencer[1], S Chittenden[2], R McCready[2], N Cox[3], L Skelton[3], M O'Brien[1], I Smith[1]. *[1]Lung Unit; [2]Physics Department, Royal Marsden Hospital, Downs Road, Sutton, Surrey, SM2 5PT, UK & [3]Celltech Therapeutics Ltd. 216 Bath Road, Slough, Berkshire, SL1 4EN, UK.*

$^{111}$In-hCTMO1 is an engineered human radiolabelled monoclonal antibody (Ab) to polymorphic epithelial mucin (PEM), a glycoprotein over-expressed on the surface of SCLC & NSCLC cells. This study was designed to confirm previous safety data and to study biodistribution of $^{111}$In-hCTMO1 in patients (pts) with lung cancer. In part 1, pts received an IV infusion of 1mg/kg $^{111}$In-hCTMO1. In part 2, following a dose of 1 mg/kg unlabelled Ab (to saturate free PEM binding and reduce liver uptake of labelled Ab) pts received 0.1 mg/kg $^{111}$In-hCTMO1. Toxicity was recorded using WHO criteria. Plasma levels of hCTMO1 were measured. Immunogenicity was studied by measurement of specific IgG and IgM titres.

Biodistribution was measured by whole body planar imaging. 21 pts received Ab, 8 after predosing. Median age 66 (44-76); M 18, F 3; SCLC 3, NSCLC 18. Infusions were well tolerated, 1 pt had a rigor after $^{111}$In-hCTMO1. Mean $t_{1/2}$ was 44 hours.

Biodistribution (% total dose/organ) was:

| | liver | kidney | tumour image seen |
|---|---|---|---|
| Without pre-dosing | 23.8 | 1.0 | 9/12 evaluable pts |
| With pre-dosing | 17.7 | 1.8 | 4/7 evaluable pts |

Pre-dosing with cold antibody reduced liver uptake in the split dosing part of the study. In a forthcoming therapy study, hCTMO1 will be used conjugated with a cytotoxic drug.

### O18 VASCULARITY & THYMIDYLATE SYNTHASE LEVEL INFLUENCE COLORECTAL LIVER METASTASES RESPONSE TO FLOXURIDINE.

MM Davies[1], D Burke[1], S Kaur[1], PG Johnston[2], TG Allen-Mersh[1].
[1]Department of Surgery, Charing Cross & Westminster Medical School, Chelsea & Westminster Hospital, London & [2]Department of Oncology, The Queen's University of Belfast.

Drug delivery (tumour vascularity) and tumour resistance (thymidylate synthase activity) may both be important in determining response to regional infusion of the fluorinated pyrimidine floxuridine.
We have examined whether of intratumoural thymidylate synthase and vascularity correlate with response to FUDR, by staining liver metastasis biopsies with anti-thymidylate synthase (TS106) and an anti-endothelial (JC70) antibodies prior to intrahepatic FUDR treatment of colorectal liver metastases.
Response was determined by comparing CT scans at the start and after four months of treatment. The intensity of TS106 staining was assessed by two "blinded" observers as either low (staining absent or weak) or high (staining moderate or intense). Vascularity was assessed by a graticule counting method at x200 magnification.

| | n (patients) | Response | No response | p |
|---|---|---|---|---|
| **TS106** High | 12 | 3 | 9 | 0.049 |
| (patients) Low | 12 | 8 | 4 | |
| **JC70** (Vessel Count median,i.q.r.) | 20 | 39.4 (25.4-51.9) | 21.2 (12.5-34.8) | 0.048 |

Vascularity was significantly (p=0.02) lower (median 15.2 i.q.r. 11.2-22.0) in low TS staining tumours which did not respond compared with those (median 39.4 i.q.r. 23.3-49.3) which responded.
Response by CLM to FUDR was associated with both lower TS levels and higher tumour vascularity.

CLM Colorectal Liver Metastases FUDR Floxuridine
TS Thymidylate Synthase

### O19 DOES THE HEREDITARY PROSTATE CANCER GENE, HPC1 CONTRIBUTE TO A LARGE PROPORTION OF FAMILIAL PROSTATE CANCER? - RESULTS FROM THE CRC/BPG UK & CANADIAN CONSORTIUM.

R. Eeles[1,2], J. Simard[3], D. Teare[4], S. Edwards[1], D. Easton[4], D. Dearnaley[2], F. Durocher[3], R. Shearer[2], A. Ardern-Jones[2], A. Dowe[2], 105 collaborators[5], S. Narod[6], W. Foulkes[7].
Institute of Cancer Research[1] and Royal Marsden Hospital[2], Sutton; Institute of Public Health, Cambridge[4]; The CRC/British Prostate Group Study Collaborators[5]; UK and CHUL Research Centre, Quebec[3]; Cancer Genetics, Toronto[6] and Montreal General Hospitals[7], CANADA.

There is evidence that prostate cancer has a genetic component; this is demonstrated by the familial clustering of cases, the increased relative risk of the disease in relatives of cases and the marked rise in this level of risk as the clustering of cases increases in closeness of relationship or number of affecteds. It has been estimated that up to 43% of cases at <55 years may be due to a highly penetrant gene. Recently the first susceptibility prostate cancer locus (HPC1) was mapped to chromosome 1q34 by linkage in a study of 91 families, most of which had 4 or more prostate cancer cases (Smith et al, 1996). However, most clusters in collections of familial prostate cancer worldwide have 2-3 cases. The CRC UK familial prostate cancer study is the largest collaborative UK effort to collect prostate cancer families, and has 105 collaborators via the British Prostate Group (BPG). A collaborative Canadian effort has combined expertise and familial collections from 3 Canadian centres. Within this consortium, 140 families with ≥ 2 cases of prostate cancer have had blood DNA extracted. Only 15 clusters had ≥ 4 cases. Four genetic 1q markers, spanning the region within which HPC1 lies, were run to assess linkage in these families. Overall, there is no significant evidence of linkage to 1q in these families; the total heterogeneity Lod score was 0.12 with an expected 11% of families due to HPC1. This is lower than the original 34% described. We therefore conclude that HPC1 contributes more signficantly to larger prostate cancer clusters and that the majority of smaller clusters of ≤3 cases are due to other gene(s).
This study is supported by the Cancer Research Campaign, UK and MRC of Canada.
(Smith et al 1996: Science. 274:1371).

### O20 DO BISPHOSPHONATES EFFECT TUMOUR BULK? CORRELATION OF TUMOUR MARKER, CLINICAL AND BIOCHEMICAL EFFECTS.

R. Coleman[1], J. Vinholes[1], O. Purohit[1], A Milford-Ward[2]. YCRC Dept of Clinical Oncology, Weston Park Hospital and Dept of Immunology, Northern General Hospital, Sheffield.

Bone resorption results in the release of various cytokines and growth factors which may provide an attractive environment for tumour growth. Pamidronate is a potent inhibitor of bone resorption which reduces the skeletal morbidity and bone pain associated with metastatic bone disease. We have evaluated serum levels of the tumour markers CA15-3 and PSA as surrogates for tumour bulk, and Ntx as a specific marker of bone resorption in patients with breast (n=29) and prostate cancer (n=10) respectively. Patients were randomised to receive intravenous pamidronate 120 mg or a placebo infusion. Four weeks later all patients received pamidronate 120 mg. Serum was collected every 4 weeks for tumour markers and urine every 2 weeks for bone resorption markers during the 12 week study period. During this time no systemic anticancer treatments or radiotherapy were administered.
CA15-3 was elevated before treatment in 19 (66%) breast cancer patients and PSA in 9 (90%) of prostate cancer patients. A >10% fall in tumour markers was seen in 11 patients (39%) (8 breast and 3 prostate) after pamidronate. This was only seen once (10%) with placebo. Changes within the normal range were considered uninterpretable. 10 patients (91%) with a >10% fall in tumour markers reported a >20% improvement in pain score - derived from an amalgam of pain intensity, analgesic consumption and performance status - compared with only 3 (18%) who showed no change or a rise in markers (p=<.01). Ntx fell to normal (n=7) or was normal throughout treatment (n=3) in all the 10 clinical responders showing a fall in tumour markers of >10%
These data support the observations in animal models of a link between bone resorption rates and tumour growth. The possibility that bisphosphonates have an anticancer action as well as beneficial effects on skeletal health deserves further study.

### O21

### TRANS-RECTAL ULTRASOUND GUIDED IODINE-125 IMPLANT FOR LOCALISED CANCER PROSTATE

**AUTHORS:** H. Al-Booz, D. Ash, B. Carey, A. Flynn. Cookridge Hospital, Leeds **Object** This work is done to determine the acceptability and short-term side effects of TRUS- guided I-125 implant for early cancer prostate. **Methods** 50 patients under the age of 75, with clinically and radiologically confined carcinoma of the prostate, and a prostate volume of less than 50 cc were treated at Cookridge Hospital, Leeds, with TRUS-guided iodine-125 Implant. Short-term side effects; urinary outflow score, bowel dysfunction, potency, and quality of life assessment (according to the EORTC- QLQ-30) were assessed. **Results** After the first 12 patients who were hospitalised for 4 days, the remainder had a mean stay of 2.5 days.Two months post implant 35.5% of patients had mild frequency of micturition, 27% moderate, non severe, and no patients had incontinence. At six months 15% of patients still complained of occasional mild nocturia, hesitancy, slight reduction in urinary stream, and none had incontinence. No patients complained of bowel symptoms. At six months 54% of patients had retained potency, of these some had not yet regained full potency after prior hormone therapy. Almost all patients had a fall in PSA. At 2 months 53% had a PSA less than 4 ngm/ml. At six months 73% had a PSA less than 4. All patients who work had returned to full-time employment within 3 weeks of implantation. **Conclusion** Results show that the procedure is completed within a minimum of hospitalisation and with few side effects. It is early to assess local control or late morbidity.

### O22

### STILBOESTROL IN THE MANAGEMENT OF REFRACTORY METASTATIC PROSTATE CANCER

D Dearnaley[1], A Capp[1], J Gadd[2], R Huddart[1].

[1]Academic Unit ,[2] Bob Champion Unit, Royal Marsden Hospital, Sutton, Surrey, SM2 5PT.

**Patients and Methods**

48 patients with metastatic Ca Prostate were prospectively entered into a phase II study using Stilboestrol 3mg/day with 75mg aspirin/day. All patients had previously been treated by orchidectomy or LHRH analogues +/- additional hormone manoeuvres. LHRH analogues were discontinued on commencing Stilboestrol. All patients had rising PSA levels +/- subjective evidence of disease progression. Stilboestrol treatment was 2nd line therapy in 2 patients, and given as 3rd or 4th line in 29 and 17 patients respectively. Stilboestrol was continued until there was subjective (increased levels of pain/decreased performance status/requirement for palliative radiotherapy)or objective progression. The mean age at commencement of therapy was 67 years (range 48 - 86 years).

**Results**

1. Subjective response. 23/48 patients (48%) of patients showed benefit or disease stability. Median duration of treatment was 4 months (range 0 - 18 months ).
2. PSA response. Median PSA at the commencement of stilboestrol was 733ng/ml (range 11 - 12950ng/ml).39/48 patients had some fall in PSA level. In these 39 men, mean PSA fall was 62% (range 10 - 99%) with median time to PSA nadir of 1 month (range 2 weeks - 12 months). 26/48 men (54 %) had ≥ 50% fall in PSA. 12/48 (25%) had ≥ 80% fall in PSA.

No subjective benefit was seen in 9/48 men who had rising PSA levels (median survival 1.5 months). 4/13 men with < 50% fall inPSA had subjective benefit ( median survival 7 months) compared with 19/26 men with ≥ 50% PSA fall (median survival 15 months). Subjective benefit was seen in 11/12 patients with ≥80% PSA fall (median survival 18 months).

10 patients remain alive, and 3 are currently continuing to take Stilboestrol.

3. Toxicity. 5 patients experienced thrombo-embolic events on Stilboestrol : 2 x DVTs, 2 x pulmonary emboli, and 1x axillary vein thrombosis. 4 responding patients continued on Stilboestrol whilst fully anticoagulated without further thrombotic episodes. There were no deaths from thrombo-embolic events. Additionally, 5 patients with previous histories of thrombo-embolic events were fully anticoagulated whilst on Stilboestrol, with no further complications.

**Conclusion**: Stilboestrol provides useful palliation in up to 50% patients with refractory disease for a median time of 5 months, with a minority of patients 7/48 having prolonged responses of a year or more.Provided each patient is anticoagulated according to individual risk, thrombo-embolic events are uncommon.

### O23

### CARBOGEN OR HYPERBARIC OXYGEN FOR BLADDER CANCER - UPDATED PHASE II RESULTS AND ANALYSIS OF THE SECOND MRC HYPERBARIC OXYGEN TRIAL.

Hoskin PJ, Saunders MI, Verma N, Powell MEB, Goodchild K, Dische S. MCRW, Mount Vernon Hospital,Northwood, Middx UK.

A total of 35 patients (mean age 68.5, 29 male) have been treated with radical radiotherapy delivering 50-52.5Gy in 20 daily fractions to the bladder whilst breathing carbogen (95% oxygen, 5% $CO_2$). Sixteen of these patients have in addition taken oral nicotinamide 80mg/kg daily 2 hours prior to radiotherapy. A total of 26 patients are assessable by cystoscopy at least 6 months following radiotherapy. Of these 24 (89%) have documented complete regression of intravesical tumour. Three local relapses at subsequent check cystoscopy have occurred these being seen at 10 months, 11 months and 30 months. Acute morbidity from treatment is no greater than expected without carbogen breathing and only one patient has significant late morbidity with a fibrosed shrunken bladder requiring catheterisation. There have been 9 deaths in this series of 35 patients (primary bladder tumour 3, metastatic bladder cancer 3, incidental death 3).

This series has been compared with an updated analysis of the second MRC hyperbaric oxygen trial in bladder cancer. In this study 116 patients were treated with radical radiotherapy delivering 48Gy in 14 fractions in air (56 patients) or 32Gy in 6 fractions in hyperbaric oxygen (60 patients). The original findings of the MRC bladder trial are confirmed on 15 year follow-up showing no difference between the two arms of the trial for recurrence-free interval or overall survival. Over 50% of bladder relapses in this trial were seen in the first year after radiotherapy during which period the current Mount Vernon carbogen series is demonstrating considerably better results, although overall survival curves can be superimposed during this initial period. The limitations of historical comparison are recognised together with the relatively short observation period in the carbogen series but this data suggests a possible advantage for carbogen for local control of bladder cancer.

### O24

### JUSTIFICATION FOR USE OF CHEMOTHERAPY TO CONSERVE THE TESTIS IN PATIENTS WITH GCC.

RTD Oliver, J Ong, S Asterling. Medical Oncology, St Bartholomew's & The Royal London Hospitals, West Smithfield, London EC1A 7BE.

Given the association between declining sperm count and rising testis cancer incidence and that more than 2/3rds of germ cell cancer patients have diminished sperm counts and more infertility than matched controls, attempting to conserve normal germinal epithelium in these patients is justified if it does not diminish cure. Recently, this unit has demonstrated that chemotherapy can salvage the primary tumour bearing testis in some patients who present with metastases or have a tumour in a solitary testis. To justify extending these studies, data on delay and primary tumour size in a series of 453 patients and the effect of chemotherapy on incidence of tumours in the contralateral testis is reviewed and the study on the response of primary tumour to chemotherapy is updated.

Between 1978-83 (n=111) 16% presented with delay equal or less than 2 months while in the period 1989-94 (n-190), 31% presented with delay less than 2 months. The average size of testis tumours removed after less than 2 months delay was 3.8cms while those removed after more than 6 months delay had a median tumour size of 5.3cms. The actuarial risk of a second germ cell cancer in patients in complete remission at 5 & 10 years after BVP for metastases (n=38), BEP for metastases (n=123), surveillance (n=72), adjuvant chemotherapy (n=138) was 4.3% & 8.9%, 1.3% & 4.6%, 5.7% & 9.9% and 1.4% and 1.4% respectively. To date 46 patients have received testis conserving chemotherapy. 30 had residual abnormality at completion of chemotherapy and proceeded to orchidectomy. 16 have been followed for a median of 7 years without orchidectomy with one second cancer at 12 years.

Given the poor results of hormone replacement in men, these findings suggest that routine use of orchidectomy needs to be reconsidered. Neo-adjuvant chemotherapy +/- partial orchidectomy is an alternative in patients with testis atrophy or tumour in a solitary testis. To date 3 of 6 patients with tumours in a solitary testis have recovered spermatogenesis ad 1 has had 2 children.

### O25 C-BOP/BEP FOR INTERMEDIATE AND POOR RISK GERM CELL TUMOURS. A SINGLE INSTITUTION EXPERIENCE.

DWO Tilsley, S Rivers, P Gill, D Moreley, K Owen and MD Mason, Velindre Hospital, Cardiff CF4 7XL.

**Objective:** C-BOP/BEP has been reported previously as a promising regime in germ cell tumours at high risk of relapse with standard therapy[1]. We report a single centre experience of C-BOP/BEP in intermediate and poor risk germ cell tumours.

**Methods:** Intensive induction is followed by consolidation with 3

| week | 1 | 2 | 3 | 4 | 5 | 6 |
|---|---|---|---|---|---|---|
| Cisplatin 20mg $m^{-2}$ | xxxxx | | xxxxx | | | |
| Cisplatin 40mg $m^{-2}$ | | x | | x | | |
| Carboplatin AUC × 2 | x | | x | | | |
| Vincristine 2mg | x | x | x | x | x | x |
| Bleomycin 15mg/24 hrs | | xxxxx | | xxxxx | | |
| Bleomycin 15mg/15 min | x | | x | | x | x |

cycles of BEP on weeks 7, 10 and 13 (total Bleomycin dose 345mg).

**Results:** Between Dec. 93 and Dec. 96 15pt. (13 testicular teratomas, 2 seminomas) were treated. 9 were IGCCCG poor risk and 5 intermediate risk. 14 were first presentations and 1 an 11 yr BEP relapse. The mean age at start of C-BOP/BEP was 30(21 – 47) yr. 11/15 (73%) are alive and disease free with a median follow-up of 15(1 – 37) months. 2 underwent surgery and only necrotic tumour was found. 2 received RT and 1 elective high dose chemotherapy. 1 pt. died of bleomycin lung and 1 of neutropenic sepsis. 1 tumour death occured at 12 months from brain metastases. The main toxicities were leucopenia (58% G3, 25% G4), thrombocytopenia (25% G3 25% G4) and neuropathy (50% G1, 33% G2). The GFR remained normal in 10/12 but in 2 was 69 and 79 ml $min^{-1}$ at the end of chemotherapy.

**Conclusion:** C-BOP/BEP appears effective in the treatment of intermediate and poor risk germ cell tumours. Further experience of this regime in a multi-centre phase II trial is warranted.

**Reference:** Horwich A *et al.* Eur. J. Cancer **30A**, 1607–1611 (1994).

### O26 ENTRY INTO CLINICAL RESEARCH STUDIES: PATIENTS WHO REFUSE.

Owen J; Abbey M; Briggs T; Browne S; Hutchinson J; Wardle J; Coleman R & Hancock BW. YCRC Dept. of Clin. Oncol., Weston Park Hospital, Sheffield.

**Introduction.** The right of a patient to refuse entry into a clinical research study is an integral part of good clinical research practice. We describe an audit undertaken at Weston Park Hospital, Sheffield investigating the numbers of patients who refuse entry, the types of studies they were offered entry into and their reasons for refusal.

**Method.** Patients who refused entry into a clinical trial were evaluated between January 1996 and January 1997. Name, hospital number, diagnosis, study referred for and reason for refusal were documented.

**Results.** 421 patients were entered into research studies in the specified period. An additional 80 patients refused entry, 36 (45%) male, 44 (55%) female. 37 (46%) refused entry into adjuvant treatment trials, 10 (13%) refused 1st line therapy studies, 13 (16%) refused entry into 2nd line studies and 20 (25%) refused trials involving supportive therapies. 29 (90%) of the trials refused were randomised studies and 3 (10%) were open studies. Reasons for refusal were:- 24 (30%) did not want chemotherapy (adjuvant treatment), 6 (7.5%) did not want to be randomised, 20 (25%) had a definite treatment preference, 9 (11%) refused due to the extra time involved, 5 (6%) requested no further treatment (advanced disease), 7 (9%) did not want to be 'experimented on', 3 (4%) felt too unwell, 3 (4%) refused due to potential treatment toxicity, 1 (1%) was needle phobic and for 2 (2.5%) patients the reasons were unknown.

**Conclusion.** Patients do not have to give a reason for their refusal to enter a study but an indication of why may be of benefit to clinicians and allow us to tailor our process of obtaining written informed consent accordingly.

### O27 AIM HIGH - FIRST CONTACT

S. Harris, B.W. Hancock, for the United Kingdom Co-ordinating Committee on Cancer Research, YCRC Dept of Clinical Oncology, Weston Park Hospital, Sheffield

There are no time limits imposed on investigators wishing to participate in multicentre studies regarding the interval between initial enquiry, submitting the study to their local Ethics Committee and entering the first patient into the trial.

The AIM HIGH study is a United Kingdom Co-ordinating Committee on Cancer Research (UKCCCR) multicentre, randomised study of observation versus adjuvant low dose extended duration interferon alpha-2a in high risk resected malignant melanoma.

Correspondence to and from the Clinical Trials Office was used to establish time taken for investigators to submit the protocol to their Ethics Committee after their initial enquiry, time taken to inform the trial co-ordinator that approval had been granted and time taken to enter the first patient into the study.

22 centres were studied. 9 Teaching Hospitals, 8 District General Hospitals and 5 Specialist Oncology Centres. From initial enquiry to submission to Ethics Committee takes an average of 4 months. For the investigator to be notified that the study has received approval takes 4 days but it takes 44 days for the co-ordinator to receive written confirmation of this. After approval, it then takes, on average, 101 days to recruit the first patient into the trial.

Time taken from initial enquiry to entry of the first patient has implications for the recruitment of a study which could be delayed by as much as 7 months. Perhaps there should be guidelines on reasonable time limits for this process.

### O28 RADIOTHERAPY FRACTIONATION SENSITIVITY: PROPOSALS FOR A UK NATIONAL TRIAL.

JR Yarnold[1] JR Owen[2], JM Bliss[3], SR Ebbs[4], J. Regan[1], B. Broad[2], J. Davidson[3], G. Harrington[2], A. Ashton[2].

[1]Academic Radiotherapy Unit, The Royal Marsden NHS Trust, Sutton, UK; [2]Gloucestershire Centre for Clinical Oncology, Cheltenham, Glos, UK; [3]Section of Epidemiology, Institute of Cancer Research, Sutton, UK; [4]Department of Surgery, Mayday Healthcare, Croydon, UK.

The fractionation sensitivity of squamous cell carcinomas of the head and neck and cervix uteri is relatively low with $\alpha/\beta$ ratios comparable to those of early reacting normal tissues. Very few data exist for other tumour types, but those for soft tissue sarcomas, melanomas and breast cancer suggest that fractionation sensitivities may be greater in some tumour types. If this can be confirmed for breast cancer, the possibility exists of simplifying radiotherapy schedules for a very large population of patients without compromising treatment benefit. Between January 1986 and July 1994, 915 patients were entered into a trial after complete resection of early stage breast cancer. Two 13-fraction schedules were compared with a standard schedule of 50Gy in 25 fractions to the breast. Two dose levels of the 13-fraction regime were selected (42.9Gy and 39Gy) with resulting fraction sizes of 3.3 and 3.0Gy respectively. The duration of treatment was 5 weeks in all arms of the trial, followed by an electron boost to the tumour bed of 14 Gy in 7 daily fractions. Assessments of normal tissue damage in the breast were made by comparing the appearances of annual photographs with the post-surgical appearances. Of 735 patients with at least one follow-up photographic assessment, the probability of a change in breast appearance is significantly lower in patients randomised to 39Gy in 13 fractions compared to those allocated 50Gy in 25 fractions or 42.9Gy in 13 fractions. At this time, no evidence is observed of a difference between the 50Gy and 42.9Gy arms. It is therefore planned to extend accrual of the trial to 2,500 patients in order to compare tumour control and the frequencies of uncommon late side-effects.

### O29 SERUM LIPIDS AND LIPOPROTEINS IN WOMEN WITH EARLY STAGE BREAST CANCER: EFFECTS OF HRT AND TAMOXIFEN.

J Marsden, NPM Sacks, M Worthington, D Crook. Dept. of Surgery, The Royal Marsden Hospital & The Wynn Division, ICSM, London.

**The greatest non-oncological cause of mortality in breast cancer survivors is cardiovascular disease. HRT reduces cardiovascular risk in part through changes in lipids and lipoproteins. Tamoxifen lowers total and LDL cholesterol levels, consequently, as part of a randomised study of HRT use in women with breast cancer, we investigated the combined effects of tamoxifen and HRT on serum lipids and lipoproteins.**
METHODS: **One-hundred postmenopausal women with early stage breast cancer, experiencing vasomotor symptoms were randomised to receive HRT (unopposed oestradiol or sequential combined therapy as appropriate) or no HRT for 6 months. Fasting levels of total cholesterol, triglycerides, LDL, HDL, HDL subtypes and apolipoproteins AI, AII and B were measured before randomisation and after 6 months. Of the 86 women eligible for this lipid sub-study, 41 were current tamoxifen users.**
RESULTS: **At baseline, breast cancer patients appeared to have high levels of total and LDL cholesterol and apolipoprotein B compared to an age and weight-matched group of healthy postmenopausal women. Patients taking tamoxifen had normal levels. In breast cancer patients HDL, apolipoprotein AI and II levels were normal at baseline, irrespective of tamoxifen status. After 6 months, HRT had not altered LDL levels in the tamoxifen group, whereas these levels fell by 10-15% ($P<0.05$) in those not taking tamoxifen. HDL cholesterol levels increased slightly in the tamoxifen group treated with unopposed oestradiol but tended to fall in women given combined therapy, regardless of their tamoxifen status.**
CONCLUSION: **The use of HRT in breast cancer patients already treated with tamoxifen may not confer all of the protection against cardiovascular disease expected from studies of HRT in healthy women.**

### O30 A NATIONAL RANDOMISED TRIAL OF HRT IN SYMPTOMATIC WOMEN WITH EARLY STAGE BREAST CANCER.

J. Marsden & NPM Sacks on behalf of the HRT Trial Steering Committee*

Observational studies of HRT use in breast cancer patients have not shown an adverse effect on survival however, a large randomised trial is urgently needed. Although a previously reported randomised pilot study[1] had a high accrual of 40% suggesting a national trial would be feasible, the opinions of patients (in an R&D funded project undertaken by The Consumers' Advisory Group for Clinical Trials and the Patient Involvement Unit, Mount Vernon Hospital) and breast cancer surgeons (by postal questionnaire) were sought to identify issues relevant to the design and successful implementation of a national trial. Patient concerns about trial participation centred around principles of good clinical practice and provision of information as well as the use of HRT, highlighting that reliance on accrual alone as a measure of successful trial design is inappropriate as it does not elucidate factors influencing patient choice. The majority (73.4%) of the 125 (i.e.50%) responding breast cancer surgeons supported a randomised trial but only 28% of these admitted to clinical uncertainty about whether HRT stimulates recurrence.
The proposed national trial aims to recruit 3,000 postmenopausal women with early stage breast cancer, experiencing vasomotor symptoms, randomising them to receive HRT or not for a minimum of two years. The main end points are (1) disease-free survival and (2) quality of life, follow-up being organised so as to inflict a minimal additional workload on clinics. Importantly, the preliminary work has resulted in a considerable proportion of the trial design being devoted to the support and information needs of both patients and health professionals before, during and after the trial period and is thus anticipated to optimise recruitment, save time and resources and is, in this respect, unique.

*Steering Committee Members (NPM Sacks, J Marsden, J Bliss, R A'Hern, J Maher, IE Smith, MIW Whitehead, M Baum, D Riley, K Thirlaway, H Thornton, M Dowsett, H Barton, Marie-Ann de Boeklin, D Fenlon, A Kirby)

1. Marsden J. et al 1996. Br J Cancer (74) Suppl 18, SP14 p6.

### O31 TAMOXIFEN: ASSESSMENT OF THE BALANCE OF BENEFITS AND RISKS OF LONG-TERM TREATMENT.

Richard Gray, Kathleen Milligan, Lauren Padmore on behalf of the aTTom trial steering committee. CRC Trials Unit, Medical School, University of Birmingham.

The most recent results from randomised studies comparing different durations of tamoxifen treatment suggest that 5 years treatment is better than 2 years but substantial uncertainty remains about whether longer than 5 years will provide any extra benefit. The NSABP B-14 study comparing 5 years with 10 years found a higher recurrence rate with longer treatment whereas a similar ECOG study has reported an opposite finding. These apparently incompatible results may be at least partly explained by the small numbers of events seen in these two studies - compounded by the outcome-dependent early closure of the NSABP study. A meta-analysis of the published data from tamoxifen duration studies has been undertaken and the results of this, in the context of the EBCTCG world-wide overview of tamoxifen trials, will be presented. These data suggest that even longer treatment with tamoxifen will produce some additional net benefit but much larger scale randomised evidence is needed to confirm this.
aTTom is a large, uniquely, simple clinical trial that aims to provide definitive evidence on the balance of benefits and risks of long term tamoxifen by randomising many thousands more women between stopping tamoxifen and continuing for 5 extra years. Because of divergent clinical opinion on the minimum prior duration of tamoxifen therapy, aTTom has adopted a pragmatic approach with randomisation occurring at the point when both the clinician and patient become *uncertain* whether to stop or continue treatment. We surveyed UK tamoxifen prescribing patterns in 1993 and, again, in 1997. The results of this survey and the shift in UK breast cancer specialists minimum treatment period, as evident from randomisations into aTTom, will be presented and discussed.

### O32 NEAT-A NATIONAL BREAST CANCER STUDY OF EPIRUBICIN+CMF VS CLASSICAL CMF AS ADJUVANT THERAPY.

HM Earl, CJ Poole, D Spooner, C Jevons, and J Dunn. CRC Clinical Trials Unit, Univ. of Birmingham, B15 2TA

The EBCTCG overviews of adjuvant chemotherapy for early stage breast cancer have demonstrated a convincing reduction in the annual odds of disease recurrence and of death with the addition of adjuvant chemotherapy[1]. However, important questions of what might constitute optimal adjuvant chemotherapy for early stage breast cancer remain unanswered. One of the most pressing questions is the role and optimal scheduling of anthracyclines. Studies by Buzzino and Bonadonna found a significant benefit when anthracyclines were given prior to CMF[2,3]. However, other studies of anthracycline-based regimens have shown no statistically significant improvements in disease free or overall survival when compared to classical CMF[4].
NEAT (and its Scottish counterpart, SCTBG study of adjuvant chemotherapy) is a multi-centre randomised trial of anthracycline-containing chemotherapy exhibited in a block schedule followed by sequential classical CMF, compared with classical CMF alone. Epirubicin has been selected as the anthracycline of choice because its anti-tumour effect is equivalent to that of doxorubicin but it has a lower general toxicity, in particular the cardiac toxicity is about 50% that of doxorubicin.
Target accrual is 1,000 in total, and already 150 patients have been randomised by over 20 collaborators. As well as comparing the effects of these two chemotherapy regimens on recurrence free and overall survival, we shall also be assessing their impact of this trial on clinical practice. We are asking clinicians at set time points their expectations of the treatment effects and the first data available will be discussed. NEAT is funded by the Cancer Research Campaign.

1. EBCTCG, (1992) Lancet: **339** pp1-15, 71-85
2. Bonadonna G, et al. (1995) JAMA **273(7)** pp542-547
3. Buzzoni R, et al. (1991) JCO **9** pp2134-2140
4. Budd, GT, et al. (1995) JCO **13(4)** pp831-839

### O33 PHASE II TRIALS IN ANAL CANCER: 5-FU AND CDDP IN ADVANCED DISEASE AND IN EARLY DISEASE IN COMBINATION WITH RADIOTHERAPY.

HM Meadows, CM Ryan, J Houghton, JMA Northover on behalf of the UKCCCR Anal Cancer Working Party

The first UK randomised trial comparing radiotherapy alone with chemoradiation in patients with anal cancer found a significant advantage in local control for patients on the combined modality treatment[1]. However the results also demonstrated that these patients have a poor prognosis with about 50% dead by 5 years and, of those dying of the disease, more than 40% had distant metastastes.

To improve this outcome we planned to run a further phase III trial using cisplatin (CDDP) and 5-fluorouracil (5-FU). To evaluate the toxicity of these agents two phase II trials have been initiated. The first was to evaluate 4 cycles of 5-FU (1 g/m² per day) and CDDP (25 mg/m² per day) both for 4 days, given every 3 weeks, in patients with advanced disease. Eleven patients have been recruited; median age 49 (38-66) years. Seven patients reported toxicity (4 neutropaenia ≥ grade 3; 2 WBC ≥ grade 3; 2 alopecia; 1 stomatitis) and there was 1 treatment-related death.

A second phase II trial has evaluated the same doses of chemotherapy given in one or two courses concomitantly with radiotherapy (40 Gy in 20 fractions in 4 weeks or 45 Gy in 25 fraction in 5 weeks) in newly diagnosed patients. 25 patients have been entered; median age 53 (36-71) years. In the 16 patients for whom response information is available, 13 had a complete response and 3 a partial response. No severe toxicity was reported in 10 patients. Ten patients reported skin reactions but these were only severe (≥ grade 3) in 4.

A further phase II trial is underway to evaluate chemoradiation incorporating mitomycin as well as 5-FU and CDDP.

1. UKCCCR Anal Cancer Working Party. Lancet 1996 ;348: 1049-54

### O34 THE INCIDENCE AND EFFECT OF PROLONGATION OF OVERALL TREATMENT TIME IN PATIENTS WITH CARCINOMA OF THE LARYNX RECEIVING RADICAL RADIOTHERAPY IN SCOTLAND.

A G Robertson(1), C Robertson(2), H M MacDougall(3), H Yosef(1), J Dewar(4), T Elia(5), D Hurman(6), C Clark(7), C Peronne(2). 1) Beatson Oncology Centre, Western Infirmary, Glasgow, G11 6NT, Scotland. 2) EIO Milan. 3) Radiotherapy Dept,Western General, Edinburgh. 4) Radiotherapy Dept, Ninewells Hosp. Dundee. 5) Radiotherapy Dept, Inverness. 6) Radiotherapy Dept, Foresterhills Hosp. Aberdeen. 7) SCTN ISD Edinburgh.

1071 patients with cancer of the larynx were treated in the five Scottish Cancer Centres between 1986 and 1990, 1994 and 1995 with radical radiotherapy. The male to female ratio was 3.85:1. Data relating to tumour stage, field size and treatment schedule were all recorded. 97% were treated wearing a BDS. Three Centres treated patients radically over 4 weeks and the other two over 5 and 6 weeks. After allowance for patient characteristics, stage of disease etc. there was no significant difference in outcome for patients treated at the five centres. Patients with tumours arising in the Glottic region had a better outcome than those with tumours arising in the supraglottic region (67-84%-v-26-55%). 65% of patients had their treatment prolonged by a day or more. 50% of these interruptions were due to machine servicing or machine breakdowns. Other reasons included replanning treatment, transport problems and patient illness. Survival analysis of the complete data (912 cases) gave a five year survival for T1-78%, T2-66%, T3-46%, T4-38%. Linear quadratic analysis for the glottic node negative showed that unscheduled prolongation of treatment affected overall tumour control. Prolongation by a day is statistically significant. In many cases a one day gap resulted in a prolongation of 3 days due to the addition of a two day weekend gap. The effect of prolongation of overall treatment time by 4 days is very significant.

### O35 SITES OF RECURRENCE AFTER LIMITED NECK IRRADIATION (XRT) FOR CANCER OF THE SUPRAGLOTTIS AND TONSIL.

J.H. Hay, S.M. Jackson, A.D.Flores, L.M. Weir, F.W.L. Wong, C. Schwindt, B. Baerg. BC Cancer Agency, Vancouver Cancer Centre, 600 W 10th Ave, Vancouver, British Columbia, V5Z 4E6, Canada.

Aim: to analyse the sites of failure following XRT for scc tonsil and scc supraglottis when the target volume included only clinical disease and the next echelon of lymph nodes.
Study populations. All received at least 50Gy, most 60Gy in 2.4Gy fractions. Supraglottis:121 patients (88% of all radically treated patients) treated between 1985 & 1992, all with opposed lateral fields, none had the posterior neck irradiated above the limits of cord tolerance. 15 had UICC Stage I disease, 25 St II, 34 St III, 47 St IV. Average field lengths were St I: 7.2cm, St II: 7.5cm, St III: 8.5cm and St IV: 9.2cm. Tonsil 88 patients treated with unilateral XRT in the years 1975-1980 and 1988-1993. The two cohorts had the same local control and survival rates. 8 were UICC St I, 28 St II, 34 St III and 18 St IV. Average field lengths were St I: 7.25cm, St II: 8.3cm, St III: 9.3cm and St IV: 11cm.
Results: Overall survival and local control compared favourably with the reported literature.

| | Site of progression or recurrence | | |
|---|---|---|---|
| | Primary | Nodes | Both |
| Supraglottis | 30 | 12 | 10 |
| Out of field | 1 | 4 | |
| Tonsil | 21 | 9 | 4 |
| Out of field | 0 | 2 | |

Conclusion. Limited field XRT of this sort results in nodal recurrence rates that are very similar to those obtained by wide field XRT and are lower than those reported after small field XRT. In-field locoregional failure is the major problem.

### O36 IMPACT OF CONFORMAL RADIOTHERAPY ON TREATMENT OF HEAD AND NECK CANCER,

M H Robinson*[2], J Conway[1], C Antony[2], G Brown[2], L Griffiths[1] and S Otter[1], [1]Department of Medical Physics and Clinical Engineering, [2]YCRC Department of Clinical Oncology, Weston Park Hospital, Sheffield

Introduction: the treatment of head and neck cancer with radiotherapy is difficult because of the low tolerance to radiation of many structures which are close to the target volume. 3D conformal radiotherapy is being introduced in an effort to improve the therapeutic ratio in this situation.
Methods: conformal 3D plans produced via CT studies and utilising Multileaf Collimation (MLC) of each field are compared against conventional planning techniques where planning target volumes (PTVs) are defined from orthogonal radiographs and where simple shielding methods are employed. Analysis of cumulative dose volume histograms (CDVH) for both plan types, enable four score functions to be defined, ie gradient ($^{S}o$), overdose ($^{S}1$), underdose ($^{S}2$) and regret fraction ($^{R}f$). The regret fraction is defined as the ratio of the PTV to the Treated Volume enclosed by the Minimum Tolerance Dose (Dmin) and indicates the degree of conformality of the plan. The other score functions reflect the uniformity of dose, overdose, and underdose volumes relative to the tolerances allowed in the PTV. The dose to critical organs are compared for both conformal and conventional plans.
Results: 37 patients have been studied. An expected improvement in the degree of conformity using MLCs was demonstrated in 70%. 60% of these patients plans showed a reduction in the overdose volume for the conformal plans. However, half of all the plans exceeded the tolerances for overdose in the target volume. 40% of plans showed increase in the underdose volume when multileaf collimator fields are employed but remained within the limits decided by the clinician. Those plans that had poor underdose scores using conventional planning were improved using MLC fields. 70% of plans indicated a reduction in mean dose to critical structures.
Conclusion: This modified scoring system provides a means to judge conformal planning techniques against the conventional methods that have previously been employed.

### O37 PHASE I DOSE ESCALATION TRIAL OF INTRA-TUMOURAL INJECTION OF AN ONCOLYTIC E1B ATTENUATED ADENOVIRUS, ONYX-015, IN PATIENTS WITH RECURRENT p53(-) SQUAMOUS CELL CANCER OF THE HEAD AND NECK.

I. Ganly[1,2], D. Kirn[6], G.I.Rodriguez[3], D. Soutar[2], G. Eckhardt[3], R. Otto[4], A.G.Robertson[1], O. Park[1], M.L. Gulley[5], M. Kraynak[5], C. Heise[6], C.Maack[6], P.W.Trown[6], D.D. Von Hoff[3], S.B.Kaye[1]. [1]University Dept of Medical Oncology, Beatson Oncology Centre, Glasgow; [2]Dept of Plastic and reconstructive surgery, Canniesburn hospital, Glasgow, Scotland; [3]Cancer Therapy and Research Center, San Antonio,TX78229; [4]The University of Texas Health Science Centre at San Antonio, TX 78284; [5]Audie L. Murphy VA Hospital, San Antonio,TX78284; [6]ONYX Pharmaceuticals, Richmond, CA 94806.

**OBJECTIVE:** ONYX-015 is an adenovirus which lacks the E1B gene coding for the 55kDa protein. This allows it to selectively replicate and lyse cells with non-functional or mutant p53. The objective of this study was to determine the safety and efficacy of direct intratumoural injection of ONYX-O15.

**METHODS:** Patients with recurrent head and neck cancer were recruited. The p53 status of the tumours was assessed by immunohistochemistry and gene sequencing on a single core biopsy taken from the recurrent tumour. Only patients with abnormal p53 as assessed by IHC were treated. Patients received direct intratumoural injection of ONYX-015 over 5 cohorts with a minimum of 3 patients per cohort. Vital signs were monitored for 2 days postinjection and patients were assessed twice weekly for toxicity. Tumour response was assessed both clinically and radiologically 4 weeks postinjection. Patients with stable disease had repeat injections done every 4 weeks. Postinjection biopsies at days 8 and 22 were assayed for viral replication by in situ hybridisation of viral DNA.

**RESULTS:** 19 patients were treated - 5pts at $10^7$pfu, 4 at $10^8$ pfu, 4 at $10^9$pfu, 3 at $3x10^9$pfu and 3 at $10^{10}$pfu. All patients had abnormal p53 as judged by IHC. 13/16 tumours for which p53 sequencing results are available had mutations in exons 5-9. No significant toxicities were observed. Vital signs remained normal following injection. 5 patients received repeat treatment(up to 4 cycles). 4 patients had significant necrosis of injected tumours both clinically and radiologically. Viral replication was detected in postinjection biopsies at the $10^{10}$pfu dose level.

**CONCLUSIONS:** Intratumoural injection of recurrent head and neck squamous cell cancer with ONYX-015 is safe. In addition, clear biological effects of tumour necrosis are seen. Phase II studies are planned in order to better define the efficacy of ONYX-015.

### O38 CONTINUOUS HYPERFRACTIONATED ACCELERATED RADIOTHERAPY (CHART) AND BRACHYTHERAPY IN EARLY ORAL CAVITY AND OROPHARYNGEAL CARCINOMAS: A PROSPECTIVE REVIEW OF OUTCOME AND MORBIDITY.

Goodchild K,SaundersMI,Powell MEBP,Hoskin PJ. MCRW,Mount Vernon Hospital,Northwood,UK.

T2 N0 squamous cell carcinomas of the oral cavity may be treated with conventionally fractionated external beam radiotherapy followed by an implant. In this pilot study CHART to 40.5Gy was followed immediately by a low dose rate implant. This technique enabled the implant to be performed easily as the tumour margins were clearly defined in the absence of mucositis. Treatment was completed in 12 days.

Twelve patients aged 41-78 years were treated. Eleven had T2 N0 M0 squamous cell carcinomas and one was post-operative with no residual macroscopic disease.

The primary and first eschelon lymph nodes received 40.5Gy in 27 fractions. prescribed to the intersection point (1.5Gy/# three times daily including the weekend). CHART was commenced on a Monday morning and completed by the following Tuesday evening. A caesium or iridium implant was carried out the next morning; doses ranged from 17.5-25Gy at 0.5cm over 48 hours.

Median length of follow-up is 39.5 months (range 6-74 months). All patients developed confluent mucositis which healed completely by three months. Long term morbidity in the skin and mucosa was minimal. Three patients developed a moderately dry mouth which persisted in two.

One patient was lost to follow-up, 10/11 patients were in complete clinical remission at three months. In longer follow-up 9/11 patients were locally controlled. Median disease-free survival was 36 months and median overall survival was 39.5 months.

We conclude that this scheduling of therapy has minimal long term morbidity with good local control and survival rates.

### O39 CAN ADJUVANT PILOCARPINE PREVENT POST RADIATION XEROSTOMIA?

MEB Powell, HJ Cladd, MI Saunders, Marie Curie Research Wing,Mount Vernon Hospital,Northwood, Middx UK.

**Aim** Post-treatment xerostomia is an important toxicity of head and neck radiotherapy, leading to an increase in dental caries, oral infections, and discomfort for patients. This prospective, randomised, double blind placebo controlled trial evaluates the efficacy of oral pilocarpine given during radiation treatment in reducing the severity of salivary gland dysfunction.

**Methods** Twenty four patients with primary head and neck tumours, scheduled to receive radical radiotherapy were randomised to receive either placebo or pilocarpine (5mg TDS) to be taken from day 1 of treatment for 12 weeks. Baseline measurements of whole and stimulated parotid saliva flow rates were taken prior to treatment and at weeks 1, 8, 12, and 24 following irradiation. In addition, quality of life was evaluated using a questionnaire.

**Results** Salivary function was impaired in both groups within 1 week of starting treatment, with flow rates dropping by as much as 80%. Over the 6 month observation period measurement of whole saliva and parotid saliva flow showed no significant difference between the groups. Quality of life assessment again did not show a difference between the groups. Five patients discontinued tablets due to side effects, 2 taking pilocarpine due to sweating and 3 taking placebo because of nausea.

**Conclusions** Pilocarpine given adjuvantly during radiotherapy neither improves salivary function nor gives symptomatic relief.

### O40 OVERCOMING RADIORESISTANCE USING ULTRA LOW DOSES

S. Short and M.C. Joiner, Gray Laboratory Research Trust, Mount Vernon Hospital, Northwood, Middx HA6 2JR.

The phenomenon of low dose hyper-radiosensitivity (HRS) has been demonstrated in a number of human tumour cell lines and in some tumours and normal tissues *in vivo*. It produces increased cell kill per unit dose below one gray. The corresponding increase in radioresistance at higher doses is thought to be related to induced DNA repair following radiation which is only triggered above a certain dose. Previous work has suggested that these effects may be more apparent in more radioresistant cell lines (compared to both sensitive cell lines and normal tissues). If this is the case the use of very low doses per fraction may offer a real therapeutic gain over conventional treatment in some tumours by exploiting their low dose hypersensitivity. We have looked for HRS in radioresistant human cell lines using a microscopic cell analyser to accurately determine cell survival at very low doses (down to 0.05 Gy) *in vitro*. We have confirmed the effect reported previously in the human bladder carcinoma line RT112 and have now demonstrated HRS in a newly tested glioblastoma line, T98G. A second radioresistant glioma cell line, U-373 which we have also newly tested did not show the effect; this now indicates a less straightforward relationship between radioresistance to conventional doses and low dose hypersensitivity/induced radioresistance. These data nevertheless suggest that for some tumours a workable therapeutic regime could be based on 'ultra-fractionation' i.e. doses <1 Gy per fraction using multiple fractions per day, which may overcome their resistance to conventional therapeutic regimes.

### O41 NORMAL TISSUE RADIOSENSITIVITY: THE ROLE OF DNA DAMAGE ASSAYS

C.J. Orton[1], A.E. Kiltie[2], A.Ryan[2], C.M.L. West[2], J.H. Hendry[2], R.D. Hunter[1], S.E.Davidson[1], [1]Dept. Clinical Oncology, Christie Hospital, [2]Dept. of Experimental Radiation Oncology, Christie CRC Research Centre, Manchester M20 4BX

Introduction: Several promising correlations have been reported between fibroblast radiosensitivity *in vitro* as measured by a clonogenic assay and the severity of late normal tissue reactions. There is increasing interest in more rapid tests of radiosensitivity, in particular those that measure DNA damage. There have been few reports studying the relationship between the different methods available. In this work a comparison has been made of fibroblast radiosensitivity measured using a clonogenic assay and three gel electrophoresis techniques; pulsed field, graded voltage and constant voltage gel electrophoresis. Materials: Eleven fibroblast strains were studied comprising two radiosensitive human strains and nine strains established from vaginal biopsies from patients with carcinomas of the cervix prior to a radical course of radiotherapy. Methods: Cells were labelled with tritiated thymidine for 72 h and grown to confluence. After 10 days they were irradiated at a dose rate of 1.87 Gy $min^{-1}$ to doses between 30 and 180 Gy. Residual DNA damage at 24 h was measured as the fraction of activity released (FAR) into the gels. Results: For all three methods there were highly significant correlations between cell surviving fractions at 2 Gy and the slope of FAR, $r>0.88$, $p<0.01$. The correlations among the three gel electrophoresis methods were also highly significant, $r>0.89$, $p<0.01$. Conclusions: The future of DNA damage assays in predicting normal tissue radiosensitivity appears to be promising.

### O42 RADIATION INDUCED CELL-CYCLE DELAYS IN ASYNCHRONOUS POPULATIONS OF NORMAL FIBROBLASTS ARE RELATED TO RADIOSENSITIVITY.

C.J.Orton[1], J.Berry[2], [1]Dept. Clinical Oncology, Christie Hospital, [2]Dept. Medical Physics and Instrumentation, Christie CRC Research Centre, Manchester M20 4BX

Introduction: It is possible to study irradiation induced cell-cycle delays in asynchronous populations with bivariate flow cytometry. Materials: Twelve normal human fibroblast cell strains were studied. Methods: Confluent cells were irradiated and then plated out at low cell density for 72 hrs, before performing cell-cycle analysis. Results: $G_2$ block following irradiation is measured by the emptying of the original $G_2$ compartment; in the most radioresistant cell strains maximum block occurs at 2 Gy whereas in the radiosensitive strains this does not occur even after 4 Gy. The proportion of cells which cycle following irradiation correlates with the cell surviving fractions at 2 Gy, $SF_2$, $r=-0.86$, $p=0.026$, at 2 Gy. The accumulation of cells in the first $G_1$ compartment following irradiation is greater in radioresistant cells than in radiosensitive cells; at 4 Gy this relationship reaches significance when correlated with $SF_2$, $r=0.81$, $p=0.05$.The percentage of cells entering the $G_2$ compartment in the first cycle is the same irrespective of whether the cells are irradiated or not. In cycling cells $G_2/M$ delay measured by the ratio of the percentage of cells in $G_2$ in the first cycle, to the percentage of cells in $G_1$ in the second cycle, correlates with $SF_2$, $r=-0.89$, $p=0.03$ at 2 and 4 Gy. Conclusions: Irradiation induced cell-cycle delays correlate with radiosensitivity.

### O43 CELL CYCLE ARREST, APOPTOSIS AND RESPONSE TO IRRADIATION AND ANTIBODY IN LYMPHOMA CELLS.

**T.M.Illidge, M.S.Cragg, M.J.Glennie; Lymphoma Research Laboratory, University of Southampton. S016 6YD. UK**

The cellular response to DNA damage following irradiation of p53 mutant lymphoma cell lines involves a cell cycle arrest at the $G_2/M$ check-point; prior to the appearance of apoptosis and irradiation can influence antibody induced apoptosis. All 10 lymphoma cell lines studied demonstrated upto a four fold peak increase in the number of cells in $G_2/M$ which occurred 18 to 24 hours after irradiation and prior to apoptosis. The kinetics of apoptotic cell death varied considerably and correlated with the rapidity of exit from the irradiation induced $G_2/M$ arrest. The loss of function of wild-type p53 and the time period elapsed in $G_2/M$ arrest following irradiation, appear to predict radiosensitivity. Our results taken together strongly suggest that apoptosis is involved in the increased radiosensitivity of cells which exit rapidly from the $G_2/M$ checkpoint. Lymphoma cells which stay longer in $G_2/M$ were more resistant to apoptosis than cells which exited faster. Abrogation of the $G_2/M$ arrest by Caffeine produces increased radiosensitisation of p53 mutant radioresistant Burkitt's lymphoma cell lines by producing increased irradiation induced apoptosis.

DNA damage by increasing doses of irradiation, appears to inhibit the early onset of anti-μ (anti-B cell receptor) but not anti-CD20 induced apoptotic cell death. Anti-CD20 antibodies when combined with irradiation demonstrate an additive effect in some cell lines. This finding of an antagonistic effect rather than an additive effect with irradiation and anti-μ antibodies was unexpected and suggests that the signals for $G_2M$ arrest initiated by irradiation induced DNA damage can inhibit or over-ride the programmed cell death initiated by Ceramide following stimulation of the B-cell receptor.

### O44 IRRADIATION MODIFIES KERATINOCYTE MIGRATION IN RESPONSE TO GROWTH FACTORS AND CYTOKINES *IN VITRO*

S.Pledge[1], H.Robson[1], E.Anderson[1] and J.Hendry[2]. [1]Clinical Research Dept, Christie Hosp, [2]Exp.Rad.Biology, Paterson Inst.Cancer Res, Manchester, UK. (S.P.now YCRC Ac.Dept Clin Onc, Weston Park Hospital, Sheffield)

Many radiotherapy patients experience acute reactions in epithelial tissues which can result in treatment delay or dose reduction. We have investigated methods of ameliorating these acute reactions using cytokines and growth factors to radioprotect the normal tissues, maximise cellular repopulation and promote keratinocyte migration into denuded areas.

Cultured human keratinocytes were used for experiments at passages 2-4. The effects of epidermal growth factor (EGF), basic fibroblast growth factor (bFGF), keratinocyte growth factor (KGF) and transforming growth factor β3 (TGFβ3) on keratinocyte proliferation and migration under normal conditions and after irradiation were determined. Proliferation was measured using a 96 well plate colorimetric assay of cell density and capacity for migration was assayed using a phagokinetic track method [Albrecht-Buehler G., *Cell* (1994), **11**, 395] with and without prior irradiation (5Gy γ radiation). TGFβ3 was added before irradiation of the cells whereas the other factors were added after. The effects of the cytokines on cell cycle distribution of the keratinocytes and expression of the EGF receptor with and without irradiation were determined using flow cytometry and ligand binding analysis.

Both EGF and KGF stimulated proliferation whereas TGFβ3 inhibited it whether the cells were irradiated or not. The results of the migration experiments showed that EGF was the only factor that could significantly enhance migration. Irradiation on it's own slightly reduced migration, however, when administered before addition of the factor, irradiation significantly enhanced the motogenic effects of EGF on the keratinocytes. This may be related to induction of EGFR expression by irradiation as has been reported previously [Peter R.U. *et al*, *Radiation Res* (1993) **136**, 65]. Alternatively, the keratinocytes may be able to migrate more easily when they are arrested in $G_2$ as after irradiation.

### O45

**IS HUMAN TUMOUR BLOOD FLOW INFLUENCED BY CARBOGEN AND NICOTINAMIDE?**

[1]MEB Powell, [2]SA Hill, [1]MI Saunders, [1]PJ Hoskin, [2]DJ Chaplin, [1]MCRW, [2]Tumour Microcirculation Group, CRC Gray Lab, Mount Vernon Hospital, Northwood, UK.

**Aim** Hypoxia is recognised as a mechanism of treatment resistance to many anticancer therapies. Modification of the tumour microenvironment by increasing perfusion and oxygenation of tumours may improve on the success of these treatments. To address this clinical trials are underway to evaluate nicotinamide and carbogen (95%$O_2$, 5%$CO_2$) in combination with accelerated radiotherapy. Using laser Doppler probes to measure microregional tumour blood flow, this study examines the influence of nicotinamide and carbogen on tumour perfusion.

**Methods** Ten patients with advanced cancer were studied. Nicotinamide (80mg/kg) was given orally and 60 minutes later up to 6 probes were inserted into the tumour. One hour of readings were taken prior to 10 minutes of carbogen and 10 further minutes breathing room air. Results were compared with a similar group of 8 control patients not given nicotinamide but who breathed carbogen.

**Results** In 44 microregions analysed, 33 (75%) showed perfusion fluctuations of ≥50%, and 20 (44%) by ≥100%. This compared with the control group in whom 62% and 27% of microregions varied by ≥50% and ≥100% respectively. Perfusion increases outweighed decreases by 1.3 with nicotinamide and 1.2 in the controls. After 5 and 10 minutes of carbogen tumour perfusion increased by 17% ($p<0.004$) and 22% ($p<0.001$) with nicotinamide and 0% and 1% in the control group.

**Conclusions** Pre-treatment with nicotinamide made little difference to random blood flow fluctuations seen in controls. However, with nicotinamide plus carbogen, tumour perfusion more reliably increased compared with carbogen alone. This may have important therapeutic implications by improving response to treatment and allowing better delivery of systemic agents.

### O46

**FUNCTIONAL IDENTIFICATION AND PRELIMINARY BIOLOGY OF A NOVEL OVARIAN CANCER TUMOUR SUPPRESSOR GENE (TSG) ON CHROMOSOME 11q24.**

H.Gabra*[1], J. E. V. Watson[1], K. J. Taylor[1], G. J. Rabiasz[1], A. A. Ritchie[1], C.M. Steel[2], J. F. Smyth[1] and D. J. Porteous[2], [1]ICRF Medical Oncology Unit, [2]MRC Human Genetics Unit, Western General Hospital, Edinburgh.

We have previously reported that LOH of an 8.5 Mb region on 11q23-q24, is associated with advanced FIGO stage and poor survival in ovarian cancer(Gabra et al *Cancer Res* **56**, 950-954, 1996).
We used a mouse A9 somatic cell hybrid (556.1.5) to transfer a normal human chr 11 by microcell mediated chromosome transfer into OVCAR3 (a poorly differentiated ovarian adenocarcinoma cell line with chr 11 rearrangement). Transfer of whole human chr11 was confirmed by chromosome paint, primed in-situ hybridisation (PRINS), and microsatellite analysis. Fourteen microcell hybrid clones (MHCs) were derived from 5 experiments in which chr 11 was transferred into a Hygromycin$^R$-tagged OVCAR3 subline. The immortalised parental phenotype was retained in all chr 11 MHCs. Significant *in-vitro* and *in-vivo* growth inhibition was noted for chr11 MHCs compared with control parent Hyg$^R$ OVCAR3 and control MHCs with null chromosome transfers. Flow cytometric analysis for cell cycle distribution and Annexin-V assay for apoptosis suggested that the observed inhibition was not mediated by these processes.
Matrigel invasion assays demonstrated significant reduction in invasiveness of the chr11 OVCAR3 MHCs that retained distal 11q compared with MHCs that had lost distal 11q and OVCAR3 controls. Further analysis demonstrated that this was associated with inhibition of attachment and spreading to laminin coated plastic and inhibition of cell migration associated with a collagen IV or fibronectin haptotactic signal. Integration of microsatellite mapping data from the MHCs with that of LOH from the patients has reduced the size of the interval housing the TSG to 4.5 Mb.
The phenotypes derived from the above techniques are being used to isolate this novel and prognostically important tumour suppressor gene by cDNA difference cloning methodology.

### O47

**OVARIAN CANCER: IMPROVEMENT IN SURVIVAL IN SCOTLAND WITH PLATINUM CHEMOTHERAPY.**

E J Junor on behalf of the Scottish Cancer Therapy Network.

**Object** To determine changes in management and survival of patients with ovarian cancer in Scotland Between 1987 and 1993.

**Methods** Retrospective case record review of all cases of ovarian cancer registered to the Scottish Cancer Registration Scheme in 1987 and 1993. Data extracted with regard to demography, treatment and outcome. Cox's proportional hazard model was used to elicit independent treatment variables while adjusting for prognostic variables.

**Results** 1040 patients registered of whom 931 eligible for study. The two year survival for all patients in 1987 and 1993 were 38.0% and 42.8% respectively. For patients over 64 years, 2 year survival was 23.4% in 1987 and 33.6% in 1993. Age, stage, histological group, degree of differentiation and ascites were independent prognostic variables.
Compared to 1987 there has been a marked increase in the use of platinum in patients 64+ in 1993. After adjusting for the five 'biological' prognostic variables, speciality of the first hospital contact, specialty of operator, residual disease and attendance at a multidisciplinary clinic, there remained a statistically significant ($P <.006$) difference in survival for patients 64+ years between 1987 and 1993. This difference was entirely due to platinum chemotherapy.

**Conclusion** All the improvement in survival in patients over 64 years between 1987 and 1993 can be accounted for by the increased prescription of platinum chemotherapy.

### O48

**ASSESSMENT OF TUMOUR REGRESSION RATES DURING RT FOR CA CERVIX USING SERIAL MRI**

L T Tan, C S Romaniuk, J Brunt, B Jones
Clatterbridge Centre for Oncology, Wirral.

The potential importance of tumour regression rates in determining the optimum timing of brachytherapy in relation to external beam RT in order to achieve maximum tumour cell kill has been explored in mathematical modelling exercises. However, before it is possible to evaluate the effect of tumour regression rates on tumour control in clinical practice, a reliable method of estimating the tumour regression rate is required. A prospective study has therefore been undertaken to assess the feasibility of estimating the regression rates of cervical carcinomas during radiotherapy using serial MRI.

Eleven patients with FIGO Stage IB-IIIB cervical carcinoma have been included in the study. Each patient underwent a routine staging MR examination of the abdomen and pelvis prior to commencing RT followed by serial MR scans of the pelvis at weekly intervals during RT. The staging and serial pelvic examinations included sagittal and oblique axial T2-weighted fast spin echo pulse sequences using long repetition times (range 2200-3500 msec) and echo times (150 msec) at 0.5 Tesla (Philips Gyroscan T5-II). Images were acquired using 6 mm contiguous slices, 179 x 256 matrix, 350 mm field of view and 6 signal averages. Constant scan parameters were used in all examinations performed on an individual patient. Tumour volumes were estimated using 2 methods by a radiologist and an oncologist:

1. Planimetry The tumour mass was manually outlined on serial images using the manufacturer's computer console and standard measurement software.
2. Stereology Tumour volumes were estimated on the same images as for planimetry using the unbiased stereological method of manual point counting.

Tumour volume regression rates were determined from a semi-logarithmic plot of sequential tumour volumes against time using linear regression analysis.

Initial tumour volumes ranged from 6 $cm^3$ to 200 $cm^3$. Tumour regression occurred within days of commencing radiotherapy and followed a negative exponential function with time. Estimated tumour volume regression rates ranged from 2% per day to 20% per day. There was no correlation between the estimated tumour regression rates and the initial tumour volumes. A minimum of 4 scans was required to estimate the tumour regression rate with reasonable precision. Similar regression rates were obtained using both planimetry and stereology. There was no significant intra-observer or inter-observer variability in the estimated regression rates.

The study has shown that it is possible to estimate the rate of tumour regression during RT for carcinoma of the cervix with reasonable precision using serial MRI.

### O49 TUMOUR ANTIGEN EXPRESSION IN OVARIAN TISSUE

AM Gillespie[1], S Rodgers[1], AP Wilson[2], AK Murray[1], RC Rees[3], J Tidy[4] and RE Coleman[1]. [1]YCRC Inst. Cancer Studies & Dept. Clinical Oncology, University of Sheffield, Sheffield, [2]Oncology Research Laboratory, Derby City Gen. Hospital, Derby, [3]Dept. Life Sciences, Nottingham Trent University, Nottingham, [4]Dept. Obstetrics & Gynaecology, University of Sheffield, Sheffield, UK.

Ovarian cancer accounts for 6% of female cancer deaths. In the near future novel treatment modalities may contribute to an improved prognosis for women diagnosed with this disease.
Antigen Specific Immunotherapy with MAGE peptide products may be one such therapy. It is known that MAGE 1-4, 6 & 12 genes are expressed in some human cancers. MAGE 1 & 3 are targets for specific immunotherapy as they encode peptide antigens -" tumour antigens "- which are presented in association with HLA class 1 molecules and are recognised by cytotoxic T lymphocytes.
To determine if ovarian cancer patients would be suitable for MAGE-peptide vaccine based immunotherapy, the frequency of expression of the MAGE 1-4 genes in ovarian tumours was assessed using Reverse Transcription Polymerase Chain Reaction (RT-PCR) and product verification with digoxigenin-labelled oligonucleotide probes specific for each MAGE gene. In addition the frequency of expression of more recently discovered tumour antigens (BAGE, GAGE-1,-2 and GAGE-3,-6) was established using RT-PCR and ethidium bromide staining.
To date in this study 15/28 ovarian cancer specimens have expressed MAGE 1, including 10/13 serous adenocarcinomas. In addition 13/21 benign pathological ovarian lesions studied also expressed MAGE 1, including simple cysts, cystadenomas and adenofibromas. No MAGE expression was detected in 10 normal ovarian specimens. Expression of other tumour antigens was rare.
This study indicates that serous cystadenocarcinomas may be suitable candidate tumours for MAGE peptide immunotherapy, whilst previous assumptions regarding the role of MAGE genes in carcinogenesis is questioned by our finding of expression in both benign and malignant tissue.

### O50 A RANDOMISED TRIAL OF DOSE INTENSITY IN ADVANCED EPITHELIAL OVARIAN CANCER (EOC).

P.N. Mainwaring*, R.P. A'Hern, V. Macfarlane, M. Slevin, P. Harper, R. Osborne, M. Quigley, A. Jones, J. Mansi, P. Blake, E. Wiltshaw, M.E. Gore for the London Gynaecological Oncology Group

There have been 9 randomised trials of platinum dose intensity in advanced EOC. Two of the smaller trials have suggested a benefit but all of the previous studies have used platinum in combination with other drugs. Two hundred and forty patients with advanced EOC were randomised to receive single agent carboplatin, dosed according to the Calvert formula, either at a standard AUC 6 for 6 cycles or at an AUC of 12 for 4 cycles, in both arms of the study the treatment cycle was 28 days. The median total dose received was 3,580 mg (700 - 5,640) and 4,311 mg (0 - 7,490) for the standard and higher dose arms respectively, representing a 20% percent increase in received total dose. Dose modifications were introduced if patients experienced prolonged platelet and/or neutrophil myelosuppression or significant falls in $^{51}$Cr-EDTA clearance. Haematological toxicity was assessed by full blood counts prior to each cycle and on day 14 in patients receiving the higher dose treatment. $^{51}$Cr-EDTA clearance was performed prior to each course of therapy for patients receiving carboplatin AUC12 and prior to courses 1 and 4 for those treated in the standard dose arm. 122 patients had measurable disease and 35 had evaluable disease only. There were no significant differences in response rates between the two patient groups; AUC6 64% (32%CR) and AUC12 55% (32%CR). There was no significant difference in progression-free or overall survival between the two treatment arms. Median progression-free and overall survival were 12 and 29 months (AUC6) and 11 and 36 months (AUC12) respectively. Dose reductions were made in 3% (3/117) and 26% (27/109) of patients receiving carboplatin AUC6 and AUC12, respectively. Significantly more infections (p = 0.003), diarrhoea (p = 0.07) and alopecia (p = 0.02) occurred in the carboplatin AUC12 arm. We have not shown a survival benefit for increasing the received total dose of carboplatin by 20%.

### O51 A PHASE II STUDY OF EPIRUBICIN, CISPLATIN AND PROLONGED VENOUS INFUSIONAL 5-FU (ECF) IN RELAPSED EPITHELIAL OVARIAN CANCER (EOC).

FY Ahmed, PN Mainwaring, DM King, RP A'Hern, M Ramsden, B McLaren, J Mansi, P Harper, M Slevin, H Thomas, ME Gore for The LGOG

The probability of response to chemotherapy following relapse in patients with EOC is related to the treatment- free interval, for instance response rates range between 17-26% for patients (pts) rechallenged with platinum-based chemotherapy within 12 months (Gore et al 1990, Markman et al 1991). Results with newer phase II agents such as paclitaxel, topotecan, altretamine, and gemcitabine, are not substantially better than this. ECF has been evaluated in a number of solid tumours, most notably in gastric and breast cancer. We have assessed the efficacy of this regimen in relapsed EOC. Eligibility criteria were; performance status (PS) 0-2, relapse following one prior platinum-based chemotherapy regimen or not more than two prior regimens and a treatment-free interval of less than 12 months. 55 pts were entered and their characteristics were: median age 51 years (range 68-21); PS 0 in 13 pts; 1 in 37 pts, and 2 in 5 pts. Patients were stratified according to previous treatment as follows: group 1 relapse, less than 4 months from last treatment, group 2 relapse 4-12 months from last treatment. The treatment consisted of continuous intravenous infusional 5-fluorouracil (5FU) 200 mg/m$^2$ per day via a Hickman line and ambulatory pump. In addition patients were given 3-weekly boluses of cisplatin 60 mg/m$^2$ iv and epirubicin 50 mg/m$^2$ iv for a maximum of 18 weeks (6 cycles). There were 8 complete remissions (CR) and 15 partial remissions (PR) with a median duration of response of 7+ months (range 2+ to 18+ months). The overall response rate was 23/55 (42%); 10/35 (29%) group 1, 13/20 (65%) group 2. The treatment was well tolerated in the majority of patients. ECF is a very active regimen in patients with EOC relapsing from platinum-based or multiple prior chemotherapy regimens.

### O52 THE G-CAT REGIMEN: PLATINUM, TAXANE, & ANTHRACYCLINE FOR ADVANCED OVARIAN CANCER.

M.E. Hill, R.K. Gregory, J. Moore, S.R.D. Johnston, P. Blake, J. Shephard, D. Barton, M.E. Gore. Gynaecology Oncology Unit, Royal Marsden NHS Trust, London, SW3 6JJ.

A recent meta-analysis has suggested that addition of an anthracycline to platinum-based chemotherapy may improve survival in advanced ovarian cancer. In GOG trial 111, cisplatin/paclitaxel appeared superior to cisplatin/cyclophosphamide. It is thus logical to investigate the feasibility of a platinum/anthracycline/taxane combination in this disease, both as a definitive therapy or prior to further intervention (e.g. interval debulking surgery, high dose chemotherapy etc.). G-CAT is one such regimen, and consists of doxorubicin (dox) 50mg/m$^2$ or epirubicin (epi) 60 mg/m$^2$ bolus, paclitaxel (tax) 175mg/m$^2$ over 3 hours and either cisplatin (cis) 75mg/m$^2$ or carboplatin (carbo) AUC 7, with G-CSF at the neutrophil nadir. 26 patients have been treated in a phase I/II study which has toxicity as the main endpoint. 8 patients received dox/tax/carbo, 8 dox/tax/cis and 10 epi/tax/carbo. Treatment was given 3-4 weekly and toxicity assessed according to CTC criteria. Median age was 49 (range 27-67). 13 patients had epithelial ovarian cancer (all ineligible for national trials) and 13 gynaecological carcinomas therapeutically similar to ovarian cancer (peritoneal, fallopian tube, mixed Mullerian). 134 cycles of chemotherapy have been given, with a median of 6 per patient (range 2-6). 69% of patients required at least one dose reduction. 19 patients experienced grade 3-4 neutropenia (8/8 with cis) and 11 grade 3-4 thrombocytopenia (10/18 with carbo). There were 6 grade 3/4 infections and no toxic deaths. Non-haematological toxicities were manageable, with the exception of lethargy which occurred in 75% of cis treated patients. Grade 1/2 cardiotoxicity as assessed by pre- and post-treatment left ventricular ejection fraction was observed in 5/13 patients who had received dox (results from epi treated patients will be presented). There was no grade 3/4 cardiac toxicity or clinically evident cardiac dysfunction. The response rate in 20 evaluable patients was 95% (12 CR, 7 PR). We conclude that G-CAT shows high activity and can be administered safely. Only very fit patients are suitable as the regimen is associated with considerable toxicity, but of the 3 combinations tested, epi/tax/carbo was the best tolerated.

### O53 A RANDOMISED TRIAL COMPARING ECF WITH FAMTX IN ADVANCED OESOPHAGO-GASTRIC CANCER - UPDATED 2 YEAR FOLLOW-UP.

Webb A[1], Cunningham D[1], Scarffe JH[2], Harper P[3], Norman A[1], Joffee JK[4], Hughes M[5], Mansi J[6], Findlay M[1], Hill A[1]. The Royal Marsden Hospital, Sutton, Surrey[1]. Christie Hospital, Manchester[2]. Guys Hospital, London[3], St James Hospital, Leeds[4]. Beatson, Glasgow[5]. St Georges Hospital, London[6], UK.

Aim: To evaluate a prospectively randomized study comparing ECF (epirubicin (50 mg/m$^2$), cisplatin (60 mg/m$^2$), protracted venous infusion 5-fluorouracil (200 mg/m2/day)] with the standard combination of 5-fluorouracil, adriamycin and methotrexate (FAMTX) in previously untreated patients with advanced oesophago-gastric cancer.

Methods: 274 patients with adenocarcinoma/undifferentiated carcinoma were randomized, and analyzed for survival, tumour response, toxicity, quality of life (QL) and cost analysis.

Results: Overall response rate was 46% in ECF and 21% in FAMTX (P=0.00003). Toxicity was tolerable and there were only 3 toxic deaths. FAMTX regimen caused more haematological toxicity and serious infections but ECF caused more emesis and alopecia. The median survival was 8.6 months in ECF and 6.0 months in FAMTX (P=0.001). For ECF, 1 and 2 year survivals were 36.9%, 12.9% and for FAMTX 21.5%, 5.5% respectively with 92% of patients dead. The median failure free survival was 7.3 months in ECF and 3.2 months in FAMTX (P<0.0001). The global QL scores were better for ECF at 24 weeks but the remaining QL data showed no differences between either arm of the study. Hospital based cost analysis on a subset of patients was similar for each arm and translated in to an incremental cost of £750 per life year gained.

Conclusions: The ECF regimen results in a survival and response advantage, tolerable toxicity, better QL and cost effectiveness when compared to FAMTX chemotherapy. This regimen should now be considered the standard treatment for advanced oesophago-gastric cancer.

### O54 TELOMERASE ACTIVATION IS AN EARLY FEATURE OF COLORECTAL NEOPLASIA

S. O Odogwu, I. A Fraser & A.G Morris, Dept. of Surgery, Walsgrave Hospital, Coventry & Dept. of Biological sciences, Warwick University

Background: Telomerase is an enzyme which has been identified in germ cells and several tumours. It is not expressed by normal somatic tissues.Telomerase expression enables cells to divide indefinitely and renders them immortal.This immortalization may be an important step in the malignant process.

Aims: To determine the incidence of telomerase expression in colorectal cancers and adenomatous polyps and to identify the stage at which the enzyme is first activated.We also analyzed the degree of telomerase activity in polyps and correlated this to the degree of dysplasia.

Methods: Telomerase activity was detected using the Telomeric Repeat Amplification Protocol (TRAP assay).This is a highly sensitive Polymerase Chain Reaction (PCR) based technique.PCR products were resolved by gel electrophoresis,autoradiographed and analyzed by densitometry.

Results: 48 out of 50 (96%) cancers were found to be positive for telomerase.These ranged from Duke's A lesions to metastatic tumours. 50 corresponding samples of normal mucosa were negative

5 out of 35 (14%) polyps were found to be telomerase positive. All 5 were villous adenomas.The degree of activity increases with degree of dysplasia.

Conclusions:Telomerase is expressed early in the malignant process in colorectal cancer.Enzyme activation appears to occur at the dysplastic polyp stage.It is very tumour specific and no significant activity was detected in any of the normal samples analyzed. In the future, telomerase may be a useful marker for early diagnosis or follow up of malignant disease. It is also a potential target for drug therapy.

### O55 IS SMALL BOWEL IMAGING USEFUL WHEN PLANNING PRE-OPERATIVE RADIOTHERAPY FOR RECTAL CANCER?

M Collinson, A Crellin, S Edwards, J Campbell, D Sebag-Montefiore. Yorkshire Centre for Cancer Treatment, Cookridge Hospital, Leeds.

**Introduction.** The small bowel is the normal tissue responsible for a significant proportion of the acute and late morbidity of pelvic radiotherapy. Pre-operative radiotherapy for locally advanced rectal cancer has used either parallel opposed techniques [as in the MRC CR02 trial] or planned volumes. This study quantifies the small bowel volume [SBV] using both techniques.

**Methods.** Twenty patients with rectal cancer who were planned to receive pre-operative radiotherapy were studied. Patients were given 300mls of Baritop 100 and 20mls of Gastrograffin orally 45 to 60 minutes prior to simulation. Radiotherapy fields were as follows:- superior border_ 5 cm above tumour or S2/3 junction [whichever was more superior], inferior border_5 cm below the tumour, lateral borders_ 3 cm lateral to tumour, posterior border_ the whole thickness of sacrum at level of the tumour, anterior border_3 cm anterior to tumour or anterior rectal wall [whichever was the more anterior]. The SBVs were calculated using the method of Gallagher et al [1] for both a two field technique and a planned volume.

**Results.** The median planning target volume was 1896 cm3 [range 998-4520 cm3]. The median SBV was 5 cm3 [range 0 to 1103 cm3] with a planned technique and 49.8 cm3 [range 0 to 1239 cm3] using a two field technique. Ten patients [50%] with a planned technique and five patients [25%] with the two field technique had no small bowel in the treatment volume.

**Conclusions.** This study demonstrates that the use of planned volumes allowed the complete exclusion of small bowel from the treatment volume in ten patients, compared with five patients using a two field technique. The four patients with an SBV of > 100 cm3 could not be predicted on the basis of the either the size or position of the radiotherapy volume borders. Small bowel imaging is a simple technique and may allow a reduction in the treated SBV in a minority of patients by the use of shielding techniques.

**References.**

[1] Gallagher MJ, Brereton HD, Rostock RA, Zero JM, Zekoski DA, Poyss LF, Richter MP, Kligerman MM. A prospective study of treatment techniques to minimize the volume of pelvic small bowel with reduction of acute and late effects associated with pelvic radiation. Int.J. Radiat. Oncol. Biol.Phys. 12: 1565-1573, 1986.

### O56 RADIOTHERAPY(RT) VS RADIOTHERAPY + CHEMOTHERAPY(CT) AS ADJUVANT THERAPY FOR RECTAL CANCER : PRELIMINARY ANALYSIS OF PELVIC TOXICITY IN QUASAR

**Roger James and Pat Price**
**On behalf of the QUASAR steering committee**

**Objectives:** Adjuvant RT reduces the risk of local recurrence in operable rectal cancer. Adjuvant CT may improve survival in colorectal cancer. This study investigates whether the regimen of pelvic RT with concomitant adjuvant CT (CRT) produces unacceptable acute toxicity.

**Methods:** Patients (pts) scheduled for post-operative pelvic RT and randomised to the uncertain arm of QUASAR (5FU/high or low dose folinic acid/± Levamisole) or no CT were studied. Dose and schedule modifications were recommended within the protocol: (i)5FU 20% dose reduction once weekly (300 mg/m$^2$ wks 1-5 of therapy) (ii) pelvic RT ≤1.8 Gy per fraction (#). Acute pelvic toxicity was recorded & graded retrospectively from a questionnaire sent to clinicians (Grade I-IV CTC classification).

**Result:** Data were requested on all uncertain indication patients scheduled for post-operative RT between 4.94 & 10.96. 74 questionnaires were returned, 64 containing invaluable information (34 RT, 30 CRT). Pt age & sex were matched in both groups (median age 64 yrs; range 30-76 yrs, 31% female, 69% male), as were total RT dose, number of # & RT duration. The dose per # was higher in the RT arm (median 2.0 vs. 1.8, Mann Whitney p=0.002). Commonest RT schedules used were: 45 Gy/25 # (31%), 45 Gy/20 # (23%), 50.4 Gy/28 # (14%), 50 Gy/25 # (12%), 40 Gy/20 # (11%). No pt received pre-operative RT. Overall 44% reported pelvic (gastrointestinal and genitourinary) toxicity including 40% who developed diarrhoea, predominantly Grade II. Grade IV bowel toxicity occurred in 2 patients (both CRT). Pelvic toxicity was significantly more frequent in the CRT group (62% vs.29%)p=0.014. This toxicity did not correlate with pts' age, dose/#, number of # nor RT duration.

**Conclusion:** This preliminary analysis confirms that CT combined with RT produces more pelvic toxicity than RT alone. Pelvic CRT should be approached with caution. The dose modifications recommended in the QUASAR study appear reasonable. Pre-operative pelvic RT may be the preferred option when adjuvant CT is to be considered. A full quality of life study within QUASAR is ongoing.

### O57

**CHEMORADIOTHERAPY FOR RECTAL CANCER -HIGH COMPLIANCE, LOW TOXICITY AND EARLY RESULTS.** D. Sebag-Montefiore[1], R Glynne-Jones[2]; Cookridge Hospital, Leeds[1], Mount Vernon Hospital, Northwood[2].

**Introduction** Concurrent chemo-radiotherapy (CRT) in rectal cancer has been reported to result in high response rates although concerns remain over radiotherapy compliance and acute toxicity. The initial results using the same CRT protocol by two clinical oncologists in two centres is reported.
**Methods** 50 consecutive patients with rectal cancer have received CRT. Group 1:-CRT alone for 10 patients (6 mobile, 2 tethered,2 fixed) with a median age of 82 (range 75-92). Group 2:- pre-operative adjuvant CRT for 36 patients (22 fixed,10 tethered,4 bulky mobile) and Group 3 :-CRT for local recurrence for 6 patients. Radiotherapy - 3/4 field plan (45 Gy in 25 fractions). Chemotherapy - folinic acid 20mg/m2 i/v bolus followed by 5 Fluorouracil 350mg/m2 as a 30-60 minute infusion on days 1-5 and 29-33. Patients in group 2 were considered for resection 4-6 weeks after completion of CRT. 7 patients in group 1 received a brachy-therapy boost.
**Results** Of the 44 patients evaluable for compliance, 40 (91%) received 45Gy without a break, 3 patients (7%) received the full dose with a rest of 3-7 days and 1 patient (2%) received a total dose of 43.2Gy. 3/44 (7%) patients experienced WHO grade 3 toxicity (1 nausea, 1 diarrhoea, 1 urinary) and there was no Grade 4/5 toxicity . Group 1:- Of the 9 patients evaluable for response, 5 patients achieved clinical complete response (CCR) and 4 patients clinical partial response (PCR). Group2:- Of 30 evaluable patients for compliance, 29 (97%) 45Gy without a break and 1 patient required a rest from treatment of 7 days. All 30 patients received the full planned doses of chemotherapy. WHO grade 3 toxicity was seen in 2 (6%) patients (1 urinary and 1 diarrhoea). To date, 26 patients have undergone resection and was considered curative in 21 (81%). Histo-pathology is available for 21 patients:- path CR-2 (9%), Dukes A-4 (19%), Dukes B-10 (48%), Dukes C-5(24%). Group3:- Of 4 evaluable patients,1 patient experienced WHO grade 3 nausea. 1 patient had CCR, 2 patients have had resections, 1 of whom had path CR.
**Conclusions** CRT using the above regimen is associated with high compliance and acceptable toxicity. The early results demonstrate a high response rate in group 1 and the histopathological outcome for group 2 patients (9% pathological CR and 19% Dukes A) are encouraging.

### O58

**PREOPERATIVE CHEMORADIOTHERAPY FOR RECTAL CANCER: PRELIMINARY RESULTS OF ASSESSMENT BY PET SCANNING.** **S. Beesley, L. Biassoni, M. Maisey and M. Leslie. Depts of Clinical Oncology and Clinical PET, St Thomas' Hospital, London, UK.**

**The results of treatment for fixed/tethered rectal tumours can be improved by pre-operative chemoradiotherapy. Tumour response assessed clinically or by conventional imaging studies can only be determined some weeks after the completion of treatment. We have investigated whether PET scanning using FDG will allow an early assessment of response to treatment. So far 9 patients have been studied with serial PET scans before, during and after treatment. Our initial studies demonstrate that the tumours are well visualised with PET and that marked reductions in FDG uptake are seen once chemoradiotherapy is commenced. If PET scanning can predict for tumour response to chemoradiotherapy at an early stage in treatment it will allow for greater confidence in the use of the neo-adjuvant approach which will be of relevance to the treatment of tumours at a number of different sites.**

### O59

**FOLLOW-UP OF PATIENTS WITH METASTATIC LIVER LESIONS TREATED WITH INTERSTITIAL LASER THERAPY**
A R Gillams, J Brookes, C Hare, S Bown,* I Taylor,+ J Ledermann++ and WR Lees. The Departments of Medical Imaging, Surgery+, Medical Oncology++ and The National Laser Centre*, The Middlesex Hospital, London, UK.
**Object:** To determine outcome for patients enrolled in the liver tumour laser programme. **Methods:** The study group comprised 55 patients (37M:18F, mean age 56y (31-80) who were treated with interstitial laser therapy (ILT). The majority of patients had primary colorectal carcinoma. Patients were usually referred for ILT when other treatment options had been exhausted. Fourteen patients (25%) had had prior liver resection for metastases, 48 (87%) had received systemic (5 Flouracil and folinic acid) or intra-arterial chemotherapy at some stage, 36 prior to ILT, 10 concurrent and 2 post ILT. At the time of ILT mean lesion number was 2.7 (1-12) and mean lesion size 2.7cm (1-6cm). **Results:** Long term follow-up data was available in 44. Mean number of laser treatments was 2.2 (1-5). Mean length of survival from resection of the primary lesion was a minimum of 27.6 months (4-86). Mean length of follow-up from first detected liver metastasis was 18 months (3-47). Mean length of follow-up in the laser programme 10 months (2-41). One year survival was 87%. Reasons for treatment failure were the development of metastases elsewhere (n = 15), hepatic recurrence or new hepatic metastases (n = 21). Eight patients continue in the laser programme. Up-to-date survival figures will be presented.
**Conclusion:** An aggressive, multi-disciplinary approach to patients with limited liver metastases (<5 in number, <5cm diameter) is warranted in terms of improved survival.

### O60

MATURE DATA FROM A LARGE RANDOMISED TRIAL OF 'TOMUDEX' V 5-FLUOROURACIL PLUS FOLINIC ACID (MACHOVER REGIMEN) IN ADVANCED COLORECTAL CANCER. D.Cunningham,[1] D.Kerr,[1] P.Woll,[1] H.Anderson,[1] J.McAleer,[1] P.Harper,[1] F.Smith,[2] N.Minton.[2]

[1]'Tomudex' Study Group, [2]Zeneca Pharmaceuticals, Macclesfield

We report mature data (minimum follow up 9 months) from a randomised, open trial comparing the direct and specific thymidylate synthase inhibitor 'Tomudex' (raltitrexed, Zeneca) 3mg $m^{-2}$ iv over 15 min once every 3 weeks (n=247) with 5-FU 400mg $m^{-2}$ + FA 200mg $m^{-2}$ iv for 5 consecutive days every 4 weeks (n=248).

The median duration of treatment was 12.7 weeks (range 3-58) for 'Tomudex' and 16.9 weeks (range 4-60) for 5-FU + FA. Overall objective response was similar for both treatments (19% for 'Tomudex' and 18% for 5-FU + FA; odds ratio: 'Tomudex' : 5-FU + FA = 1.03; 95% CI 0.65 to 1.63, p = 0.90) as was median survival (10.7m for 'Tomudex' and 11.8m for 5-FU+FA; hazard ratio = 1.13; 95% CI 0.87 to 1.45, p = 0.36).

There was a statistically significantly lower incidence of WHO grade 3+4 mucositis (2% v 16%) and a lower incidence of leucopenia (6% v 13%) and diarrhoea (10% v 19%) on 'Tomudex' compared with 5-FU + FA and a statistically significantly higher incidence of asymptomatic, reversible, grade 3+4 elevations in hepatic transaminases (13% v 0%). 71% of patients on 'Tomudex' and 48% of those on 5-FU + FA received treatment without dose reduction or delay. Palliative benefits were seen for both treatments in terms of disease related symptoms and an improvement in performance status (38% of patients on 'Tomudex' and 31% on 5-FU + FA improved performance status relative to entry).

In conclusion, this mature data indicates that 'Tomudex' has similar activity to high dose 5-FU + FA (Machover regimen), with an acceptable safety profile and more convenient 3-weekly dosing schedule and reinforces the advantages of palliative chemotherapy for advanced colorectal cancer.

### O61

A PROSPECTIVE RANDOMISED TRIAL OF PROTRACTED VENOUS INFUSION(PVI) 5-FU WITH OR WITHOUT MITOMYCIN C (MMC) IN ADVANCED COLORECTAL CANCER. P Ross, A Norman, D Cunningham, A Webb, T Iveson, A Padhani, and A Massey. The Department of Medicine and The Gastrointestinal Unit, The Royal Marsden Hospital, London and Surrey, SM2 5PT.

Aims To compare PVI 5-FU with or without MMC for tumour response, survival, toxicity and quality of life (QL) in patients with previously untreated advanced colorectal cancer.
Methods 200 patients received PVI 5-FU (300mg/m$^2$/day for 24 weeks) with or without MMC (10mg/m$^2$ IV bolus 6 weekly for 4 courses; 7mg/m$^2$ from June 1995).
Results Overall response was 54% (95% confidence intervals [CI] 44.1-63.9%) with PVI 5-FU+MMC compared to 38% (CI 28.3-47.7%) with PVI 5-FU alone (p=0.024). PVI 5-FU+MMC caused more overall haematological toxicity but CTC grades 3/4 was increased only for thrombocytopenia (p=0.0006). Two patients treated with a cumulative MMC dose of 40 mg/m$^2$ developed haemolytic uraemic syndrome (HUS) warranting a reduction in cumulative MMC dose to 28mg/m$^2$. No HUS developed in patients treated with MMC to 28mg/m$^2$. Median failure free survival was 7.9 months with PVI 5-FU+MMC compared to 5.4 months with PVI 5-FU alone (p=0.033) with 31.9% and 17.7% respectively alive and failure free at 1 year. Median overall survival was 14 months with PVI 5-FU+MMC compared to 15 months with PVI 5-FU alone with 1 year survival of 51.7% compared to 57.2%. Global QL scores were better for PVI 5-FU+MMC
Conclusions PVI 5-FU+MMC results in improved response and failure free survival, tolerable toxicity and better QL when compared to PVI 5-FU alone. There is no irreversible toxicity with MMC at a cumulative dose of 28mg/m$^2$. This regimen is suitable for evaluation as adjuvant therapy in colorectal cancer.

### O62

RADIOTHERAPY IN THE TREATMENT OF ELDERLY PATIENTS WITH HIGH OR INTERMEDIATE GRADE NHL, J.P.Wylie, R.A.Cowan, Dept. Of Radiotherapy, Christie Hospital, Manchester M20 4BX...

The treatment of elderly patients with high or intermediate grade NHL remains difficult and controversial. 270 elderly patients treated between 1987-1992 with such a diagnosis were retrospectively reviewed. Eighty one, unfit for chemotherapy, received fractionated radiotherapy for apparently localised stage I or II disease. 25% were considered adequately staged. The pathology was reviewed in 77% of cases.
Median age was 78 years (range 70-87). Forty stage I and seventeen stage II patients had extra-nodal sites of disease. Nine had bulk disease and five B symptoms.
The radiation field included the primary site plus immediate adjacent nodes. After a median follow-up of 3.9 years the 5 year OS and DFS was 28% (CR 72%) and 21% respectively. Age (p=0.003), stage (p=0.004) and lactate dehydrogenase level (LDH) (p=0.006) were identified as independent risk factors for recurrence. These factors can define a group in which radiotherapy can produce acceptable survival rates.
These patients (age $\leq$80 years, stage I, LDH $\leq$500) had a 5 year DFS and overall survival of 56% and 62% respectively.
Using modern day prognostic factors it is possible to define a proportion of elderly patients, unfit for chemotherapy, who can derive acceptable relapse-free and survival rates from radiotherapy alone.

### O63

ALTERNATING ChlVPP/PABlOE IS BETTER THAN PABlOE ALONE AS INITIAL CHEMOTHERAPY FOR ADVANCED HODGKIN'S DISEASE: FIRST RESULTS OF A BNLI/CLG STUDY. B W Hancock[1], M H Cullen[2], G Vaughan Hudson[3], [1]YCRC Dept. of Clinical Oncology, Weston Park Hospital, Sheffield, [2]CRC Trials Unit, Queen Elizabeth Hospital, Birmingham, [3]BNLI, The Middlesex Hospital, London.

This British National Lymphoma Investigation (BNLI)/Central Lymphoma Group (CLG) trial commenced in October 1992 and was prematurely concluded in April 1996 after the first interim analysis. 682 patients (461 BNLI, 221 CLG) were randomised to either ChlVPP/PABlOE (chlorambucil, Velbe, procarbazine, prednisolone alternating with prednisolone, Adriamycin, bleomycin, Oncovin and etoposide) or PABlOE alone. 604 patients are so far evaluable for response and survival analysis. The patient characteristics were balanced between the two treatment arms. In the ChlVPP/PABlOE arm the complete remission and freedom from progression rates are significantly higher (75% vs 60% and 72% vs 52% at 2 years, respectively). At present there is no significant difference in the overall survival between the two arms. There was significantly more grade III, IV toxicity for myelosuppression and infection in the ChlVPP/PABlOE arm. ChlVPP/PABlOE will be a 'standard' therapy in the next United Kingdom Lymphoma Group randomised study in advanced Hodgkin's Disease.

### O64

**POST-TREATMENT REMISSION ASSESSMENT IN LYMPHOMA USING FDG-PET SCANNING,** **N.G. Mikhaeel[1], V. Ahern[1], S. Barrington[2], A.R. Timothy[1], Department of Clinical Oncology[1] and Clinical PET Centre[2], Guy's and St Thomas' Hospital, Lambeth Palace Road, London SE1 7EH, UK.**

**Although CT scanning is now the most widely used imaging technique in lymphomas, the information obtained from CT scans in the post-treatment setting is limited by the minimum volume of disease that can be detected and the inability to assess disease activity in residual abnormalities.**

**2-(Fluoro-18)-fluoro-2-deoxy-D-glucose (FDG) has been consistently shown to concentrate preferentially in metabolically active tissue including lymphoma. As part of an ongoing protocol to improve the accuracy of pretreatment staging and remission assessment for malignant lymphomas, we have been using FDG whole body Positron Emission Tomography (PET) scans in conjunction with CT scanning.**

**Fifty lymphoma patients have undergone FDG PET scanning for post-treatment remission assessment. Ten patients had residual activity on post-treatment PET scans but only 5 had residual abnormalities on CT with PET scans consistently showing more sites of involvement. Of the 10 PET scan-positive patients, 9 had persistent disease or relapsed later at sites of residual activity (ie. true positive PET). One patient had a negative biopsy of the mediastinum despite both abnormal PET & CT scans but the duration of follow-up on this patient is still short. Forty patients had normal post-treatment PET scans although CT scan showed residual abnormalities in 14 patients. No relapse in CT-positive sites has occured in these patients to date.**

**Our preliminary experience suggests that FDG PET scanning offers a highly sensitive method of assessing remission status following treatment of lymphoma.**

### O65 FIRST DEMONSTRATION OF ANTI-LYMPHOMA ACTIVITY OF BCL-2 ANTISENSE MOLECULE-G3139; RESULTS OF PHASE I/IIA CLINICAL TRIAL.

A Webb[1], D Cunningham[1], F Cotter[2], P Ross[1], J Walters[1], I Judson,[1] F Raynaud[1], P Clarke[1], Z E Dziewanowska[3], Royal Marsden Hospital[1] (RMH), UK, Institute of Child Health[2], UK, Genta Inc[3], US.

Introduction: It has been well known that T14/18 translocation in follicular lymphoma up-regulates BCL-2, leading to continued expression of BCL-2 protein. Upregulation of BCL-2 leads to extended survival of the cells and increased chemoresistance. Clinical trials demonstrated correlation between BCL-2 expression and poor clinical prognosis in an intermediate and high grade lymphomas. G3139 is an all-phosphorothioate 18mer oligonucleotides targeted to the first six codons of the BCL-2 mRNA. It has been shown to specifically down regulate BCL-2 *in vitro* and to have dose dependent activity in mice models of human lymphoma as well as other xenograft models of solid tumours.
Methods: The Lymphoma Unit at the RMH performed the first Phase I trial in all grades NHL pts who relapsed following several previous conventional chemotherapy regimens and who expressed BCL-2. Replicating preclinical xenograft model, the patients received G3139 as a continuous, subcutaneous 14 day infusion. The doses were escalated according to EORTC scheme and safety as well as efficacy measured using standard evaluation criteria.
Results: Until early February1997, 13 pts were entered in 6 dose escalation cohorts up to a dose of 147.2 mg/m$^2$/day. Based on excellent systemic tolerance the escalations were made in 100% increments. At the 6th dose level, reversible grade 3 thrombocytopenia was observed in 1 pt. Mild topical, infusion site irritation which was generally acceptable but two pts had more severe reversible reactions which were not dose dependent. Blood levels of two pts at 5th escalation level approximated concentration effective in *in vivo* models of lymphoma. In the first 9 pts, 4 pts demonstrated improvement in disease status as defined by clinical and.or laboratory parameters including decrease in BCL-2 protein. One of those 4 pts demonstrated minor tumour response. Another patient on the higher dose, who failed 4 prior therapies, with follicular grade II lymphoma, stage IVB, developed complete clinical and radiological response of 30+ week duration.
Conclusion: We conclude that antisense approach to BCL-2 constitutes a potentially important treatment modality in NHL, leading to responses in poor prognosis patients at doses causing low toxicity. The trial is continuing and the full update will be presented.

### O66 ESTABLISHING A REGIONAL BLOOD STEM CELL PHERESIS SERVICE: THE NORTH TRENT EXPERIENCE.

PC Lorigan*[1] and DA Jones[2]. [1]YCRC Dept of Clinical Oncology, Weston Park Hospital, Sheffield S10 2SJ, [2]National Blood Transfusion Service, Longley Lane, Sheffield S5 7JN.

In 1994, a regional blood stem cell pheresis service was established as a joint venture between Blood Transfusion Service (BTS) and Weston Park Hospital (WPH). The service is administered by BTS which also provides the nursing and technical expertise needed for the procedure and processing of the product. The facility is based at WPH which provides nursing back up. Medical expertise is provided by both centres.

162 patients have been referred for stem cell harvesting to date. These comprised non-Hodgkin's lymphoma (22.8%), relapsed Hodgkin's Disease (17.9%), multiple myeloma (11.7%), breast cancer (7.4%), and a mixture of acute leukaemia, Ewing's sarcoma, relapsed germ cell tumours, healthy donors, paediatric malignancies and others. 95% of cases were referred by the three centres providing Medical Oncology, Haemato-Oncology and Paediatric Oncology. Priming regimens were standardised, the majority of patients received cyclophosphamide 3g/m$^2$ and GCSF but a number of other regimens were also used. The decision to harvest was based on the peripheral CD34 positive cell count which was shown to correlate with the total yield (Lorigan et al. BJC 1996, 73, Suppl. XXVI, 235). 134 patients have undergone blood stem cell harvesting. The remaining patients were not harvested because of a low peripheral blood CD34 positive cell count, infection or disease progression. Patients had a mean of 2 harvests. The total CD34 positive cell yield was <1.0 x 10$^6$/kg in 19% of patients, between 1.0 and 1.9x10$^6$/kg in 20% and >2x10$^6$/kg in the remaining 61%. Factors which predicted poor mobilisation have been identified.

A regional blood stem cell pheresis service offers centralisation of expertise, economies of scale and the opportunity to standardise management of this heterogeneous group of patients.

### O67 TREATMENT OF PERIPHERAL BLOOD PROGENITOR (PBPC) HARVESTS BY TWO-STAGE IMMUNOMAGNETIC SELECTION: YIELDS COMPARABLE TO POSITIVE SELECTION ALONE.

P.W.M. Johnson[1], R. Coupe[2], D. Clarke[1], A. Lubenko[2], K. Short[1], T.J. Perren[1], P.J. Selby[1], S. MacLennan[2]. [1]ICRF Cancer Medicine Research Unit, St James's Hospital and [2]National Blood Service Yorkshire, Leeds.

*Purpose:* To determine the feasibility of sequential positive and negative selection to enrich haemopoietic progenitors and deplete tumour cells from PBPC's for rescue following high-dose chemotherapy.
*Methods:* Aliquots of 9 PBPC harvests (0.59-2.3 x 10$^{10}$ cells) were processed by immunomagnetic selection using antibodies (Ab's) to CD34 for enrichment of haemopoietic precursors, followed by depletion of tumour cells using a cocktail of Ab's to either 5 lymphoid or 3 epithelial antigens for lymphoma (5 pts) or breast cancer (4 pts) respectively. Separations were carried out using paramagnetic beads and a magnetic column, with removal of beads after the positive selection by competitive release. Numbers of CD34+ cells were measured by flow cytometry and CFU-GM enumerated in the apheresis product and at each stage of the procedure.
*Results:* The initial mean concentration of CD34+ cells was 1.73% (+/-0.81), increased to 90.8 % (+/-8.25) following enrichment and 92.6% (+/-7.05) after both stages. CFU-GM were enriched a mean 203-fold (+/-164) after enrichment and 241-fold (+/-132) in the final product. For the enrichment step the mean yield of CD34+ cells was 34.5% (+/-11.8), and for depletion 92.1%(+/-6.7). Overall the mean yield of CD34+ cells was 33.6% (+/-9.15). By projecting the numbers of CD34+ cells from the total harvest on 2 days apheresis, all 5 patients with lymphoma and 1 patient with breast cancer would have had sufficient numbers (>2 x 10$^6$/kg) for rescue following both stages. The proportion of CD34+ cells in the apheresis product was a good predictor of adequate numbers of cells remaining after processing, with a cut-off at 1%.
*Conclusion:* Two-stage selection of PBPC gives yields of early progenitors very similar to single stage enrichment, suggesting that this is a feasible method for in vitro treatment to remove tumour cells, provided the harvest contains at least 1% CD34+ cells.

### O68 STEM CELL FACTOR (r-metHuSCF) IN COMBINATION WITH FILGRASTIM (r-metHuG-CSF) ENHANCES PERIPHERAL BLOOD PROGENITOR CELL (PBPC) MOBILISATION FOLLOWING CHEMOTHERAPY: A RANDOMISED STUDY

**A. Weaver**[1], P.J. Woll[2], M. Lind[3], C.Gill[4], B. Jenkins[4], T.M. Dexter[1], N.G. Testa[1] and D. Crowther[1]. CRC Depts of Medical Oncology and Experimental Haematology- [1]Christie Hospital, Manchester, UK. [2]Nottingham City Hospital, UK. [3]Newcastle General Hospital, UK. and [4]Amgen Cambridge, UK.

It has previously been reported that SCF synergizes with filgrastim to enhance PBPC yields in patients with breast cancer undergoing cytokine-only mobilisation . We report here that SCF plus filgrastim following chemotherapy also enhances PBPC mobilisation compared with filgrastim alone. 48 chemotherapy-naive patients with FIGO stages 1c-IV epithelial ovarian cancer were randomised to receive either SCF and filgrastim or filgrastim alone following cyclophosphamide 3g/m$^2$. The dose of SCF was cohort dependent, with 12 patients in each cohort, 9 of whom received filgrastim (5μg/kg) plus SCF and the remaining 3 receiving filgrastim (5μg/kg) alone. The dose of SCF was 5,10,15, and 20μg/kg in cohorts 1, 2, 3 and 4 respectively. Growth factors were administered daily from day 3 post-chemotherapy until WBC was ≥4x10$^9$/L, when all patients underwent an apheresis, the product of which was divided into 4 aliquots, one aliquot being reinfused following each subsequent dose-intensive cycle of chemotherapy. We have demonstrated a statistically significant increase in colony-forming cells and CD34+ cells as the dose of SCF was increased. Likewise there was a significant enhancement in yields of CD34+ cells, CFU-GM, BFU-E and LTC-IC in those patients receiving the higher doses of SCF plus filgrastim compared with those receiving filgrastim alone. The median number of days (range) peripheral blood CD34+ cells, CFU-GM and BFU-E remained above specified threshold values are presented below.

| Number of days for: | Cyclophosphamide 3g/m$^2$ followed by filgrastim 5μg/kg | filgrastim 5μg/kg + SCF 5μg/kg | filgrastim 5μg/kg + SCF 10μg/kg | filgrastim 5μg/kg + SCF 15μg/kg | filgrastim 5μg/kg + SCF 20μg/kg | p-value* |
|---|---|---|---|---|---|---|
| CFU-GM >5x10$^3$/ml | 5 (0-7) | 4 (2-5) | 5 (1-8) | 8 (5-10) | 9 (6-10) | p<0.001 |
| BFU-E >5x10$^3$/ml | 5 (0-7) | 5 (2-5) | 6 (2-9) | 7 (6-10) | 9 (6-10) | p<0.001 |
| CD34+ >50x10$^3$/ml | 3.5 (0-5) | 3 (0-5) | 4 (0-8) | 6 (3-9) | 7 (6-8) | p<0.001 |

*Linear regression on SCF dose across all 5 groups

The greatest number of days above threshold levels was observed for those patients receiving SCF 20μg/kg plus filgrastim for all parameters. This has an important clinical implication in that the higher doses of SCF offer a greater 'window of opportunity' in which to perform the apheresis to achieve high yields.

### O69 DEVELOPING A MODEL FOR NURSE-LED CLINICS WITHIN A RADIOTHERAPY OUTPATIENT DEPARTMENT

D Dodwell, J Campbell, L German, C Coyle, C Lane - Yorkshire Regional Centre for Cancer Treatment, Cookridge Hospital, Leeds, LS16 6QB

**Objective:** To provide a descriptive account of the activities which take place within a radiotherapy review clinic and to compare them with a nurse-led clinic.
**Background:** There are numerous initiatives to identify possibilities for the greater involvement of non-medical practitioners in patient care. Suitably trained nurses already form an important part of the multi-disciplinary team and the enhancement of the role of the specialist nurse in other areas may improve the delivery and quality of care.
**Subjects:** 71 adult patients receiving outpatient radiotherapy and assessed in the traditional (doctor-held) radiotherapy review clinic and 141 patients from one Consultant's practice receiving outpatient radiotherapy and reviewed in a nurse-led review clinic.
**Outcome measures:** These included the activities in both clinics, including the degree and number of interactions that occurred, consultation time, waiting time, degree of involvement from other support services, reasons for doctor contact and perceptions of patients, doctors and radiographers.
**Results:** Nurse consultations lasted significantly longer than those of doctor's. Waiting time was reduced. Of 391 episodes of review, 21 contacts were made with the doctor, predominantly because of the perceived need for a prescription. There were a greater number of referrals and liaison with other support groups, (88 referrals within 391 episodes within the nurse-led clinic, compared to 4 within 71 episodes for the traditional doctor-held clinic). Interactions were divided into 6 broad categories (treatment enquiry, examining radiotherapy site, provision of a prescription, psycho-social enquiry/support, investigation/other procedures and information/advice giving). Significantly more interactions occurred during nurse consultations.
**Conclusion:** This small sequential observational study suggests that once the activities occurring within a traditional radiotherapy review clinic were analyzed, specialist trained nursing staff, given appropriate medical support when required, may undertake the tasks involved in a radiotherapy review clinic equally (and possibly more) effectively.

### O70 FRACTION OF NORMAL REMAINING LIFE: A NEW METHOD FOR EXPRESSING SURVIVAL IN CANCER.

**J S Vaidya*[1], *I Mittra[1]**
*[1]Department of Surgery, Tata Memorial Hospital, Bombay, 400 012, India

The plotting of conventional survival curves for cancer, using the interval between diagnosis and date of last follow up or death to denote survival time, ignores the patient's expected life span had the patient been healthy. In human terms, the impact of a projected 10 year survival of a woman diagnosed to have breast cancer (for example) would be different for a 30 year old patient as opposed to a 70 year old. We believe that survival is better expressed as a fraction of normal remaining life span expected at the time of diagnosis.

To illustrate this concept, we used a database of 1134 operable breast cancer patients from Bombay who had their primary surgery performed at Tata Memorial Hospital between 1974 and 1988. Each patient's age at diagnosis is subtracted from the average life expectancy for that age to obtain what we call her normal remaining life (NRL). At the time of survival analysis, the percentage of NRL that has actually been lived by the patient was calculated and used in place of survival time to plot actuarial survival curves which we 'call real life expectancy curves. The survival curves were plotted in two different ways: by the conventional method and the novel way which we call real life expectancy method. Both curves were plotted using the actuarial method. For example, in India, a healthy 40 year old woman has a normal life expectancy of 72 years and NRL of 32 years (72 minus 40). If at 40 she were diagnosed to have breast cancer, and she lived for 10 years, her survival is expressed as 31% of her NRL (10/32 x100). On the other hand, a similar patient of breast cancer aged 60 at diagnosis would have a normal life expectancy of 75 years and NRL of 15 years. If she lives for 10 years after diagnosis, her survival is expressed as 67% (10/15 x 100) of her NRL. To plot the Real Life Expectancy curves, these percentage figures were used instead of actual number of years. The mathematical procedure and statistical considerations are exactly the same as that used for plotting conventional actuarial survival curves. Using the conventional method, one would estimate that a woman with uninvolved nodes has a 82% chance of living for 10 years while using the new method she would have a 81% chance of living 1/2 of her NRL. Since the normal life expectancy of a 40 year old Indian woman is 72 years and that of a 60 year old is 75 years, this would work out to a 81% chance of living for 16 years for a 40 year old woman and 7.5 years for a 60 year old. We could even say that a node -ve woman has a 68% chance of living her full NRL which is equivalent of a cure. The importance of the facility to express survival in terms of cure, especially for a disease such as cancer, is profound. We believe that by individualising survival estimates according to age and expressing survival in terms of cure rates, the new method that we have proposed makes survival estimates more meaningful, relevant and human.

### O71 INFORMATION RECALLED, HOSPITAL ANXIETY DEPRESSION SCORES (HAD) AND KNOWLEDGE OR CONSENT AMONGST PATIENTS UNDERGOING RADIOTHERAPY (RT)

A P M Lydon[1], C Montgomery[2], A Hong[3], and K Lloyd[4], [1]Department of Oncology, [2] Department of Psychiatry, Royal Devon & Exeter Healthcare NHS Trust, Exeter, EX2 5DW.

Patients may complain of receiving too little or occasionally too much information about their cancer diagnosis and treatment. Our aim was to determine current knowledge of patients undergoing RT, and any association with HAD scores.

**75** patients were interviewed. They were questioned about their satisfaction with information given, knowledge of treatment related side effects, consent and preferred source of information. All completed a standardised HAD score questionnaire.

**58 (77%)** of patients were satisfied with amount of information given. There was a correlation between high HAD scores and dissatisfaction with information recalled (**p = 0.04**). Only **56 (75%)** could remember signing a consent form,
**27** believed they had consented to any treatment, surgery, chemotherapy and RT.
**57 (76%)** recalled acute side effects, considered inappropriate in 3. Only 2 knew of late side effects. **16 (22%)** would have liked more discussion time **14** with their oncologist in preference to their GP, radiographer or patient with a similar illness.

This study is one of the first to suggest that patients predisposed to anxiety and depression, identified by HAD scoring, are more likely to be dissatisfied with information given about their disease and treatment. Many patients would value more time with their oncologist. Late side effects need to be discussed more freely and perhaps included in obtaining consent.

### O72 PATIENTS' EXPERIENCES OF MENOPAUSE -LIKE SYMPTOMS FOLLOWING BREAST CANCER TREATMENT

R Adewuyi-Dalton, J Bradburn, E J Maher and the Breast Cancer & Hormones Information Committee.

**Objectives:** This study explored womens' experiences of menopause-like symptoms after breast cancer treatment. This study was associated with a national R&D study of HRT use. The aim was to assess the impact of symptoms upon their lives, the support and information they found to assist them and current priorities in addressing the symptoms.
**Method:** 46 breast cancer support groups throughout the country volunteered to participate. Focus groups were held with 6 groups to elicit the range of concerns associated with menopause-like symptoms. This data was used as the basis for a questionnaire sent to the remaining 40 groups.
**Results:** All groups replied resulting in 344 evaluable questionnaires.

- Symptoms: hot flushes (80%), tiredness (74%), night sweats (69%), weight gain (67%) and insomnia (58%). Psychological symptoms (loss of concentration, memory loss, depression, mood swings, panic attacks and loss of control) affected up to 50%.
- 62% attributed symptoms to Tamoxifen, 17% a natural menopause.
- Of those experiencing hot flushes 52% were told to expect them; for loss of concentration 13%. Information sources for advice on symptoms were diverse; 58% asked their GP, 42% a breast care nurse, 8% a gynaecologist, 36% a support group member and 33% an oncologist. Advice was useful in understanding symptoms but not to relieve them.
- Dealing with symptoms: 12% considered their symptoms were of sufficient severity to take HRT if offered. Opinion indicated that 78% would only take HRT if cancer doctors monitored closely. Self-help strategies were prioritised by 71% and improved information by 65%.

**Conclusions:** The study highlights the extent of physical and psychological distress as a result of menopause-like symptoms and the lack of acknowledgement of this in terms of the information and support offered. A patient information booklet addressing this need is currently being produced by a multi-disciplinary lay/professional group.

### O73

SUCCESS AND COMPLICATIONS OF ENDOVASCULAR STENT INSERTION IN THE MANAGEMENT OF THE SVC (SUPERIOR VENA CAVA) SYNDROME.

S.M. Cooper, E.H. Whitby, P.A.Gaines, T.J.Cleveland, Sheffield Vascular Institute, Northern General Hospital, Sheffield S5 7AU, U.K

OBJECT: To assess the success and complications of endovascular stent insertion in the treatment of SVC syndrome in a large series of patients.
METHODS: Of 63 patients 61 had mediastinal malignancy; 40 related to bronchial tumours, 19 breast carcinoma, 2 other tumours. Two had benign disease. Forty had undergone previous radiotherapy. Thirty-three patients had total venous occlusion with 17 requiring thrombolysis or clot aspiration prior to stenting. The remainder had uncomplicated stenoses of the SVC or brachiocephalic veins. Twenty-one patients required multiple stents. Forty-eight were given Heparin during and immediately post-procedure with 19 continuing long-term anticoagulation.
RESULTS: 98% patients experienced rapid symptomatic relief. In 55 normal vessel calibre was restored, with partial recanalisation in 8. Eight required further treatment (4 thrombolysis, 4 additional stents), all successfully. Survival ranged from 5 days to over 9 months, 40% died within 1 month. Complications occurred in 8 (12.7%); 6 related to anticoagulation (2 to thrombolysis, 2 to heparin post-procedure, 2 to long-term anticoagulation), 1 technical failure, 1 wound cellulitis. No deaths were attributable to the procedure.
CONCLUSION: Stenting is a safe and effective treatment providing almost immediate symptomatic relief. It should be available to all patients with the SVC syndrome.

### O74

PALLIATION OF MALIGNANT DYSPHAGIA WITH EXPANDING METALLIC STENTS.

G J O'Sullivan, J L Mansi, J P Glees and A Grundy. Depts of Radiology & Oncology, St George's Hospital, London SW17 0QT.

Palliation of symptoms and complications of stent insertion to relieve malignant dyspagia have been retrospectively studied.

98 expanding metal stents have been inserted in 87 patients (61 male, 26 female). Age ranged from 46.2 to 92.7 years (mean 73 years). 69 patients had primary tumours of the oesophagus, 6 patients had recurrent disease following previous surgery or radiotherapy and 12 patients had extrinsic involvement by other malignant disease. 74 Strecker stents, 22 covered Wallstents, 1 Gianturco and 1 Instent were used.

Three patients died within 3 days of stent insertion. Immediate relief of dysphagia was achieved in 80 of the remaining 84 patients. Three fistulas to the tracheo-bronchial tree were sealed with covered stents. There were no major complications related to stent insertion. Patient survial ranged from 1 day to 2 years with a mean survival of 7 weeks. Major late complications were food impaction in 5 patients, stent migration in 3, minor bleeding in 2 and the development of an oesophago-aortic fistula in one patient. 8 patients had tumour overgrowth or ingrowth managed by insertion of a second stent or laser ablation.

Although complications may occur in about 20% of patients, expanding metal stents provide good palliation of malignant dysphagia allowing over 90% of patients to maintain oral feeding.

### O75

WHY DO PATIENTS WITH WEIGHT LOSS HAVE A WORSE OUTCOME WHEN UNDERGOING CHEMOTHERAPY FOR GASTRO-INTESTINAL MALIGNANCY?

**H.J.N.Andreyev, P.J.Ross, A.R. Norman, J. Oates and D. Cunningham** GI Unit, Royal Marsden Hospital & CRC Centre of Cancer Therapeutics, Institute of Cancer Research, Sutton, Surrey.

**Aims:** To examine whether weight loss at presentation for chemotherapy for gastrointestinal carcinomas influences outcome and whether nutritional intervention could be worthwhile.

**Methods:** Data were gathered prospectively. The outcomes were compared of patients with or without weight loss treated for locally advanced or metastatic tumours of the oesophagus, stomach, pancreas, colon or rectum.

**Results:** Of 1,555 consecutive patients included, weight loss was reported more commonly by men (51%) than women (44%) at presentation ($p=0.01$) Patients with weight loss received less chemotherapy but developed more frequent and more severe dose limiting toxicity - plantar-palmar syndrome ($p<0.0001$) and stomatitis ($p<0.001$) - than patients without weight loss. Consequently, patients with weight loss on average received 1 month less treatment ($p<0.0001$). Weight loss correlated with shorter failure free ($p<0.0001$, Hazard Ratio=1.25) and overall survival ($p<0.0001$, Hazard Ratio=1.63), decreased response ($p=0.006$), quality of life ($p<0.0001$) and performance status ($p<0.0001$). Patients who stopped losing weight had better overall survival ($p=0.0004$). Weight loss at presentation was an independent prognostic variable (Hazard Ratio =1.43).

**Conclusions:** The poorer outcome from treatment in patients with weight loss appears to occur because they develop more toxicity, have more breaks from treatment and receive significantly less chemotherapy rather than any specifically reduced tumour responsiveness to treatment. Randomised nutritional support intervention studies are urgently required in these groups of patients.

### O76

NON-SURGICAL TREATMENT FOR CHONDROSARCOMA

MQF Hatton, J Reid, S Murray*, R Reid+, A Barrett. Beatson Oncology Centre, Western Infirmary, Glasgow G11 6NT. *Dept. Orthopaedic Surgery, Western Infirmary, Glasgow. +Scottish Bone Tumour Registry, Dept. of Pathology, Western Infirmary, Glasgow.

We present a retrospective review of patients with chondrosarcoma, from the records of the Scottish Bone Tumour registry, who received radiotherapy or chemotherapy as part of their treatment. There were 65 patients in this group in the 305 patients with chondrosarcoma identified in Scotland between 1960 and 1995. Thirty five were male, the median age was 57 years (range 5 - 89).

All patients had histologically confirmed chondrosarcoma, (13 grade 1, 19 grade 2, and 27 grade 3). Forty nine patients had tumours arising in the axial skeleton; commonest sites being pelvis (23), ribs (11), femur (9), humerus (7), spine (6) and skull (4).

37 patients received radiotherapy as their primary treatment, a further 14 as an adjuvant to surgery and 19 as treatment for relapse. Doses ranged from a 10 Gy single treatment to 73 Gy in 33 fractions. One patient received primary chemotherapy, 9 adjuvant and 15 chemotherapy for relapse.

13 patients (35%) receiving primary radiotherapy had a partial response, 22 stable disease and 2 progressed on treatment; the median time to relapse was 7 months (range 0 - 198). Palliative radiotherapy produced 1 complete and 3 partial responses (21%), 10 patients had stable disease, 4 progressed with 1 unassessed for response. The median time to relapse was 3 months (range 0 - 54). The median survival for all patients following radiotherapy was 11 months (range 1 - 232).

Twenty four patients received chemotherapy, 13 were given a single agent, 11 combination treatment. Overall 3 patients had a partial responses, 8 stable disease, 9 progressed on treatment and in 5 the response could not be assessed. The median time to relapse was 3.5 months (range 0 - 14) and median survival following treatment was 8 months (range 1 - 30).

In conclusion, this review suggests a role for radiotherapy in the palliation of chondrosarcomas that cannot be adequately treated surgically. However, the results seen with chemotherapy are very disappointing and the use of this treatment cannot be recommended outside a trial setting.

### O77 PROSPECTIVE VALIDATION OF MRC PROGNOSTIC INDEX IN HIGH GRADE GLIOMAS.

R.Benson, S.Old, L.T.Tan, S.Harden, V.R.Bulusu, N.Burnet, Oncology Centre, Addenbrooke's Hospital, Cambridge, U.K. CB2 2QQ.

The importance of prognostic factors in patients with high grade gliomas has been emphasised in previous MRC studies. Age, WHO performance status, length of history of fits and extent of surgery were identified as independent prognostic variables. A prognostic index was developed to predict survival.

**Aim**: To prospectively evaluate the MRC prognostic index in patients with high grade gliomas.

**Methods**: MRC prognostic index was recorded in 62 adult patients with high grade gliomas (WHO Gr 3&4). 54 pts received radiation, 28 had 60 Gy in 6wks, 14 had 45Gy in 4wks, 12 had 36Gy in 2.5wks to their tumour volume using 5-8Mv photons. 8 pts had biopsy only or partial excision. Patients with MRC prognostic index of <25 received radical radiotherapy and those with index of >25 either had a palliative (36Gy) dose or were treated symptomatically. All patients were followed upto relapse/death. Three prognostic groups were created based on the index, Group I (n=16) score=0-15, Group II (n=20) score=16-25 and Group III (n=26) score=>26. Mean (95% C.I) and median survival were calculated for all three groups.

**Results**: Survival data for the 3 Prognostic Groups

| Group | MRC prognostic index | Expected survival | Observed Survival(wks) Mean | Median | 95%C.I. |
|---|---|---|---|---|---|
| I n=16 | 0-15 | 51-80wks | 147 | 126.5 | 126.5-167 |
| II n=20 | 16-25 | 33-46wks | 48.4 | 43.5 | 36.5-60 |
| IIIn=26 | >25 | 16-28wks | 17 | 17 | 13-21 |

**Conclusions**: MRC prognostic index, though a useful prognostic tool, tends to underestimate survival in the best prognostic group (score <15), especially in patients with scores <10. It probably overestimates survival in the poor prognostic group (score >25).

### O78 ANTI-TUMOUR IMMUNE RESPONSES AFTER MONOCLONAL ANTIBODY THERAPY OF OVARIAN CANCER,

S. Nicholson[1,2], H.Thomas[1], D.Snary[2],J. Taylor-Papadimitriou[2]. [1]Dept of Clinical Oncology, Hammersmith Hospital, London; [2]Imperial Cancer Research Fund, Lincoln's Inn Fields, London.

Two multi-centre trials are underway exploring the role of monoclonal antibody HMFG1 in patients with ovarian carcinoma: patient accrual is ongoing in a phase III trial of radiolabelled antibody and in a phase I/II trial of unlabelled HMFG1. The target is the tumour-associated antigen Polymorphic Epithelial Mucin or "MUC-1."

Methods for assessing successful immune stimulation are essential, both to understand the mechanism behind any anti-tumour effect of these treatments, and to correlate immune response with clinical effectiveness. The latter is important, as these approaches to treatment are most likely to find clinical application as consolidation therapy in patients with minimal residual disease after chemotherapy. Assays for enhanced immune responsiveness to the target antigen have been performed.

Humoral immune responses comprise general Human Anti-Mouse Antibodies (HAMA), and more specific anti-idiotypic (Ab2) and anti-anti-idiotypic (Ab3) responses. The antigen binding site of Ab3 should bind to the same epitope as the administered MAb. Assays have been developed for measuring Ab2 and Ab3 responses in our patients. As measured by ELISA, Ab2 signal was increased in 10/10 recipients of adjuvant intraperitoneal radioimmunotherapy and Ab3 was increased in 6/10 patients.

Cellular responses to the antigen have been studied by measuring the proliferation of peripheral blood lymphocytes to peptides which possess the epitope recognised by the HMFG1 antibody. Lymphoproliferation has been measured prior to antibody therapy and at fixed timepoints thereafter. Enhanced proliferation has been demonstrated in 2/6 patients after adjuvant intraperitoneal radioimmunotherapy and 4/10 patients treated with multiple doses of unlabelled antibody on an idiotypic vaccination protocol.

Treatment strategies will be outlined, methods of immune assessment will be described in more detail and updated clinical and immunological results will be presented.

### O79 RADIOIMMUNOTHERAPY OF BCL-1 LYMPHOMA WITH IODINE-131 LABELLED ANTIBODIES : DETERMINANTS OF SUCCESSFUL THERAPY

**T. M. Illidge; R.Reid; and M. G. Glennie. Lymphoma Research Laboratory, Southampton General Hospital, Southampton S016 6YD.**

The important determinants of effective radioimmunotherapy of the mouse lymphocytic lymphoma BCL-1 have been evaluated by comparing the effects of 131-antibodies in vivo. In contrast, to the largely disappointing results in solid tumours, the use of radioimmunotherapy for the treatment of relapsed B cell Non-Hodgkin's lymphomas appears to be an increasingly promising approach; with impressive durable reported response rates. However, the mechanisms behind these encouraging clinical responses remains largely undetermined, which has meant that the optimal treatment approach including the relative importance of the specific target antigen and the required dose of radioactivity is unknown.

Here we compare antibodies against the invariant chain Ii (CD74) versus tumour specific anti-idiotype and isotype matched control antibodies with and without conjugated Iodine-131. Extensive biodistribution studies with Iodine-125 labelled antibodies enabled accurate organ dosimetric predictions to be made before low dose Iodine-131 (3.7-7.4 MBq) radioimmunotherapy was performed. In contrast to recent reports in the same BCL-1 model we were able to demonstrate a very high therapeutic efficacy (>93% cure) with $10^6$ and $10^7$ cells using $^{131}$-I anti-CD74 and no observed toxicity. The 131-I labelled anti-idiotype was capable of inducing very significant increases in life-span but no cures were observed. We have demonstrated for the first time in an animal model of B-cell lymphoma the importance of delivering radioactivity to the tumour bearing organs by the selection of an antigen target that does not modulate significantly.

Using a range of external beam irradiation doses in the same lymphoma *in vitro* cell line we have been able to demonstrate the exquisite radiosensitivity of this lymphoma is secondary to the ability to trigger rapid and large amounts of apoptosis which can be modified by antibody binding. We suggest that the successful use of RIT in the clinic is due to the ability of this approach to deliver systemic irradiation to the tumour and that the radiation and antibody may induce apoptosis.